# The Genetic and Environmental Origins of Learning Abilities and Disabilities in the Early School

**DOI:** 10.1111/j.1540-5834.2007.00439.x

**Published:** 2007-12

Despite the importance of learning abilities and disabilities in education and child development, little is known about their genetic and environmental origins in the early school years. We report results for English (which includes reading, writing, and speaking), mathematics, and science as well as general cognitive ability in a large and representative sample of U.K. twins studied at 7, 9, and 10 years of age. Although preliminary reports of some of these data have been published, the purpose of this monograph is to present new univariate, multivariate, and longitudinal analyses that systematically examine genetic and environmental influences for the entire sample at all ages for all measures for both the low extremes (disabilities) and the entire sample (abilities).

English, mathematics, and science yielded similarly high heritabilities and modest shared environmental influences at 7, 9, and 10 years despite major changes in content across these years. We draw three conclusions that go beyond estimating heritability. First, the abnormal is normal: Low performance is the quantitative extreme of the same genetic and environmental influences that operate throughout the normal distribution. Second, continuity is genetic and change is environmental: Longitudinal analyses suggest that age-to-age stability is primarily mediated genetically, whereas the environment contributes to change from age to age. Third, genes are generalists and environments are specialists: Multivariate analyses indicate that genes largely contribute to similarity in performance within and between the three domains—and with general cognitive ability—whereas the environment contributes to differences in performance.

These conclusions have far-reaching implications for education and child development as well as for molecular genetics and neuroscience.

## I. INTRODUCTION

Why do children differ so much in their progress in the early school years? For the past half-century, environmental factors have been the prime focus, such as characteristics of schools (e.g., physical facilities, teacher training, discipline systems), neighborhoods (e.g., poverty, crime, pollution), and families (e.g., parental education, use of language, disciplinary practices). Far less attention has been given to the possibility of genetic influences on characteristics of children that affect academic learning (other than IQ) or, more intriguingly, genetic mediation of the effects of schools, neighborhoods, and families (Wooldridge, 1994). Decades of research on the nature and nurture of children's development in families have led to a consensus in developmental psychology that recognizes the importance of genetics as well as environment (Plomin, 2004). However, this fundamental issue of the interplay of nature and nurture has just begun to be addressed in relation to education (Plomin & Walker, 2003). One goal of this monograph is to consider the nature–nurture issue in the early school years in relation to individual differences in performance in reading, mathematics, and science as well as general cognitive ability, which we refer to as learning abilities and disabilities.

However, our main goal is to go beyond this rudimentary nature–nurture question to investigate three issues that have far-reaching ramifications for the field of education because they move toward the question of “how” rather than “how much.” All three issues concern the etiology of relationships between things: between the normal (learning abilities) and the abnormal (learning disabilities), between ages (7, 9, and 10 years), and between learning abilities and disabilities (English, mathematics, and science) as well as their relationship to general cognitive ability. In short, they are questions of cognitive developmental architecture. The first issue, the relationship between learning abilities and learning disabilities, requires a sample large enough to assess abnormal development in the context of normal development. The second issue, genetic and environmental influences on change and continuity, requires longitudinal data during the early school years. The third issue requires multivariate data on learning abilities and disabilities. Answers to all of these questions require a genetically sensitive design such as the twin method that capitalizes on the experiment of nature provided by identical and fraternal twins as described in Chapter II.

These requirements are met by the Twins' Early Development Study (TEDS), a large and representative sample of twins whose progress during the early school years has been assessed longitudinally at 7, 9, and 10 years (Oliver & Plomin, 2007; Trouton, Spinath, & Plomin, 2002). Some of these results have been reported previously in diverse literatures such as education, learning disabilities, and language in addition to child development (Oliver & Plomin, 2007). As is necessarily the case with longitudinal projects, these papers were published as data collection progressed during the course of the 10-year TEDS project, often at one age using different samples, models, and analytic strategies. Our goal here was to examine genetic and environmental influences systematically in univariate, multivariate, and longitudinal analyses that are based on the entire sample at all ages for all measures for both the low extremes (disabilities) and the entire sample (abilities). All of the analyses in this monograph are new and based on the same complete dataset and the same models and analytic strategies at 7, 9, and 10 years. We found that new interpretations emerged from comparisons across measures and ages that were not apparent in previous analyses focused on one measure or one age.

In this chapter, we provide a brief overview of what is known about the genetic and environmental origins of learning abilities and disabilities in the early school years. In addition to the basic issue of nature and nurture, we introduce the three themes of this monograph: the etiological relationship between the normal (abilities) and abnormal (disabilities), genetic and environmental contributions to longitudinal continuity and change, and multivariate analyses of genetic and environmental heterogeneity and homogeneity.

In this monograph, we use the phrase *learning abilities and disabilities* but not to indicate an a priori position on the issues of achievement versus ability and nature versus nurture. We view achievement and ability as a continuum from learning specific skills and content (e.g., learning to read) to using these skills and contents for comprehension and problem solving (reading to learn). Especially strong views are held on the use of appropriate labels for children's low performance, with the pros and cons debated for such labels as challenge, delay, difficulty, disorder, and impairment. We use the word *disability* with its semantic link to the word *ability* because research presented in this monograph suggests that common learning disabilities are the low end of the normal distribution of learning abilities (Chapter IV).

Finally, we recognize that there are many possible ways to address the questions that we raise in this monograph. Moreover, there are many other possible questions that can be asked about this dataset. For this reason, all of the data included in this monograph are freely accessible as a zipped SPSS file at the following web page: http://www.teds.ac.uk/information/SRCDdataset.htm. The academic and cognitive measures are included in standardized form (following adjustment for sex and age at time of assessment as in our genetic analyses) and unstandardized form. Only data described in this monograph are included in the dataset and these data are only available at the level at which they are analyzed in this monograph. For example, in this monograph we analyze data at the level of scales rather than individual items. It is our hope that Chapter II will provide adequate annotation for use of the dataset.

### LOGIC AND ASSUMPTIONS OF THE TWIN METHOD

Adoption and twinning provide naturally occurring experimental situations that illuminate the relative influence of nature and nurture on specific traits and on the relationship between traits. Although each method has its own limitations (described in detail elsewhere, e.g., Plomin, DeFries, McClearn, & McGuffin, in press), these limitations are generally complementary. Consequently, the convergence of results from the two methods provides strong evidence for the validity of the findings. This section provides a brief introduction to the logic of the twin method; more information on statistics and estimates is presented in Chapter II and in the following chapters on results. The method is based on comparison between identical and nonidentical twins. Identical or monozygotic (MZ) twins derive from one zygote and are genetically identical. If genetic factors are important for a trait, these genetically identical pairs of individuals must be more similar than first-degree relatives, who are only 50% similar genetically on average. The best comparison group among first-degree relatives for MZ twins is dizygotic (DZ) twins, who develop from separately fertilized eggs. Half of DZ twins are same-sex pairs and half are opposite-sex pairs. Like MZ twins, DZ twins experience together most prenatal and many postnatal experiential variables such as prenatal nutrition and family social class.

If a trait is influenced genetically, identical twins must be more similar than fraternal twins. However, when greater similarity of MZ twins is found, it is also possible that some or all of the greater similarity is caused environmentally rather than genetically. The equal environment assumption of the twin method assumes that environmentally caused similarity is roughly the same for both types of twins. If the assumption were violated because identical twins experience more similar environments and consequently develop more similarly than nonidentical twins, this violation would inflate estimates of genetic influence. There is, in fact, evidence that MZ twins are treated more similarly than their DZ counterparts. For example, as children, MZ twins are more likely to have the same playmates, share the same room, and dress alike. As adults, MZ twins are more likely to keep in contact than are same-sex DZ twins (Evans & Martins, 2000). However, the equal environment assumption would only be violated if this greater similarity for MZ twins leads to a greater similarity for phenotypes of interest. The equal environments assumption has been tested in several ways and appears reasonable for most traits. For example, environmental similarity during childhood does not predict twin similarity in personality, attitudes, intelligence, nor a range of psychiatric disorders (Evans & Martins, 2000). Moreover, both greater similarity of parental treatment of MZ twins and greater physical similarity between MZ twins are uncorrelated with twin similarity for personality, vocational interests, and cognitive abilities.

Another potential violation of the equal environment assumption, in the opposite direction from that just discussed, would occur if identical twins experience greater environmental differences than fraternal twins, such as greater prenatal competition. To the extent that identical twins experience less similar environments, the twin method will underestimate heritability. Despite some potential limitations, the twin study remains the best method for assessing the relative contribution of genes and environment to traits in human populations (Evans & Martin, 2000). However, it is important to remember that statistics derived from twin data, which estimate genetic influences (heritability) and environmental influences, have very specific definitions within the twin method (see Chapter II), and can be misinterpreted. For example, heritability refers to effect size, the extent to which individual differences for the trait in the population can be accounted for by genetic differences among individuals. Effect size in this sense refers to individual differences for a trait in the entire population, not to the effect of genetic factors on a specific individual (Plomin et al., in press). In other words, heritability is the proportion of phenotypic variance that can be accounted for by genetic differences among individuals. Like all statistics, heritability estimates include error of estimation, which is a function of the effect size and the sample size. Therefore, as with other methods, replication across studies and across designs is crucial.

It should be emphasized that heritability refers to the contribution of genetic differences to observed differences among individuals within a specific population, for a particular trait, and at a particular time. Moreover, heritability describes *what is* in a particular population at a particular time rather than *what could be*. That is, if either genetic influences change (e.g., changes due to migration) or environmental influences change (e.g., changes in curricula or in educational opportunity), then the relative impact of genes and environment will also change. Even for a highly heritable trait such as height, changes in the environment could make a big difference. For example, if an epidemic struck or if children's diets were altered for the worse by famine, average height would decrease, but genetic influence might actually increase due to diminished environmental variance.

We also emphasize that the causes of individual differences within groups have no implications for the causes of average differences between groups. Specifically, heritability is defined, both conceptually and statistically, as the genetic contribution to differences among individuals within a group. Differences between groups may have quite different causes, which are difficult to evaluate rigorously; twin studies have little use here. Finally, it is important to remember that genetic influence on behavior involves probabilistic propensities rather than predetermined programming.

Much of the research reported in this monograph takes advantage of important extensions of the basic, univariate twin method. The most important is the development of multivariate methods (Martin & Eaves, 1977). The univariate approach just described estimates the genetic and environmental contribution to the variance in a specific trait. Analogously, multivariate methods estimate the genetic and environmental contribution to the covariance, or correlation, between two traits. Many aspects of behavior and development are known to be phenotypically correlated; however, such correlations might be the result of shared genetic influence or environmental influences on both. Distinguishing those influences provides valuable insight into the mechanisms underlying each. Like univariate analyses, multivariate analyses contrast correlations for MZ and DZ twins, where the magnitude of the discrepancy between them indexes a genetic effect, and the magnitude of the correlations regardless of zygosity indexes a shared environmental effect. But in multivariate analyses, the relevant correlation is the cross-trait twin correlation, that is, correlating measure A for twin 1 with measure B for twin 2. Multivariate analyses also provide an estimate of the degree to which the same genetic influences are at play for two traits. A special case of multivariate analysis of particular interest to developmental science is longitudinal analysis, where measure A and measure B (possibly the same measure, possibly a different one) are obtained at different time points. Longitudinal genetic analyses estimate genetic and environmental contributions to continuity and change. The assumptions underlying the twin method, and the qualifications concerning interpretation, apply to multivariate and longitudinal as well univariate analyses.

### NATURE AND NURTURE OF LEARNING ABILITIES

In this section, we focus on individual differences in learning abilities throughout the normal distribution. In the next section, we review research relevant to low performance because the genetic and environmental etiology of abilities and disabilities can differ; this issue is the focus of Chapter IV.

The first twin study with test data on academic performance in childhood included 278 pairs of twins that ranged in age from 6 to 12 years (Thompson, Detterman, & Plomin, 1991). The published report indicated modest heritability (about 20% of the variance in test performance was accounted for by genetic influences) and substantial (about 60%) shared environmental influence (i.e., environmental effects shared by the twins), but the measures had not been corrected for age. Age correction is necessary because members of a twin pair are exactly the same age; failure to correct for age inflates estimates of shared environment (see Chapter II). With age correction, the results of this study suggest moderate heritability (about 40%) and moderate shared environmental influence (about 40%) (L. A. Thompson, personal communication, June 21, 2006).

Three other twin studies of a broad range of academic abilities have been reported for older children. The classic study in this area included bright high school–age twins in the United States, using data obtained from the National Merit Scholarship Qualifying Test for 1,300 MZ and 864 DZ twin pairs (Loehlin & Nichols, 1976). For English and mathematics, MZ twin correlations were about .70 and DZ correlations were about .50, again suggesting moderate heritability (about 40%) and shared environmental influence (about 30%). The second study, which included 190 twin pairs assessed on a Dutch national test of educational achievement at 12 years, reported greater heritability (about 60%) and similar shared environmental influence (about 30%) (Bartels, Rietveld, van Baal, & Boomsma, 2002b). The third study yielded yet another pattern of results (Wainwright, Wright, Luciano, Geffen, & Martin, 2005b). The study of 390 pairs of twins from 15 to 18 years reported substantial heritability (about 60%) and modest shared environmental influence (about 10%). However, rather than assessing achievement in particular subjects such as English, the tests used in this latter study assessed general cognitive abilities such as “comprehension of facts from a broad range of stimuli” and “deduction and induction among relationships” (p. 603).

Given how diverse the studies are in samples, ages and measures, their results are surprisingly consistent in suggesting at least moderate heritability (about 50% on average) and shared environmental influence (about 25%). All these studies were based on tests administered to the twins. Another result relevant to findings in this monograph comes from an early Swedish study of a thousand pairs of 13-year-old twins based on report card grades (Husén, 1959). Results for reading, writing, and arithmetic were similar: The average heritability was 50% and the average estimate of shared environmental influence was 30%. The consistent evidence for shared environmental influence would seem unremarkable except for the striking fact that little evidence has been found for shared environmental influence in other domains of behavioral development such as personality or psychopathology (Plomin, Asbury, & Dunn, 2001).

Reading has received the most attention among academic abilities in genetic research. The major twin study of reading is a Colorado study that focused on reading disability but also included a control sample of twins (Light, DeFries, & Olson, 1998). For the control sample of 223 pairs of twins from 8 to 20 years of age, individual differences in reading ability yielded moderate heritability (about 40%) and modest shared environment (about 25%). A review of five smaller twin studies of various measures of reading ability in childhood also suggests an average heritability estimate of about 40% but the shared environment estimate was much higher, about 45% (Stromswold, 2001). Two more recent studies included many measures of early reading, although the sample sizes were modest (Byrne et al., 2005; Petrill, Deater-Deckard, Thompson, Schatschneider, & DeThorne, in press). The studies found diverse results across measures but generally suggested moderate genetic and shared environmental effects.

For mathematics, the only genetic research other than the three studies mentioned above comes from the Colorado study of reading, which also included tests of mathematics. High heritability (69%) and negligible shared environmental influence (6%) were reported for mathematics ability (Light, DeFries, & Olson, 1998); a latent variable analysis yielded even higher heritability and negligible shared environment (Alarcón, Knopik, & DeFries, 2000). For science, the only genetic study is the study of bright high school students mentioned above which reported heritability of 40% and shared environment of 30% for a measure of critical reading of scientific material (Loehlin & Nichols, 1976). Although science is not one of the traditional educational domains—reading, writing, and arithmetic—science has increasingly become a focus for education. For example, in the United Kingdom, science became a compulsory subject in elementary teaching in 1989 with the introduction of the National Curriculum.

In contrast to the meager previous research on academic learning abilities, a massive amount of research has been conducted on general cognitive ability (“*g*”), which refers to the observed positive manifold among different cognitive (verbal and nonverbal) tasks (Plomin & Spinath, 2004). This research has been reviewed many times, including an influential review in *Science* (Bouchard, Jr. & McGue, 1981). An updated review yielded an average MZ twin correlation of .86, which is near the test–retest reliability of the measures, in contrast to the DZ correlation of .60 (Plomin & Petrill, 1997). This pattern of twin correlations again suggests heritability of about 50% and shared environmental influence of about 30%. Meta-analyses including all of the family, adoption and twin data on “*g*” also yield heritability estimates of about 50% (Chipuer, Rovine, & Plomin, 1990; Devlin, Daniels, & Roeder, 1997; Loehlin, 1989). Similar results continue to be found in more recent twin studies (Benyamin, Wilson, Whalley, Visscher, & Deary, 2005; Rietveld, Dolan, van Baal, & Boomsma, 2003; Wainwright et al., 2005b). However, this overall conclusion averages out two important developmental changes, as discussed later.

### NATURE AND NURTURE OF LEARNING DISABILITIES

Even fewer genetic studies have addressed the nature and nurture of learning disabilities. It cannot be assumed that low performance is influenced quantitatively and qualitatively by the same genetic and environmental factors responsible for the normal distribution of variation in learning abilities. The same issues about etiology are relevant to the origins of high ability but they are beyond the scope of the present paper and have only been addressed in relation to high “*g*” (e.g., Ronald, Spinath, & Plomin, 2002).

In fact, twin studies of learning disabilities suggest results roughly similar to those for learning abilities. For example, a review of twin studies of learning disabilities reported twin concordances (the likelihood that one twin will be affected if the other twin is affected) of 75% for MZ twins and 43% for DZ twins for language disability and 84% and 48%, respectively, for reading disability (Stromswold, 2001). For mathematics disability, the concordances are about 70% for MZ twins and 50% for DZ twins (Oliver et al., 2004). No twin studies of low performance in the sciences have been reported.

Treating low performance categorically, that is, analyzing twin data dichotomously as normal versus not normal, loses information about quantitative variation in the normal distribution. As explained more fully in Chapter II, we have emphasized an analysis called DF extremes analysis that combines qualitative information about probands' low performance with quantitative variation in their cotwins. Using DF extremes analysis, a review of twin studies that reported results for both learning disabilities and abilities found that the average weighted “group” heritability was .43 for language disabilities and .25 for language abilities; .52 and .63 for reading disabilities and abilities, respectively; and .61 and .63 for mathematics disabilities and abilities (Plomin & Kovas, 2005). In these analyses, group heritability refers to genetic influence on the average difference between the low-performing group and the rest of the population. However, most of these were small studies that make it hazardous to compare the magnitude of genetic influence for disabilities and abilities, a comparison that makes daunting demands in terms of sample size for adequate statistical power. Despite the large number of twin studies of individual differences in “*g*,” there are scarcely any twin studies on low “*g*” or mental retardation (Spinath, Harlaar, Ronald, & Plomin, 2004).

One of the goals of the present monograph is to investigate genetic and environmental influences on learning abilities in a large and representative sample, which is the focus of Chapter III. For the first time, we systematically compare estimates of genetic and environmental influences on individual differences across the full distribution of ability with estimates of those influences for low-performing children within the same sample assessed on the same measures at the same ages. The first question is whether the magnitude of genetic and environmental influences is similar for learning abilities and disabilities. However, as explained in Chapter II, even if the magnitude of genetic and environmental influence is the same for disability and ability, completely different genetic and environmental factors could be responsible for the genetic and environmental influence. A feature of DF extremes analysis is that, by combining qualitative information about proband status and quantitative variation in cotwins, it can clarify genetic and environmental links between the abnormal and the normal. This second question is the focus of Chapter IV.

### DEVELOPMENTAL CONTINUITY AND CHANGE

To what extent do genetic and environmental influences on learning abilities and disabilities change during development? There are two questions here—a question about quantitative differences in the magnitude of genetic and environmental influences and a question about qualitative changes in genetic and environmental influences. The first question about quantitative differences can be addressed with cross-sectional data. The second question about qualitative changes from age to age requires longitudinal data.

Concerning quantitative age differences, genetic research on “*g*” has yielded two fascinating developmental trends. First, heritability increases linearly from about 20% in infancy, to about 40% in middle childhood, to about 50% in adolescence and young adulthood, and even higher in middle age (Boomsma, 1993; McGue, Bouchard, Jr., Iacono, & Lykken, 1993; Plomin, 1986). The cause of this developmental increase in heritability is not known but one possibility is that as children increasingly make their own way in the world they move from experiencing environments largely created by other people to actively creating correlations between their genetic propensities and their experiences (Plomin & DeFries, 1985). Second, shared environmental influence decreases sharply from about 30% in childhood to near 0% in adolescence, perhaps as adolescents increasingly live their lives outside their family. To the extent that academic achievement reflects “*g*,” similar developmental trends would be expected for learning abilities and disabilities.

Although there are few studies of learning abilities, and their measures and samples differ considerably, the results reviewed in the previous section on learning abilities suggest a trend in this same direction. The only study in the early school years (middle childhood) yielded estimates of 40% heritability and 40% shared environment (Thompson et al., 1991). In early adolescence, two studies yielded average estimates of about 55% heritability and 30% shared environment (Bartels et al., 2002b; Husén, 1959). In late adolescence, two studies yielded average estimates of about 50% heritability and 20% shared environment (Loehlin & Nichols, 1976; Wainwright, Wright, Geffen, Luciano, & Martin, 2005a; Wainwright et al., 2005b). Nonetheless, although such cross-sectional comparisons across studies with different samples and measures can provide rough estimates of developmental differences in genetic and environmental influences, what is needed for precise comparisons is a longitudinal study with the same samples and measures at each age. It should also be noted that comparing genetic and environmental estimates across ages requires large samples. For example, in a sample of 200 pairs of twins, a heritability estimate of 40% is surrounded by a 95% confidence interval of 5–70%, which means that it has no power to compare heritability estimates with another study. Twin studies are especially underpowered to detect and compare estimates of shared environmental influence (Hopper, 2000).

The second question about qualitative changes in genetic and environmental influence from age to age requires longitudinal data. Rather than asking how much do genetic and environmental factors affect performance, here we are asking a logically independent question: To what extent are the same genetic and environmental factors influential at different ages? Only one genetically informative study has examined reading longitudinally over more than a 1-year interval. In the Colorado Adoption Project, word recognition was examined at 7, 12, and 16 years in a sample of adoptive and nonadoptive sibling pairs (Wadsworth, Corley, Hewitt, & DeFries, 2001; Wadsworth, Corley, Plomin, Hewitt, & DeFries, 2006). Longitudinal genetic analysis (see Chapter II) indicated that genes were largely responsible for the substantial stability from age to age. Moreover, genetic correlations from age to age—an index of the extent to which it is the same genetic factors that are operative across age—were 1.0 indicating that the same genetic factors affect reading performance from childhood to adolescence. (See Chapter II for descriptions of these analyses.) These rare data for adoptive and nonadoptive siblings are especially important because, unlike twin analyses, adoptive sibling correlations provide a direct test of the importance of shared environmental influence. The results indicate that, although shared environmental influence accounted for only 10% of the total variance in word recognition, all of this shared environmental influence contributed to continuity from age to age. Nearly all of the change from age to age could be attributed to nonshared environment, that is, environmental effects that are distinct for the siblings, not shared.

Two other longitudinal studies of early reading are in progress but as yet have only reported longitudinal analyses from kindergarten to first grade (Byrne et al., 2006; Byrne et al., 2005) or from first to second grade (Petrill, Deater-Deckard, Thompson, Schatschneider, & DeThorne, in press). These twin studies also suggested substantial genetic stability. They yielded mixed results concerning shared environmental influence, as expected given the confidence intervals surrounding twin study estimates of shared environment mentioned above, but on balance the studies suggest that shared environmental influences contribute to stability and that nonshared environment is largely responsible for change. We look forward to future reports from these two studies because they include diverse measures of reading and language-related skills such as phonological awareness, rapid automatized naming, and spelling.

We are aware of no longitudinal studies of learning abilities other than reading and none for learning disabilities. Similar to the studies of reading, longitudinal studies of “*g*” indicate substantial genetic stability in childhood (Bartels, Rietveld, van Baal, & Boomsma, 2002a; Petrill et al., 2004), adulthood (Loehlin, Horn, & Willerman, 1989), and even late in life (Plomin, Pedersen, Lichtenstein, & McClearn, 1994). Also similar to reading, “*g*” shows less shared environmental influence, but to the extent that shared environment can be detected it appears that it is largely stable from age to age. Change from age to age is due to nonshared environment.

Chapter V presents TEDS results that address these two issues of quantitative age differences and qualitative age changes at 7, 9, and 10 years for learning abilities and, for the first time, for learning disabilities.

### HETEROGENEITY AND HOMOGENEITY

The third way in which the present monograph goes beyond the basic nature–nurture question is to investigate genetic and environmental links between learning abilities. For example, to what extent do genes that affect reading ability also affect mathematics? In contrast to univariate genetic analysis that focuses on genetic and environmental contributions to the variance of a single variable, multivariate genetic analysis investigates the covariance between variables and estimates the extent to which genetic and environmental factors that affect one variable also affect other variables. (Chapter II describes multivariate genetic analysis.)

The surprise from the few extant multivariate genetic analyses of learning abilities is that genetic correlations are high, which suggests that the same genes affect different abilities. In a recent review, genetic correlations varied from .67 to 1.0 for reading versus language (five studies), from .47 to .98 for reading versus mathematics (three studies), and from .59 to .98 for language versus mathematics (two studies) (Plomin & Kovas, 2005). The average genetic correlation is about .70, which can be interpreted to mean that when genes are found that are associated with one learning ability such as reading there is about a 70% chance that the genes will also be associated with other learning abilities such as mathematics. There is only one small multivariate genetic study of learning disabilities and it reported a genetic correlation of .53 between reading disability and mathematics disability (Knopik, Alarcón, & DeFries, 1997). If genetic correlations are so high between learning abilities, it makes sense to expect that components within each learning domain (e.g., read words vs. reading nonwords) are also highly correlated genetically, and that is the case. Genetic correlations range between .60 and .90 within each of the domains of language, reading, and mathematics (Plomin & Kovas, 2005). Multivariate genetic research on cognitive abilities such as verbal, spatial, and memory abilities also consistently find genetic correlations greater than .50 and often near 1.0 across diverse cognitive abilities, including basic information processing measures (Deary, Spinath, & Bates, 2006). This genetic overlap across cognitive abilities becomes stronger later in the life span (Petrill, 2002). Phenotypic correlations among diverse tests of cognitive abilities led Charles Spearman in 1904 to call this general factor “*g*” in order to avoid the many connotations of the word *intelligence*. To what extent do genes for “*g*” overlap with genes for specific learning abilities such as reading? A review of a dozen such studies concludes that genetic correlations between learning abilities (mostly reading) and “*g*” are substantial but somewhat lower than the genetic correlations among learning abilities (Plomin & Kovas, 2005), which is consistent with a paper published since this review (Wainwright et al., 2005a, 2005b). This result suggests that most (but not all) genes that affect learning abilities are even more general in that they also affect other sorts of cognitive abilities included in the “*g*” factor.

Multivariate genetic analysis also provides information on shared and nonshared environmental links between abilities. The first multivariate genetic analysis of learning abilities in childhood was subtitled *Genetic Overlap but Environmental Differences* because it found a genetic correlation of .98 between reading and mathematics but a nonshared environmental correlation of .28 (Thompson et al., 1991). Other multivariate genetic analyses tend to be consistent with the conclusion that nonshared environments are specialists (Kovas & Plomin, 2007).

These multivariate genetic results led to the development of a theory called “generalist genes,” which proposes that the same set of genes affects individual differences in diverse learning and cognitive abilities (Plomin & Kovas, 2005). If true, the generalist genes theory would have widespread implications for molecular genetics, cognitive neuroscience, and education (Kovas & Plomin, 2006). However, the theory is based on a fragile foundation of a few small and diverse studies, especially for learning abilities. In particular, larger studies are needed because multivariate genetic analysis is especially demanding in relation to statistical power (Rhee, Hewitt, Corley, & Willcutt, 2005). Chapter VI presents multivariate genetic analyses using the large TEDS sample that investigate genetic and environmental links within each domain of learning abilities (e.g., reading words vs. nonwords), between domains of learning abilities (e.g., reading vs. mathematics), and between learning abilities and “*g*.”

### NATURE AND NURTURE AGAIN

Chapter VII discusses our findings in relation to the three themes of this monograph: the etiological relationship between the normal (learning abilities) and the abnormal (learning disabilities), genetic and environmental contributions to stability and change from 7 to 10 years, and genetic and environmental heterogeneity and homogeneity within and between learning abilities as well as their relationship to general cognitive ability. These three themes go beyond the fundamental nature–nurture question, but in Chapter VII we also return to more general issues related to nature and nurture, including some limitations of our study, findings that surprised us and some puzzles that remain, and implications of this research for theories of education and child development.

## II. METHODS

In this chapter, we describe the sample, measures, and analyses used to investigate the genetic and environmental origins of learning abilities and disabilities in the early school years. We also present descriptive statistics for all of the measures at 7, 9, and 10 years.

### PARTICIPANTS

All analyses reported in this monograph are based on data collected as part of the Twins' Early Development Study (TEDS), a longitudinal study involving a representative sample of all twins born in England and Wales in 1994, 1995, and 1996 (Oliver & Plomin, 2007; Trouton, Spinath, & Plomin, 2002). Families of twins (*n*=25,815) were identified by the Office for National Statistics (ONS) from their children's birth records and contacted when the children were 1 year old. Of all families (*n*=16,810) who responded that they were interested in participating in TEDS, 12,054 families have been involved in TEDS since its inception, at least for one assessment point. Various subsets of this foundation sample were assessed at each age, as described later.

Although cognitive and language data were obtained in TEDS at 2–4 years (e.g., Colledge et al., 2002; Dale et al., 1998; Dale, Dionne, Eley, & Plomin, 2000; Dionne, Dale, Boivin, & Plomin, 2003; Hayiou-Thomas et al., 2006; Kovas et al., 2005; Price, Dale, & Plomin, 2004; Spinath, Ronald, Harlaar, Price, & Plomin, 2003; Spinath, Harlaar, Ronald, & Plomin, 2004; Viding et al., 2003; Viding et al., 2004), the focus of this monograph is on learning abilities assessed at 7, 9, and 10 years. These ages correspond to the early school years during which important changes in academic content occur, reflected in the U.K. National Curriculum (NC) by a second key stage (see Appendices A–C). The NC across all of the key stages is based on an 8-point scale. The differences between the key stages reflect the expectation that children of a certain age should score appropriately on this scale. For example, at the end of key stage 1 most children reach level 2, and at the end of key stage 2 most children reach level 4. These changes are also accompanied by major content and difficulty changes. For example, for English: Speaking, and Listening, at the end of key stage 1 children are expected to reach level 2, which is described as children beginning to show confidence with speaking and listening. By the end of key stage 2 children are expected to have reached level 4 where they are able to talk and listen with confidence (see Appendices A–C that detail the attainment targets for each level).

Before analysis, the following exclusion criteria were applied: specific medical syndromes such as Down syndrome and other chromosomal anomalies, cystic fibrosis, and cerebral palsy; severe hearing loss; autism spectrum disorder; organic brain damage; extreme outliers for birth weight and gestational age; heavy maternal alcohol consumption (>13 units of alcohol per week) during pregnancy; and intensive care after birth. Although the numbers of children excluded varies for different analyses, in general 8% of the sample was excluded on the basis of these criteria.

Table 1 summarizes the sample sizes at each age after exclusions. Although teacher ratings at 7 years were obtained from all three cohorts, funds were available only to include the first two cohorts for the other measures and other ages.

**TABLE 1:** NUMBER OF INDIVIDUALS AT EACH WAVE OF TESTING

	National Curriculum (NC)	Tests		
Age	English Math Science	Reading	Math	“*g*”
7	Cohorts 1–3: *n*=11,333–11,482	Cohorts 1–2: *n*=9,925–9,979 (telephone testing)	—	Cohorts 1–2: *n*=9,940 (telephone testing)
9	Cohorts 1–2: *n*=5,319–5,421	—	—	Cohorts 1–2: *n*=6,259 (booklet)
10	Cohorts 1–2: *n*=5,561–5,690	Cohorts 1–2: *n*=5,808 (web)	Cohorts 1–2: *n*=5,348 (web)	Cohorts 1–2: *n*=5,084 (web)

*Note*.—Cohorts are based on children's dates of birth: Cohort 1: January 1994–August 1995; Cohort 2: September 1995–December 1995; Cohort 3: January 1996–December 1996. *n*, number of individuals in each cohort. The mean age in years (and standard deviation) at the time of testing was 7.1 (.24) at 7 years, 9.0 (.28) at 9 years, and 10.1 (.28) at 10 years.

#### Representativeness

Considering the major burden imposed by the booklets on harried parents of young twins and our lack of pressure on the parents in order to avoid having families drop out of the study, a gratifyingly large number of parents completed the time-consuming booklets, which testifies to the well-known phenomenon of excellent cooperation from parents of young twins. Each year, parents were given the opportunity to indicate by checking a box that they no longer wish to participate in the study; after 10 years, only 1,147 of the 16,810 (6.8%) families have so indicated.

TEDS families are reasonably representative as compared with U.K. census data for families with children. Table 2 indicates that mothers in the total TEDS sample are representative of the United Kingdom population for ethnicity and for the percentage who completed A-level exams, which are taken by students finishing secondary school who plan to go to university. Moreover, mothers who completed all test booklets at each age (third column in Table 2) do not differ from the total TEDS sample (second column) for ethnicity and A-level exams. The percentage of mothers who had no educational qualifications (i.e., in the U.K. system they did not pass the examinations as part of the General Certification in Secondary Education or any higher examinations) was somewhat higher and the percentage of working mothers was somewhat lower in TEDS as compared with all mothers in the United Kingdom.

**TABLE 2:** TEDS REPRESENTATIVENESS

Mother	U.K.	TEDS	TEDS complete data
White (%)	92	92	94
A-levels (%)	32	34	39
School leaver (%)	19	10	7
Employed (%)	49	41	42

*Note*.—U.K., U.K. census data; TEDS, the total TEDS sample; TEDS complete data, subsample for whom all the booklets have been completed.

#### Zygosity

A parent-rated questionnaire was used to assign twin zygosity of same-sex twins when the twins were 18 months old, and again when twins were 3 and 4 years old. (Opposite-sex twins are of course always DZ.) This questionnaire includes items such as whether the twins are “as physically alike as two peas in the pod,” whether they have hair that is similar in color and texture, and whether they have the same eye color. At 18 months of age, zygosity was correctly assigned by parent ratings in 94% of cases as validated against zygosity assigned by identity of polymorphic DNA markers using DNA extracted from cheek swabs (for details see Freeman et al., 2003; Price et al., 2000).

These results validate the use of parental report questionnaire data to assign zygosity even in infancy, and concur with other studies showing that the determination of zygosity in twins based on questionnaires can be done with a high degree of accuracy (for a review, see Rietveld, van Baal, Dolan, & Boomsma, 2000). For the sample used in this monograph, we used zygosity information assessed from DNA when it was available (34% of the total sample). DNA is available for twice as many pairs in anticipation of future molecular genetic studies. However, zygosity tests are costly and were conducted only when the parents requested zygosity testing or when the twins' zygosity was doubtful. For the rest of the sample, zygosity of same-sex twins was based on parental assessments of their twins' physical similarity. As expected, roughly one-third of the twins are MZ, one-third are same-sex DZ, and one-third are opposite-sex DZ.

### OVERVIEW OF MEASURES AND PROCEDURES

At ages 7, 9, and 10, data collection was based on the school year (September–August). Rating scales and questionnaires were sent to teachers in the spring term to ensure that each child had received approximately the same contact time with teachers and to allow teachers to become familiar with the children's achievement and behavior over the academic year. Both members of a twin pair were rated by a single teacher if they were in the same classroom; co-twins were rated by different teachers if they were in different classrooms. The percentages of twins rated by the same teacher were 67% at 7 years, 63% at 9 years, and 58% at 10 years. Informed consent was obtained in writing from parents at each assessment so that they were free to withdraw from that particular part of the project, as well as having the option of withdrawing from the entire study as well. Informed consent was also obtained from teachers.

#### Teacher NC Assessments at 7, 9, and 10 Years

When the twins were 7, 9, and 10 years of age (corresponding to the second, fourth, and fifth years of school in the United Kingdom), their teachers assessed three broad areas of ability: English (including Speaking and Listening, Reading, and Writing), mathematics (including Using and Applying Mathematics, Numbers, and Shapes, Space, and Measures), and science (including Scientific Enquiry, Life Processes, and Physical Processes), which was assessed at 9 and 10 only. These assessments were based on the U.K. NC, the core academic curriculum developed by the Qualifications and Curriculum Authority (QCA), and the National Foundation for Educational Research (NFER) (QCA: http://www.qca.org.uk; NFER: http://www.nfer.ac.uk/index.cfm). This assessment follows from requirements of key stages for attainment in English, Mathematics, and Science. Although U.K. teachers are well familiar with these criteria, we reminded them of these criteria as part of our mailing (see Appendices A–D).

The second year of school (age 7) corresponds to NC key stage 1, and the fourth and fifth school years (ages 9 and 10) correspond to NC key stage 2 (Qualifications and Curriculum Authority, 1999; Qualifications and Curriculum Authority, 2003). For the NC Teacher Assessments, at the end of the school year, teachers summarize students' performance throughout the school year in each of these areas using a 5-point scale (see Appendices A–C for full details of the scales for each subject). This judgment was not made specifically for the present study, but rather forms the continuing assessment of each child that ultimately leads to the final NC Teacher Assessment score submitted to the QCA at the end of the school year to indicate the child's academic achievement during that year. (Other measures such as QCA-administered tests also contribute to children's grades, but we did not have access to these data.) We asked teachers to provide this rating using a similar format. In addition to analyzing the three components within each of the three broad areas of achievement, composite measures were created for each of the three broad areas at each age (English composite, Mathematics composite, and Science composite) by calculating a mean for the three scores. The use of composites to represent each area was supported by the results of factor analyses (computed using one twin from each pair), which showed high first unrotated principal component loadings for all measures at all ages (average variance explained by the first principal component=87%, range=78–93%).

There is growing evidence for the validity of teacher assessments. In TEDS, for example, a general factor for NC ratings at 7 years has been found to correlate .58 with a general factor of telephone-administered tests of verbal and nonverbal cognitive abilities (Spinath, Ronald, Harlaar, Price, & Plomin, 2003). Correlations between NC ratings and test data also support the validity of teacher assessments, as described in Chapter VI.

#### Telephone Testing at 7 Years

At age 7, we assessed the children's reading and general cognitive ability on the telephone. Our telephone adaptation of the tests retained the original test materials, and the administration procedure was closely aligned to the standard face-to-face procedure. Item lists were mailed to families in a sealed envelope before the test sessions. Twins in each pair were tested within the same test session and by the same tester, who was blind to zygosity. Several precautions were taken to prevent cheating. First, it was emphasized to parents that the test items were meant for a range of ages and that no 7-year-old children would be able to perform successfully on all tasks. Second, test stimuli were mailed to families in a sealed envelope before the test sessions with separate instructions that the envelope should not be opened until the time of testing. Third, parents were asked to provide a room that was free from distractions, such as other family members and operating televisions. Finally, the testing procedure provided no opportunity for parental intercession.

Telephone-administered measures have been shown to be efficient and cost-effective alternatives to in-person assessments. Recent reports have demonstrated good reliability and validity of telephone assessments. For example, in a validation study of telephone-administered cognitive measures 52 children as young as 6 years were recruited as part of a larger volunteer family registry at Wesleyan University, U.S.A. (Petrill, Rempell, Oliver, & Plomin, 2002). These children were assessed using the telephone battery and then tested at home using the Stanford-Binet (SB) Intelligence Scale (Thorndike, Hagen, & Sattler, 1986). A general cognitive ability composite from the telephone-administered battery and the SB correlated .62. We have also shown in TEDS that a word recognition test administered by telephone correlated .70 with NC teacher assessments of reading (Dale, Harlaar, & Plomin, 2005).

#### Booklet Testing at 9 Years

Nine-year-old participants received a test booklet containing four cognitive tests that were administered under the supervision of the parent who was guided by an instruction booklet. As with telephone testing, precautions were taken to prevent cheating. Correlations with telephone-administered and web-administered cognitive testing are described in Chapters V.

#### Web-Based Testing at 10 Years

At age 10 children participated in web-based testing. The internet is well suited to children as young as 10, most of whom are competent computer users. Web-based testing can be interactive and enjoyable; ease of understanding the test questions can be facilitated by including voice instructions as well as on-screen text as well as graphics and practice items. Branching rules on some tests allowed for adaptive testing, which increases their engagement while limiting the number of items that need to be answered (Birnbaum, 2004).

The use of web-based assessment facilitates data collection because it allows data from large widely dispersed samples to be collected quickly, cheaply, and reliably. Web-based data collection is less error prone because it does not require human transcription and data entry (Kraut et al., 2004; Naglieri et al., 2004). Another positive aspect of web testing is that the social pressure or embarrassment which might be present in face-to-face testing is reduced (Kraut et al., 2004; Birnbaum, 2004). Moreover, several recent empirical studies have found that web-based findings generalize across presentation formats, and are consistent with findings from traditional methods (e.g., Gosling, Vazire, Srivastava, & John, 2004).

In TEDS, 80% of the families have daily access to the internet (based on a pilot study with 100 randomly selected TEDS' families), which is similar to the results of market surveys of U.K. families with adolescents. Most children without access to the internet at home have access in their schools and local libraries.

In designing our web-based battery, we guarded against potential problems associated with research on the internet. The web page and testing were administered by a secure server in the TEDS office (the TEDS' web page can be accessed at http://www.teds.ac.uk). We used a secure site for data storage; identifying information is kept separately from the data. Safeguards were in place that prevented children from answering the same item more than once. We provided technical support and other advice to parents and children who were advised to call our toll-free telephone number in case of any problems or questions.

Parents supervised the testing by coming online first with a user name and password for the family, examining a demonstration test and completing a consent form. Then parents allowed each twin to complete the test in turn. Parents were urged not to assist the twins with answers and not to allow the twins to see each other's answers. We are confident on the basis of our telephone interactions with many of the parents that parents complied with these requirements, most of whom have participated in the TEDS research program for a decade.

### MEASURES

#### English

##### NC

When children were seven, teachers assessed academic achievement in three areas of English at key stage 1, designed for children aged 5–7 years. The QCA provides teachers with guidelines for assessments that aim to cover diverse aspects of the three areas, Writing, Reading, and Speaking/Listening. (See Appendix A for the 5-point NC criteria given by the QCA and used by teachers to indicate achievement levels in each of the three areas of English.) The same three areas were assessed when the children were 9 and 10 using key stage 2 NC criteria.

##### Tests

When children were 7, the Test of Word Reading Efficiency (TOWRE, Form B; Torgesen, Wagner, & Rashotte, 1999) was administered to children over the telephone. The TOWRE, a standardized measure of fluency and accuracy in word reading skills, includes two subtests, each printed on a single sheet: A list of 85 words, called Sight-word Efficiency (SWE), which assesses the ability to read aloud real words, and a list of 54 non-words, called Phonemic Decoding Efficiency (PDE), which assesses the ability to read aloud pronounceable printed nonwords. The child is given 45 seconds to read as many words as possible. Twins were individually assessed by telephone using test stimuli that had been mailed to families in a sealed package with separate instructions that the package should not be opened until the time of testing. The same tester, who was blind to zygosity, assessed both twins in a pair within the same test session. In addition to looking at each component, a reading composite was also created, as supported by the correlation of .83 between the two subtests.

Although we are not aware of any previous studies that have administered reading tests by telephone, we recently examined reading scores for 54 twin pairs from the 1994 cohort who participated in the 7-year telephone testing and who were also tested by telephone at age 9 on Form A of the TOWRE and on the comprehension subtest of the Neale Analysis of Reading Ability (NARA)-II; (Neale, 1997). For the 108 children, the correlation between TOWRE Form B at 7 years and TOWRE Form A at 9 years was .83. This finding is consistent with previous research demonstrating the longitudinal stability of word level reading skills (Juel, 1988; Torgesen et al., 1999) and can be seen as a lower-limit estimate of reliability. Furthermore, the correlation between the TOWRE composite and the NARA-II comprehension test was .73, consistent with previous research demonstrating the association between word identification and later reading performance (e.g., Juel, 1988; Storch & Whitehurst, 2002). In addition, our results (e.g., standard deviations, twin correlations, heritability estimates) mirror very closely the TOWRE results from a U.S. study in which the TOWRE was administered in the standard format to twins in kindergarten (age 6) and first grade (age 7) (Byrne et al., 2005). Although TOWRE's standardization has been done in the United States, rather than United Kingdom, the focus of this study is not on how the children compared with norms, but rather on variance within the sample.

At age 10, participants completed a web-based adaptation of the reading comprehension subtest of the Peabody Individual Achievement Test (Markwardt, 1997) at home (hereafter referred to as PIAT). The PIAT assesses literal comprehension of sentences. Sentence items were presented visually and with oral instructions given by the computer using digitized speech. The children responded by selecting the picture described by the sentence using the mouse, moving the pointer to the desired location and clicking on it. All the children started with the same items, but an adaptive algorithm modified item order and test discontinuation depending on the performance of the participant. Children could attempt each item only once. The web-based adaptation of the PIAT contained the same practice items, test items, and instructions as the original published test. Credit (automatic score of 1) was given for all items that were skipped due to upward branching. PIAT total scores were derived by summing correct and credited scores. Test–retest reliability of the PIAT across 7 months was .66 in a subsample of the TEDS twins (*n*=55). The PIAT also shows good internal consistency (Cronbach's *α*=.95).

#### Mathematics

##### NC

When children were seven, teachers assessed academic achievement in three areas of mathematics at key stage 1, designed for children aged 5–7 years. The QCA provides teachers with guidelines for assessments that aim to cover diverse aspects of the three domains: *Using and Applying Mathematics*, *Numbers*, and *Shapes, Space, and Measures* (see Appendix B for the 5-point NC criteria given by the QCA and used by teachers to indicate achievement levels in each of the three areas of mathematics). The same three areas were assessed when the children were 9 and 10 using key stage 2 NC criteria (see Appendix B).

##### Tests

We developed a web-based battery that assessed three aspects of mathematics performance (described below) when the children were 10. The items were based on the NFER 5–14 Mathematics Series, which is linked closely to curriculum requirements in the United Kingdom and the English Numeracy Strategy (nferNelson, 1994, 1999, 2001). Such curriculum-based assessment alleviates some of the potential biases associated with other achievement tests (Good & Salvia, 1988). From booklets 6–11 (referring to age of students), a total of 77 target items were chosen. The items were organized by mathematical subtest and level of difficulty. The level of difficulty was based on the NC level and the percentage correct for each item from the NC standardization sample (reported in the Group Record Sheets, nferNelson). A set of adaptive branching rules was developed separately for each of three subtests, so that all the children started with the same items, but then were branched to easier or harder items depending on their performance. The presentation of items was streamed, so that items from the three subtests were mixed to make the test more interesting, but the data recording and branching were done within each subtest. Participants could attempt each item only once.

As with many psychological tests that use branching (e.g., Wechsler Intelligence Scale for Children (WISC-III-UK, Wechsler, 1992)), the general scoring rules were as follows: 1 point was recorded for each correct response, for each unadministered item preceding the child's starting point, and for each item skipped through branching to harder items. After a certain number of failures, a discontinuation rule was applied within each area, and no points were recorded for all items after discontinuation. Thus, for each of the 77 items, a score of 1 or 0 was recorded for each child. For example, for Computation and Knowledge (total number of items=31), all children started at item 10. The following rules were then applied:

- If items 10–12 were all answered incorrectly, the child was branched to item 1, and had to continue with the test attempting all remaining items, or until the discontinuation criterion was met.
- If items 10–12 were all answered correctly, the child received credit for all preceding items (1–9), and was branched to item 24. If items 24–26 were all answered incorrectly, the child was branched back to item 13 and had to continue with the test (skipping all items administered previously), attempting all remaining items, or until the discontinuation criterion was met. If one or two of items 24–26 were answered incorrectly the child received credit for all preceding items (13–23) and then continued with the test, attempting items 27–31, or until the discontinuation criterion was met.
- If items 10–12 were not all answered incorrectly or correctly (i.e., if some but not all were answered correctly), the child received credit for all preceding items (1–9) and then had to continue with the test, attempting at all remaining items or until the discontinuation criterion was met.
- Discontinuation criterion: three incorrect answers in a row (does not apply across branching points).

As with other psychological tests with items of increasing difficulty and using similar rules, this scoring system for our branching approach is meant to mirror the traditional approach in which all children attempt all items, allowing us to calculate total number and proportion of correct responses for each child for each subtest, as well as testing the internal consistency of each subtest. Specific branching and discontinuation rules and the number of skipped (credited) items for each subtest are available from the authors.

The items were drawn from the following three subtests:

*Understanding Number* (27 items) requires an understanding of the numerical and algebraic process to be applied when solving problems (such as understanding that multiplication and division are inverse operations). For example, “Look at the number 6085. Change the order of the figures around to make the biggest number possible.” Another example is: “Type the missing number in the box: 27+27+27+27+27=27 × _.”

*Nonnumerical Processes* (19 items) requires understanding of nonnumerical mathematical processes and concepts such as rotational or reflective symmetry and other spatial operations. The questions do not have any significant numerical content that needs to be considered by the pupils. Three examples follow: “Which is the longest drinking straw? Click on it.”“One of these shapes has corners that are the same. Click on this shape.”“Which card appears the same when turned upside down? Click on it.”

*Computation and Knowledge* (31 items) assesses the ability to perform straightforward computations using well-rehearsed pencil and paper techniques and the ability to recall mathematical facts and terminology. These questions are either algorithmic or rely upon memorizing mathematical facts and terminology. The operation is stated or is relatively unambiguous. Three examples follow. “Type in the answer: 76 – 39.”“All four-sided shapes are called? Click on the answer (squares rectangles parallelograms kites quadrilaterals).”“Type in the answer: 149+785=?.”

A composite score was also created using the mean of the percentage scores of the three tests. This was supported by the high correlations between the three tests; as reported in Chapter VI, the average correlation was .59.

The web-administered measures yielded high Chronbach's *α* coefficients (Understanding Number: *α*=.88; Nonnumerical Processes: *α*=.78; Computation and Knowledge: *α*=.93).

Finally, in terms of validity, we were able to compare children's overall web-based performance in mathematics at 10 years to their overall mathematics performance in the classroom as assessed by their teachers on the national curriculum criteria when the children were 10 years old and we found a correlation of .53 ( *p*<.001, *N*=1,878). Only one twin from each pair was randomly selected for this analysis; a similar correlation of .50 was found for the other half of the sample.

As a direct test of the reliability and validity of the web-based measures, we conducted a test–retest study in which thirty 12-year-old children (members of 15 twin pairs) who had completed the web-based testing were administered the tests in person using the standard 12-year paper and pencil version of the test (nferNelson, 2001). Stratified sampling was used to ensure coverage of the full range of ability. The interval between test and retest was 1–3 months with an average of 2.2 months. The total math score from our web-based tests correlated .92 with the total score from the in-person testing for the total sample of 30 children; generalized estimation equations that take into account the nested covariance structure yielded a correlation of .93. For the three subtests reported in this paper the correlations between the web and the paper and pencil scores were .77, .64, and .81 for Understanding Number, Nonnumerical Processes, and Computation and Knowledge, respectively. These results demonstrate that our web-based testing is both highly reliable and valid, at least at 12 years.

#### Science

##### NC

As for all children in U.K. schools, the twins' scientific performance was assessed throughout the fourth and the fifth years of school (corresponding to age 9 and 10) by their teachers, using criteria and tests of the NC. In the current study, the NC Teacher Assessments at key stage 2 were used, which are familiar to teachers and are designed for children age 8 through their sixth year of primary school at age 11. For key stage 2, the QCA provides teachers with NC material and assessment guidelines for three strands of science which directly map on to areas in science that are taught throughout the NC at this stage: *Scientific Enquiry*, *Life Processes*, and *Physical Processes* (see Appendix C for the 5-point NC criteria given by the QCA and used by teachers to indicate achievement levels in each of the three areas of science).

#### General Cognitive Ability

We assessed general cognitive ability (“*g*”) at 7, 9, and 10 using two verbal tests and two nonverbal tests but with very different procedures at each age (from telephone testing at 7 to parent administration of mailed booklets at 9 and to web-based testing at 10). At each age, we selected tests that were highly loaded on “*g*” and well suited to the particular format of administration.

##### Age 7

Two verbal and two nonverbal cognitive measures designed to yield an index of “*g*” were administered over the telephone using the same procedure as described in the aforementioned section on reading at 7. The verbal measures were the Vocabulary (what does “strenuous” mean?) and Similarities (in what way are milk and water alike?) subtests of the Wechsler Intelligence Scale for Children (WISC-III-UK; Wechsler, 1992). The nonverbal measures were the Picture Completion subtest from the Wechsler Scale, in which a child needs to find a missing part in a picture in 20 seconds, and Conceptual Grouping from the McCarthy Scales of Children's Abilities (MCSA; McCarthy, 1972), which assesses the child's ability to deal logically with objects, to classify, and to generalize. Scores from our telephone adaptations of these standard cognitive tests have been shown to be substantially correlated with both subtest and composite scores from in-person assessments using the Stanford-Binet Intelligence Scale (Thorndike, Hagen, & Sattler, 1986) in 6- to 8-year-old children (Petrill et al., 2002).

##### Age 9

Nine-year-old participants received a test booklet containing two nonverbal and two verbal tests that were administered under the supervision of the parent (guided by an instruction booklet). The verbal tests included two tests adapted from the WISC-III (Wechsler, 1992): Vocabulary (what does “migrate” mean?) and a General Knowledge test (in which direction does the sun set?) adapted from the Information subtest of the multiple choice version of WISC-III (Kaplan, Fein, Kramer, Delis, & Morris, 1999).

The nonverbal tests included a Puzzle test adapted from the Figure Classification subtest of the Cognitive Abilities Test 3 (CAT) (Smith, Fernandes, & Strand, 2001). This test involves inductive reasoning and a minor element of visualization. The child is asked to identify which shape, out of five, continues a series. The second nonverbal test is a Shapes test also adapted from the CAT3 Figure Analogies subtest that assesses inductive and deductive reasoning. The child is asked to identify the one shape, out of five, that relates to another shape in the same way as shown by an example (e.g., a rectangle and a square relate to each other like an oval and what other shape?).

##### Age 10

Participants at age 10 were tested on a web-based adaptation of two verbal tests: WISC-III Multiple Choice Information (General Knowledge) and WISC-III Vocabulary Multiple Choice (Wechsler, 1992). Two nonverbal reasoning tests were also administered as part of the web battery: WISC-III-UK Picture Completion (Wechsler, 1992) and Raven's Standard Progressive Matrices (Raven, Court, & Raven, 1996).

##### “*g*” Composites

In addition to examining each test separately, a composite measure was constructed at each age. A mean standardized score was calculated when data were available for all four subtests. The use of a composite was supported by the results of factor analyses (conducted on one twin from each pair), which showed high principal component loadings for all measures at all ages: the first principal component accounted for 47%, 53%, and 55% of the variance of the four measures at 7, 9, and 10 years, respectively.

### PHENOTYPIC ANALYSES

Although all analyses in this monograph are based on standard scores, in order to provide a general characterization of performance we report unadjusted raw score means and standard deviations for NC measures and test scores in Appendix D. Normative data are available for two of the tests. For the TOWRE administered by telephone at age 7, the mean performance of our sample on both subtests corresponds to a standard score of 105. For the web-administered PIAT Reading Comprehension, the mean performance of our sample corresponds to a standard score of 102. The agreement with norms is remarkable, given the different national context (U.K. vs. U.S.), method of administration, and twinship status of the sample, and provides further assurance of the appropriateness of the measures. Moreover, the six mean NC ratings of the TEDS sample at 7 reported in Appendix D are also very close to national norms (available from http://www.standards.dfes.gov.uk/performance for age 7), in every case deviating by less than .2 *SD*. These national norms are not available for ages 9 and 10 because these ages are not at the end of a key stage.

Analysis of variance (ANOVA) was performed on each variable in order to assess the mean effects of sex and zygosity and their interaction on each variable. All scores were corrected for age at time of testing and standardized using the standardized residuals from a regression on age. Tables 3–8 present means and standard deviations and the results of ANOVAs for all measures. These data are corrected for age at time of assessment and standardized to facilitate comparisons between groups; standardized data corrected for age and sex are used in our genetic analyses for reasons explained later (unstandardized means and standard deviations are included in Appendix D).

**TABLE 3:** ENGLISH NC: MEANS (AND *SD*) AT 7, 9, AND 10 (ADJUSTED FOR AGE), BY ZYGOSITY AND SEX; AND ANOVA RESULTS SHOWING SIGNIFICANCE AND EFFECT SIZE, BY SEX AND ZYGOSITY

	Zygosity		Sex		ANOVA		
Measure at 7	MZ (*n*=4,090–4,133)	DZ (*n*=7,296–7,349)	Female (*n*=5,855–5,908)	Male (*n*=5,531–5,574)	Zygosity	Sex	Zygosity × Sex
Speaking and Listening	−.06 (1.02)	.03 (.99)	.08 (.96)	−.09 (1.03)	*p*<.001 *η*^2^=.002	*p*< .001 *η*^2^=.008	*p*=.037 *η*^2^<.001
Reading	−.06 (1.00)	.03 (1.00)	.10 (.96)	−.11 (1.03)	*p*<.001 *η*^2^=.002	*p*<.001 *η*^2^=.011	*p*=.459 *η*^2^<.001
Writing	−.04 (1.00)	.02 (1.00)	.14 (.95)	−.15 (1.03)	*p*<.001 *η*^2^=.001	*p*<.001 *η*^2^=.019	*p*=.768 *η*^2^<.001
Composite	−.06 (1.01)	.03 (.99)	.12 (.95)	−.13 (1.03)	*p*<.001 *η*^2^=.002	*p*<.001 *η*^2^=.016	*p*=.236 *η*^2^<.001

	Zygosity		Sex		ANOVA		
Measure at 9	MZ (*n*=1,947–1,963)	DZ (*n*=3,429–3,458)	Female (*n*=2,824–2,848)	Male (*n*=2,552–2,573)	Zygosity	Sex	Zygosity × Sex

Speaking and Listening	−.06 (1.00)	.03 (1.00)	.11 (.96)	−.12 (1.03)	*p*<.001 *η*^2^=.002	*p*<.001 *η*^2^=.013	*p*=.350 *η*^2^<.001
Reading	−.03 (1.00)	.02 (1.00)	.11 (.95)	−.12 (1.04)	*p*=.049 *η*^2^=.001	*p*<.001 *η*^2^=.014	*p*=.220 *η*^2^<.001
Writing	−.05 (1.00)	.03 (1.00)	.13 (.96)	−.14 (1.02)	*p*=.003 *η*^2^=.002	*p*<.001 *η*^2^=.018	*p*=.327 *η*^2^ < .001
Composite	−.05 (1.00)	.03 (1.00)	.13 (.95)	−.14 (1.03)	*p*=.001 *η*^2^=.002	*p*<.001 *η*^2^=.019	*p*=.217 *η*^2^< .001

	Zygosity		Sex		ANOVA		
Measure at 10	MZ (*n*=2,006–2,033)	DZ (*n*=3,624–3,657)	Female (*n*=2,957–2,992)	Male (*n*=2,673–2,698)	Zygosity	Sex	Zygosity × Sex

Speaking and Listening	−.07 (1.01)	.04 (.99)	.11 (.95)	−.12 (1.04)	*p*<.001 *η*^2^=.003	*p*<.001 *η*^2^=.012	*p*=.719 *η*^2^<.001
Reading	−.05 (1.00)	.03 (1.00)	.09 (.96)	−.10 (1.03)	*p*=.002 *η*^2^=.002	*p*<.001 *η*^2^=.010	*p*=.519 *η*^2^<.001
Writing	−.02 (1.00)	.01 (1.00)	.14 (.95)	−.16 (1.03)	*p*=.048 *η*^2^=.001	*p*<.001 *η*^2^=.022	*p*=.402 *η*^2^<.001
Composite	−.05 (1.01)	.03 (.99)	.12 (.95)	−.14 (1.04)	*p*=.001 *η*^2^=.002	*p*<.001 *η*^2^=.017	*p*=.486 *η*^2^<.001

**TABLE 4:** ENGLISH TESTS: MEANS (AND *SD*) FOR THE TELEPHONE-BASED READING AT 7 AND WEB-BASED READING AT 10 (ADJUSTED FOR AGE), BY ZYGOSITY AND SEX; AND ANOVA RESULTS SHOWING SIGNIFICANCE AND EFFECT SIZE, BY SEX AND ZYGOSITY

	Zygosity		Sex		ANOVA		
Measure at 7	MZ (*n*=3,582–3,602)	DZ (*n*=6,343–6,377)	Female (*n*=5,104–5,138	Male (*n*=4,821–4,841)	Zygosity	Sex	Zygosity × Sex
Towre: word	−.03 (1.01)	.02 (1.00)	.09 (.97)	−.09 (1.02)	*p*=.005 *η*^2^=.001	*p*< .001 *η*^2^=.009	*p*=.003 *η*^2^=.001
Towre: nonword	−.04 (1.01)	.03 (.99)	−.00 (.98)	.00 (1.02)	*p*=.001 *η*^2^=.001	*p*=.582 *η*^2^<.001	*p*=.019 *η*^2^=.001
Towre: composite	−.04 (1.01)	.02 (1.00)	.05 (.97)	−.05 (1.03)	*p*=.001 *η*^2^=.001	*p*<.001 *η*^2^=.003	*p*=.004 *η*^2^=.001

	Zygosity		Sex		ANOVA		
Measure at 10	MZ (*n*=2,110)	DZ (*n*=3,698)	Female (*n*=3,162)	Male (*n*=2,646)	Zygosity	Sex	Zygosity × Sex

PIAT	−.06 (1.00)	.03 (1.00)	−.01 (.97)	.02 (1.04)	*p*=.001 *η*^2^=.002	*p*=.307 *η*^2^<.001	*p*=.871 *η*^2^<.001

**TABLE 5:** MATHEMATICS NC: MEANS (AND *SD*) AT 7, 9, AND 10 (ADJUSTED FOR AGE), BY ZYGOSITY AND SEX; AND ANOVA RESULTS SHOWING SIGNIFICANCE AND EFFECT SIZE, BY SEX AND ZYGOSITY

	Zygosity		Sex		ANOVA		
Measure at 7	MZ (*n*=4,063–4,118)	DZ (*n*=7,270–7,337)	Female (*n*=5,829–5,894)	Male (*n*=5,504–5,561)	Zygosity	Sex	Zygosity × Sex
Using and Applying	−.05 (1.00)	.03 (1.00)	−.04 (.94)	.04 (1.06)	*p*<.001 *η*^2^=.001	*p*=.003 *η*^2^=.001	*p*=.008 *η*^2^=.001
Numbers and Algebra	−.05 (1.00)	.03 (1.00)	−.04 (.95)	.04 (1.05)	*p*<.001 *η*^2^=.001	*p*=.002 *η*^2^=.001	*p*=.028 *η*^2^<.001
Shapes, Space and Measures	−.06 (1.01)	.03 (.99)	−.01 (.94)	.01 (1.06)	*p*<.001 *η*^2^=.002	*p*=.658 *η*^2^<.001	*p*=.028 *η*^2^<.001
Composite	−.05 (1.00)	.03 (1.00)	−.03 (.94)	.03 (1.06)	*p*<.001 *η*^2^=.002	*p*=.026 *η*^2^<.001	*p*=.012 *η*^2^=.001

	Zygosity		Sex		ANOVA		
Measure at 9	MZ (*n*=1,932–1,946)	DZ (*n*=3,413–3,441)	Female (*n*=2,809–2,832)	Male (*n*=2,539–2,555)	Zygosity	Sex	Zygosity × Sex

Using and Applying	−.06 (.99)	.03 (1.01)	−.04 (.96)	.05 (1.04)	*p*=.002 *η*^2^=.002	*p*=.014 *η*^2^=.001	*p*=.050 *η*^2^=.001
Numbers and Algebra	−.05 (.99)	.03 (1.00)	−.06 (.97)	.07 (1.03)	*p*=.005 *η*^2^=.001	*p*<.001 *η*^2^=.003	*p*=.214 *η*^2^<.001
Shapes, Space and Measures	−.06 (.99)	.03 (1.00)	−.02 (.96)	.03 (1.04)	*p*=.001 *η*^2^=.002	*p*=.207 *η*^2^<.001	*p*=.113 *η*^2^<.001
Composite	−.06 (.99)	.03 (1.00)	−.04 (.96)	.05 (1.04)	*p*=.001 *η*^2^=.002	*p*=.008 *η*^2^=.001	*p*=.094 *η*^2^=.001

	Zygosity		Sex		ANOVA		
Measure at 10	MZ (*n*=1,995–2,021)	DZ (*n*=3,596–3,632)	Female (*n*=2,943–2,972)	Male (*n*=2,648–2,681)	Zygosity	Sex	Zygosity × Sex

Using and Applying	−.04 (1.00)	.02 (1.00)	−.06 (.95)	.06 (1.05)	*p*=.020 *η*^2^=.001	*p*<.001 *η*^2^=.003	*p*=.685 *η*^2^<.001
Numbers and Algebra	−.04 (.99)	.02 (1.01)	−.06 (.95)	.07 (1.05)	*p*=.027 *η*^2^=.001	*p*<.001 *η*^2^=.003	*p*=.556 *η*^2^<.001
Shapes, Space and Measures	−.06 (1.00)	.03 (1.00)	−.04 (.95)	.05 (1.05)	*p*=.001 *η*^2^=.002	*p*=.003 *η*^2^=.002	*p*=.770 *η*^2^<.001
Composite	−.05 (1.00)	.03 (1.00)	−.06 (.95)	.06 (1.05)	*p*=.006 *η*^2^=.001	*p*<.001 *η*^2^=.003	*p*=.567 *η*^2^<.001

**TABLE 6:** MATHEMATICS TESTS: MEANS (AND *SD*) FOR THE WEB-BASED MATH AT 10 (ADJUSTED FOR AGE), BY ZYGOSITY AND SEX; AND ANOVA RESULTS SHOWING SIGNIFICANCE AND EFFECT SIZE, BY SEX AND ZYGOSITY

	Zygosity		Sex		ANOVA		
Measure	MZ (*n*=1,941)	DZ (*n*=3,407)	Female (*n*=2,935)	Male (*n*=2,413)	Zygosity	Sex	Zygosity × Sex
Understanding Number	−.03 (1.00)	.02 (1.00)	−.08 (1.00)	.09 (.99)	*p*=.226 *η*^2^<.001	*p*<.001 *η*^2^=.007	*p*=.723 *η*^2^<.001
Nonnumerical Processes	−.04 (1.03)	.02 (.98)	−.05 (.99)	.06 (1.02)	*p*=.079 *η*^2^=.001	*p*<.001 *η*^2^=.002	*p*=.840 *η*^2^<.001
Computation and Knowledge	−.02 (1.00)	.01 (1.00)	−.05 (1.01)	.06 (.98)	*p*=.339 *η*^2^<.001	*p*<.001 *η*^2^=.003	*p*=.471 *η*^2^<.001
Math composite	−.03 (1.01)	.02 (1.00)	−.07 (1.00)	.08 (.99)	*p*=.158 *η*^2^<.001	*p*<001 *η*^2^=.005	*p*=.587 *η*^2^<.001

**TABLE 7:** SCIENCE NC: MEANS (AND *SD*) AT 9 AND 10 (ADJUSTED FOR AGE), BY ZYGOSITY AND SEX; AND ANOVA RESULTS SHOWING SIGNIFICANCE AND EFFECT SIZE, BY SEX AND ZYGOSITY

	Zygosity		Sex		ANOVA		
Measure at 9	MZ (*n*=1,922–1,949)	DZ (*n*=3,397–3,445)	Female (*n*=2,793–2,834)	Male (*n*=2,526–2,560)	Zygosity	Sex	Zygosity × Sex
Scientific Enquiry	−.05 (.99)	.03 (1.00)	−.03 (.96)	.03 (1.04)	*p*=.006 *η*^2^=.001	*p*=.177 *η*^2^<.001	*p*=.019 *η*^2^=.001
Life Processes	−.05 (1.00)	.03 (1.00)	−.01 (.94)	.01(1.06)	*p*=.002 *η*^2^=.002	*p*=.890 *η*^2^<.001	*p*=.098 *η*^2^=.001
Physical Processes	−.03 (.98)	.02 (1.01)	−.03 (.95)	.03 (1.05)	*p*=.064 *η*^2^=.001	*p*=.148 *η*^2^<.001	*p*=.022 *η*^2^=.001
Composite	−.05 (.99)	.03 (1.01)	−.02 (.95)	.02 (1.05)	*p*=.010 *η*^2^=.001	*p*=.340 *η*^2^<.001	*p*=.025 *η*^2^=.001

	Zygosity		Sex		ANOVA		
Measure at 10	MZ (*n*=1,987–2,018)	DZ (*n*=3,574–3,639)	Female (*n*=2,921–2,974)	Male (*n*=2,640–2,683)	Zygosity	Sex	Zygosity × Sex

Scientific Enquiry	−.05 (1.01)	.03 (.99)	−.01 (.95)	.01 (1.05)	*p*=.003 *η*^2^=.002	*p*=.408 *η*^2^<.001	*p*=.958 *η*^2^<.001
Life Processes	−.04 (.99)	.02 (1.00)	−.00 (.96)	.00 (1.04)	*p*=.017 *η*^2^=.001	*p*=.924 *η*^2^<.001	*p*=.919 *η*^2^<.001
Physical Processes	−.04 (1.00)	.02 (1.00)	−.02 (.96)	.02 (1.05)	*p*=.041 *η*^2^=.001	*p*=.148 *η*^2^<.001	*p*=.633 *η*^2^<.001
Composite	−.05 (1.00)	.03 (1.00)	−.01 (.96)	.01 (1.05)	*p*=.008 *η*^2^=.001	*p*=.505 *η*^2^<.001	*p*=.916 *η*^2^<.001

**TABLE 8:** GENERAL COGNITIVE ABILITY (“*g*”): MEANS (AND *SD*) FOR “*g*” AT 7, 9, AND 10 (ADJUSTED FOR AGE), BY ZYGOSITY AND SEX; AND ANOVA RESULTS SHOWING SIGNIFICANCE AND EFFECT SIZE, BY SEX AND ZYGOSITY

	Zygosity		Sex		ANOVA		
	MZ	DZ	Female	Male	Zygosity	Sex	Zygosity × Sex
(“*g*”) at 7 Telephone assessment	−.06 (.99) *n*=3,590	.03 (1.00) *n*=6,350	.00 (.98) *n*=5,122	−.00 (1.02) *n*=4,818	*p*<.001 *η*^2^=.002	*p*=.527 *η*^2^<.001	*p*=.578 *η*^2^<.001
(“*g*”) at 9 Booklet assessment	−.05 (.98) *n*=2,320	.03 (1.01) *n*=3,939	−.02 (.99) *n*=3,348	.02 (1.01) *n*=2,911	*p*=.004 *η*^2^=.001	*p*=.176 *η*^2^<.001	*p*=.291 *η*^2^<.001
(“*g*”) at 10 Web assessment	−.05 (.99) *n*=1,850	.03 (1.00) *n*=3,234	−.06 (.98) *n*=2,804	.08 (1.02) *n*=2,280	*p*=.020 *η*^2^=.001	*p*<.001 *η*^2^=.004	*p*=.645 *η*^2^<.001

It can be seen from Tables 3–8 that sex and zygosity as well as interactions between them were not important factors in explaining variance in any of the measures. Phenotypic correlations among the measures are presented in Chapter VI.

In our genetic analyses (described in the following section) the scores were corrected for age so that age does not contribute to twin resemblance, which is standard in analyses of twin data (McGue & Bouchard, Jr., 1984). The results could be affected even by small differences in age at the time of testing at this important stage of development, which would inflate estimates of shared environment because members of a twin pair are of exactly the same age. For the analyses of individual differences the scores were also corrected for sex differences. This was not done for the extremes analyses in order not to affect the representativeness of groups at low ability cut-offs. For the individual differences analyses (but not the extremes analyses), in order to avoid the possibility that our results were affected by very extreme scores, all pairs in which one or both twins scored 3 or more standard deviations below or above the mean were excluded from each category.

### GENETIC ANALYSES

The twin method, one of the major tools of quantitative genetic research, addresses the origins of individual differences by estimating the proportion of variance that can be attributed to genetic, shared environment, and nonshared environment factors (Plomin et al., in press). In the case of complex traits that are likely to be influenced by multiple factors, the genetic component of variance refers to the influence of alleles at all gene loci that affect the trait. The similarity between twins for any particular trait can be due wholly or in part to these shared genetic effects. Twin similarity may also be due wholly or in part to shared environment, which refers to environmental influences that vary in the population but are experienced similarly by members of pairs of twins. For example, pairs of twins experience similar conditions during gestation, have the same socio-economic status, live in the same family, and usually go to the same school. These factors could reasonably be expected to increase similarity between co-twins. Nonshared environment refers to any aspect of environmental influence that is experienced differently by the two twins and contributes to phenotypic differences between them, including measurement error. Such influences involve aspects of experience that are specific to an individual, such as traumas and diseases, idiosyncratic experiences, different peers, differential treatment by the parents and teachers, and, importantly, different perceptions of such experiences, even if the events appear to be ostensibly the same for the two children.

Genetic influence can be estimated by comparing intraclass correlations for identical (monozygotic, MZ) twins, who are genetically identical, and fraternal (dizygotic, DZ) twins, whose genetic relatedness is on average .50. The phenotypic variance of a trait can be attributed to genetic variance to the extent that the MZ twin correlation exceeds the DZ twin correlation. Specifically, heritability, which is the proportion of phenotypic variance attributed to genetic variance, can be estimated as twice the difference between the MZ and DZ twin correlations. The relatedness for shared (common) environmental influences is assumed to be 1.0 for both MZ and DZ twin pairs who grow up in the same family because they experience equally similar prenatal and postnatal environments. Shared environmental influences are evidenced to the extent that the DZ twins' correlation is more than half of the MZ correlation. Limitations of the twin method can be found elsewhere (e.g., Plomin, DeFries, McClearn, & McGuffin, 2001). Twin correlations for all of the measures at all of the ages are presented in Chapter III.

#### Model Fitting

**FIGURE 1:**
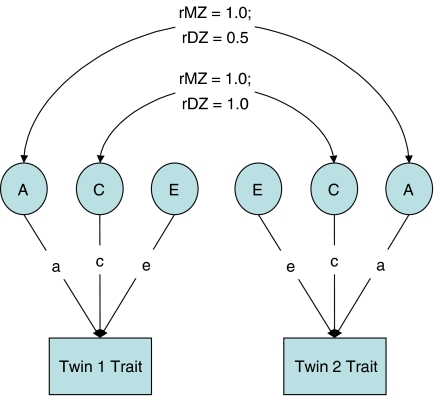
—The basic twin model. A, additive genetic influence; C, shared environment; E, nonshared environment; paths a, c, and e, effects of A, C, and E on a trait; rMZ, monozygotic genetic or shared environmental correlation; rDZ, dizygotic generic or shared environmental correlation.

Structural equation model fitting is a comprehensive way of estimating variance components of a given trait (or, as explained below, of the covariance between traits) based on the principles described above. The fundamental quantitative genetic model is the so-called ACE model. It apportions the phenotypic variance into genetic (A), shared environmental (C), and nonshared environmental (E) components, assuming no effects of nonadditive genetics or nonrandom mating. Figure 1 illustrates the basic logic of this method. The path coefficients of latent variables A (genetic), C (shared environmental), and E (nonshared environmental, including error of measurement) are represented by the lowercase letters a, c, and e, respectively. Genetic relatedness is 1.0 for MZ twins and .5 for DZ twins. Shared environmental relatedness is assumed to be 1.0 for both MZ and DZ twins. The ACE parameters and their confidence intervals can be estimated by fitting the models to variance/covariance matrices using the model-fitting program Mx (Neale, 1997).

ACE model-fitting results of individual differences for the entire sample for all measures at all ages are presented in Chapter III.

#### Sex-Limitation Models

As summarized in Table 9, there are three possibilities with respect to the causes of individual differences in boys and girls, regardless of mean differences between the sexes (Neale & Maes, 2003). The first possibility is that different genetic and environmental factors are responsible for individual differences in mathematics for boys and girls—these are called *qualitative* differences. Such sex-specific effects are not limited to genes on the X chromosome but can also involve genes on the autosomal chromosomes that affect boys and girls differently, for example, because the genes interact with sex hormones. The second possibility, not mutually exclusive with the first, is that the same etiological influences affect individual differences in boys and girls, but that they do so to a different extent—these are known as *quantitative* differences. The third possibility is that there are no differences in the etiology of individual differences for boys and girls; the same genes and environments operate to the same extent in both sexes, even if there are mean differences between boys and girls. That is, mean reading scores are lower for boys than girls, but the factors that make one boy different from another can be the same as those that make one girl different from another girl. It should be noted that quantitative genetics with its focus on individual differences has little to say about the origins of mean differences between boys and girls. Indeed, we frequently find no quantitative or qualitative differences in the etiology of individual differences for boys and girls despite large mean differences (Viding et al., 2004).

**TABLE 9:** THREE POSSIBILITIES WITH RESPECT TO THE CAUSES OF INDIVIDUAL DIFFERENCES IN BOYS AND GIRLS, REGARDLESS OF MEAN DIFFERENCES BETWEEN THE SEXES

Sex Differences in Etiology of Individual Differences	Explanation	Possible Contributing Factors
*Qualitative* differences	Different genetic and environmental factors are responsible for individual differences for boys and girls.	Genes on the sex chromosomes. Genes on the autosomal chromosomes affect boys and girls differently, for example, because the genes interact with sex hormones. Teachers treat boys and girls differently in terms of their expectations or requests for help.
*Quantitative* differences	The same etiological influences affect individual differences in boys and girls, but that they do so to a different extent.	As above, but the differences are in quantity of effects.
No differences in etiology	The same genes and environments operate to the same extent in both sexes.	Boys as a group may exhibit a mean disadvantage, but the factors that make one boy different from another are the same as those that make one girl different from another girl.

**FIGURE 2:**
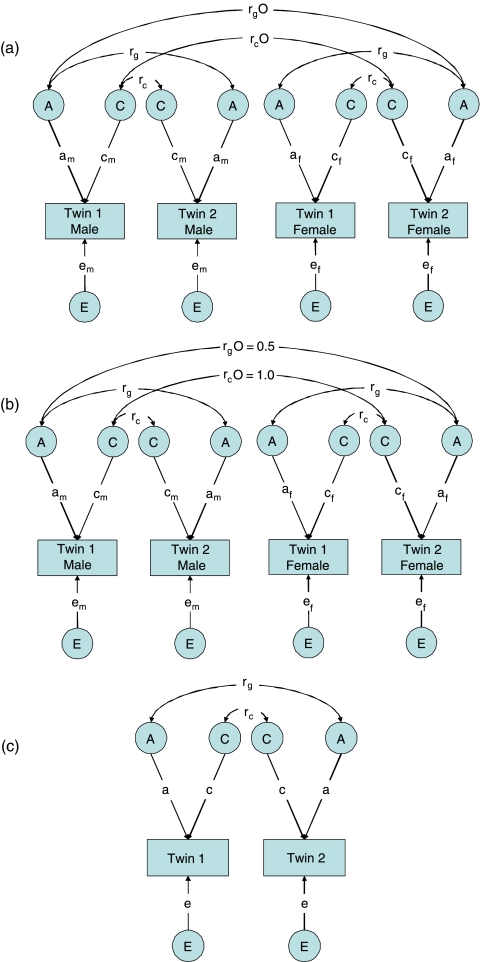
—Full sex-limitation model (a) and nested models (b and c) *Note.—*A, additive genetic influence; C, shared environment influence; E, nonshared environment influence; Paths a, c, and e, effects of A, C, and E on a trait with subscript “m” for males and “f” for females; *r*_*g*_, genetic relatedness between same-sex twins which is fixed at 1.0 for MZ twins and .5 for DZ twins; *r*_*c*_, shared environment relatedness between same-sex twins which is fixed at 1.0 for MZ and DZ twins; *r*_*g*_O, genetic relatedness between opposite-sex twins; *r*_*c*_O, shared environment relatedness between opposite-sex twins. Opposite-sex twins are represented as twin one male and twin two female, and are linked by *r*_*g*_O and *r*_*c*_O. (a) shows the full sex limitation model that estimates seven parameters: *a*_m_, *c*_m_, *e*_m_, *a*_f_, *c*_f_, *e*_f_, and *r*_*g*_O or *r*_*c*_O. This model allows qualitative sex differences in that the genetic and shared environmental correlations (*r*_*g*_O and *r*_*c*_O) between opposite-sex twins are allowed to be <.5 and 1.0, respectively. The model also allows quantitative sex differences in that the ACE parameters for males and females (*a*_m_, *c*_m_, *e*_m_, *a*_f_, *c*_f_, *e*_f_) can differ. Variances differences between the sexes are also allowed (not shown in the path diagram). (b) shows the common effects model, which is nested in the full sex limitation model and tests for qualitative sex differences by constraining the genetic and shared environmental correlations between opposite-sex twins (*r*_G_O and *r*_C_O) to be .5 and 1.0, respectively (i.e., the same as same-sex DZ twins). This model estimates six parameters: *a*_m_, *c*_m_, *e*_m_, *a*_f_, *c*_f_, *e*_f_, and allows variance differences between the sexes. (c) shows the scalar and null models of the sex-limitation design which reduces to the basic twin model and thus, in comparison with the other models, tests for both qualitative and quantitative sex differences. This model estimates three parameters: *a*, *c*, and *e* for the sexes combined. In the scalar model, variance differences between the sexes are allowed. In the null model the variances are equated across the sexes (not shown in the path diagram).

These three possibilities (qualitative differences, quantitative differences, and no differences) can be assessed using sex-limitation structural equation modeling. Each possibility is associated with a set of parameters in the sex-limitation models (see Figure 2). Qualitative differences are evidenced in the genetic relatedness (*r*_*g*_) between DZ opposite-sex twins. In DZ same-sex pairs, the assumption is that on average the twins share 50% of their varying DNA, and the coefficient of genetic relatedness is therefore .5. If there are qualitative differences in etiology between boys and girls (different genetic and environmental factors), the genetic relatedness in DZ opposite-sex twins will be less than .5. If there are quantitative differences (the same factors, but exerting different magnitudes of effect) rather than qualitative differences, the genetic relatedness for DZ opposite-sex pairs will still be .5, but the parameter estimates for the A, C, and E components will be significantly different for male–male pairs and female–female pairs. If there are no qualitative or quantitative differences between boys and girls, the genetic relatedness of DZ opposite-sex (DZos) pairs will be .5 and the A, C, and E estimates for male–male and female–female pairs will be the same. However, the phenotypic variance might nonetheless differ for the two sexes because mean differences are often associated with variance differences (i.e., higher means have higher variances).

Using the model-fitting program *Mx* (Neale, 1997) for each composite measure, we first tested the full model which allows all parameters to vary: *r*_*g*_ in the DZ opposite-sex pairs, A, C, and E estimates, and variance estimates (see Figure 2a). This was fit to variance/covariance matrices derived from the data. A series of nested models was then tested. The first nested model (Figure 2b) is called the common effects sex-limitation model that tests for qualitative sex differences by fixing *r*_g_ to .5 in the DZos, but allows different A, C, E, and variance estimates. The second nested model (Figure 2c) is called a scalar effects sex-limitation model that tests for quantitative sex differences by constraining A, C, and E parameters to be the same in boys and girls as well as constraining *r*_*g*_ to .5 in the DZos; however, it allows differences in phenotypic variance between males and females. The third and final nested model, called the null model (also Figure 2c), tests for variance differences between boys and girls by constraining all the parameters to be equal for males and females. For each model, the ACE parameters and their confidence intervals were estimated. The overall fit of each model was evaluated using the root mean square error of approximation (RMSEA), with lower values representing better fitting models. Results of sex-limitation model fitting are presented in Chapter III.

#### Teacher Heterogeneity Model

In order to test whether being in the same classroom and having the same teacher affected the results of our analyses, we analyzed each of the composite scores separately for the two groups (same vs. different teacher for the two twins in the family). After examining the pattern of twin correlations for the two groups, we performed model-fitting analyses to test whether the differences in estimates for the two groups were statistically significant. The model used for this analysis was similar to that of the sex-limitation models used to test for quantitative sex differences. The full model allowed A, C, and E parameters to vary between the groups. The null model equated the A, C, and E parameters for the two groups. The results from the teacher heterogeneity model are also described in Chapter III. Note that in the case of teacher ratings, being in the same classroom includes the effects of a shared teaching experience and a shared rater, whereas for the test scores, being in the same classroom reflects a shared teaching experience only.

#### Extremes Analyses

The previous model-fitting sections focused on the analysis of individual differences for the entire sample; that is, ability rather than disability. An important feature of TEDS is that its large community sample makes it possible to study disability in the context of ability by selecting children at the low end of the normal distribution. In Chapter IV, we present results for all measures at 7, 9, and 10 years for children in the lowest 15% of the distribution.

For each of the measures, we defined probands as 5% and 15% of the whole sample, identifying statistically low performance on that measure. As results for both cut-offs were generally similar, we only present the results from the 15% cut-off analyses, which provided greater power. Probandwise concordances (the ratio of the number of probands in concordant pairs to the total number of probands) were calculated separately for each measure and each of the five sex-by-zygosity groups. Probandwise concordances represent the risk that a co-twin of a proband is affected. Greater MZ than DZ concordances suggest genetic influence, but unlike twin correlations, twin concordances cannot be used to estimate genetic and environmental parameters because they do not in themselves include information about the population incidence.

**FIGURE 3:**
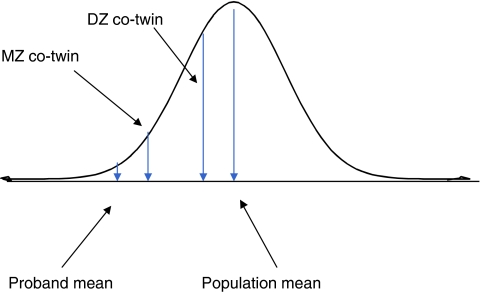
—DF extremes analysis assesses twin similarity as the extent to which the mean standardized quantitative trait score of co-twins is as low as the mean standardized score of selected extreme or diagnosed probands. This measure of twin similarity is called a *group* twin correlation (or *transformed co-twin mean*) in DF extremes analysis because it focuses on the mean quantitative trait score of co-twins rather than individual differences.

DF extremes analysis assesses genetic links between disability and ability by bringing together dichotomous diagnoses of disability and quantitative traits of ability. Rather than assessing twin similarity in terms of individual differences on a quantitative trait of ability or in terms of concordance for a diagnostic cut-off, DF extremes analysis assesses twin similarity as the extent to which the mean standardized quantitative trait score of co-twins of selected extreme or diagnosed probands is below the population mean and approaches the mean standardized score of those probands (see Plomin & Kovas, 2005 for detailed explanation of DF extremes analysis and for discussion of alternative methods). This measure of twin similarity is called a *group* twin correlation (or *transformed co-twin mean*) in DF extremes analysis because it focuses on the mean quantitative trait score of co-twins rather than individual differences. Genetic influence is implied if group twin correlations are greater for MZ than for DZ twins, that is, if the mean standardized score of the co-twins is lower for MZ pairs than for DZ pairs. Doubling the difference between MZ and DZ group twin correlations estimates the genetic contribution to the average phenotypic difference between the probands and the population. The ratio between this genetic estimate and the phenotypic difference between the probands and the population is called *group heritability*. It should be noted that group heritability does not refer to individual differences among the probands–the question is not why one proband is slightly more disabled than another but rather why the probands as a group have lower scores than the rest of the population. Figure 3 illustrates the basic logic of the DF analysis.

Although DF extremes group heritability can be estimated by doubling the difference in MZ and DZ group twin correlations (Plomin, 1991), DF extremes analysis is more properly conducted using a regression model (DeFries & Fulker, 1988). The DF extremes model fits standardized scores for MZ and DZ twins to the regression equation, *C*=*B*_1_*P*+*B*_2_*R*+*A*, where *C* is the predicted score for the co-twin, *P* is the proband score, *R* is the coefficient of genetic relatedness (1.0 for MZ twins and .5 for DZ twins), and *A* is the regression constant. *B*_1_ is the partial regression of the co-twin score on the proband, an index of average MZ and DZ twin resemblance independent of *B*_2_. The focus of DF extremes analysis is on *B*_2_. *B*_2_ is the partial regression of the co-twin score on *R* independent of *B*_1_. It is equivalent to twice the difference between the means for MZ and DZ co-twins adjusted for differences between MZ and DZ probands. In other words, *B*_2_ is the genetic contribution to the phenotypic mean difference between the probands and the population. Group heritability is estimated by dividing *B*_2_ by the difference between the means for probands and the population.

Finding group heritability implies that, first, disability and ability are both heritable, and second, that there are genetic links between the disability and normal variation in the ability. That is, group heritability itself, not the comparison between group heritability and the other estimates of heritability, indicates genetic links between disability and ability. If a measure of extremes (or a diagnosis) were not linked genetically to a quantitative trait, group heritability would be zero. For example, this situation could occur if a severe form of learning disability is due to a single-gene disorder that contributes little to normal variation in learning ability. However, most researchers now believe that common disorders such as learning disabilities are caused by common genetic variants—the common disease/common variant hypothesis (Collins, Euyer, & Chakravarti, 1997)—rather than by a concatenation of rare single-gene disorders. To the extent that the same genes contribute to learning disability and normal variation in learning ability, group heritability will be observed, although the magnitude of group heritability depends on the individual heritability for normal variation and the heritability of disability gleaned from concordances for disability.

The results of these DF extremes analyses are the topic of Chapter IV.

#### Longitudinal Analyses

Cross-sectional designs can be used to compare genetic and environmental estimates across age but are weakened by the use of different samples at each age. One strength of a longitudinal design is that the same sample is studied at each age. However, the most important benefit of a longitudinal design is that analyses of age-to-age change and continuity are possible, as in the previous example of longitudinal DF extremes analysis. Prospective and retrospective longitudinal analyses can be performed using the multivariate twin methodology described in the following section. Longitudinal analyses are described in Chapter V. In Chapter V, we also present, for the first time, an extension of DF extremes analysis to a trait assessed at two measurement occasions, following the approach described in the following section. For longitudinal DF extremes analysis, we selected probands on the basis of reading scores at 7 years and analyzed their co-twins quantitative reading scores, not at 7 years, but at 10 years.

#### Multivariate Analyses

The principles of the twin method can be extended to determine the etiology of the covariance between different traits, which is called multivariate genetic analysis. As mentioned in the previous section, longitudinal analysis is a special case of multivariate analysis in that it focuses on the etiology of the covariance between the same trait at different ages. In contrast to univariate quantitative genetic analysis that decomposes the variance of a single trait into genetic and environmental sources of variance, multivariate genetic analysis decomposes the covariance between traits into genetic and environmental sources of covariance (Martin & Eaves, 1977). In other words, multivariate genetic analysis assesses genetic and environmental factors responsible for the phenotypic correlation between two traits. For example, if the same genes affect different traits (called pleiotropy), a genetic correlation will be observed between the traits.

**FIGURE 4:**
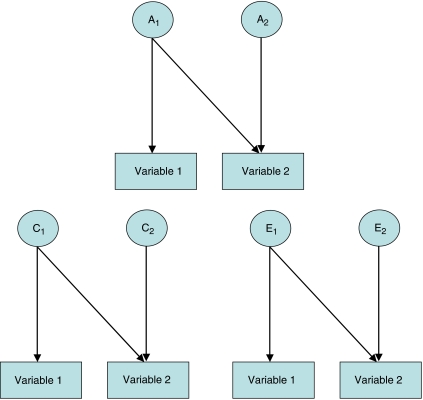
—A typical decomposition (Cholesky decomposition) model that tests for common and independent genetic and environmental effects on variance in two different traits. The same logic applies to the longitudinal analyses, where variable 1 and 2 are replaced with the same variable assessed at ages 1 and 2.

For twin studies, multivariate genetic analysis is based on cross-trait twin correlations for two or more traits. That is, rather than comparing one twin's score on variable *X* with the co-twin's score on the same variable *X*, one twin's *X* is correlated with the co-twin's *Y*. The phenotypic covariance between two traits is attributed wholly or in part to their genetic overlap to the extent that the MZ cross-trait twin correlation exceeds the DZ cross-trait twin correlation. Shared environmental influences are indicated to the extent that DZ twins' correlation is more than half of the MZ correlation. As with the univariate analyses, structural equation modeling, based on the same principles, is used as a more comprehensive way of estimating the proportion of covariance. Figure 4 illustrates a typical model (called Cholesky decomposition) that tests for common and independent genetic and environmental effects on variance in two different traits. The Cholesky procedure is similar to hierarchical regression analyses in nongenetic studies, where the independent contribution of a predictor variable is assessed after accounting for its shared variance with other predictor variables. In the bivariate case, the first factor assesses genetic and shared and nonshared environmental influences on trait 1, some of which also influence trait 2. The second factor estimates genetic and shared and nonshared environmental influences unique to trait 2. The same logic applies to more than two factors.

Another important statistic that can be derived from Cholesky analyses is bivariate heritability. This statistic indexes the extent to which the phenotypic correlation between *X* and *Y* is mediated genetically. That is, univariate heritability is the extent to which the variance of a trait can be explained by genetic variance; bivariate heritability is the extent to which the covariance between two traits (or the same trait at two ages) can be explained by genetic covariance. Bivariate heritability is the genetic correlation (see the next paragraph) weighted by the product of the square roots of the heritabilities of *X* and *Y* and divided by the phenotypic correlation between the two traits (Plomin & DeFries, 1979). The rest of the phenotypic correlation is explained by bivariate shared environment and bivariate nonshared environment.

**FIGURE 5:**
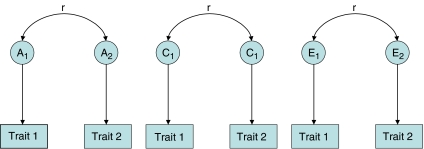
—Correlated factors model. Genetic correlations (*r*_A1A2_) index the extent to which individual differences in the two measures reflect the same genetic influences. Shared and nonshared environmental correlations index the extant to which the same environmental influences affect the two traits.

In addition, the paths from the model can be transformed to obtain the estimates of genetic, shared, and non-shared environmental correlations between each pair of factors. Genetic correlations index the extent to which genetic influences on one measure correlate with genetic influences on a second measure. In other words, genetic correlations indicate the extent to which individual differences in the two measures reflect the same genetic influences. This correlated factors model is illustrated in Figure 5—it is merely an algebraic transformation of the Cholesky model shown in Figure 4. The point is that there are two important statistics: bivariate heritability which is the genetic contribution to the phenotypic correlation between traits, and the genetic correlation which is the extent to which genetic effects on one trait are correlated with genetic effects on another trait. Multivariate genetic analyses are the topic of Chapter VI; Chapter V presents longitudinal genetic analyses based on similar models.

It is also possible to extend DF extremes analysis to address multivariate issues (Light & DeFries, 1995; Plomin & Kovas, 2005), analyzing two traits at the same measurement occasion, or the same trait at two measurement occasions. In Chapter VI, probands were selected on the basis of being in the lowest 15% of web-based reading and mathematics scores at 10 years and analyzed in comparison to their co-twin's reading and mathematics scores. Group heritability indicates the extent to which genetic factors account for the mean difference between probands selected on reading and the population on mathematics. In other words, group heritability in a multivariate extremes analysis indicates the extent to which genetic effects mediate the phenotypic covariance between reading disability and mathematics ability. The group genetic correlation indicates the extent to which the same genetic effects operate on reading disability and mathematics ability. Analysis in both directions is required to estimate a DF extremes genetic correlation—that is, probands were also selected from the lowest 15% of mathematics performance and analyzed with their co-twin's quantitative trait scores on reading. The group genetic correlation can be calculated using the following formula: 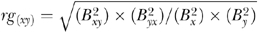
where 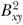 is the group heritability from reading (*x*) to mathematics ( *y*), 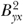 is the group heritability from mathematics to reading, 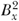 is the group heritability of reading, and 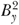 is the group heritability of mathematics (see Knopik, Alarcón, & DeFries, 1997 for details).

#### What Follows

Although this methods chapter is necessarily dense, especially for readers first exposed to these standard quantitative genetic analyses, we hope that applications of these methods and interpretations of the results of these analyses in the following chapters will clarify the concepts. The following chapter presents univariate analyses of individual differences of learning abilities for the total TEDS sample for all measures at 7, 9, and 10 years. Chapter IV focuses on extremes analyses of learning disabilities. Chapter V considers longitudinal analyses of composite measures from 7 to 10 years. Chapter VI addresses multivariate analyses between composite measures at all three ages. Chapter VII summarizes the results in relation to our three themes of the relationship between normal and abnormal, longitudinal analyses of change and continuity, and multivariate analyses of heterogeneity and homogeneity, and also considers limitations and implications of the research.

### III. NATURE AND NURTURE

We begin with estimates of genetic and environmental influence on individual differences in learning abilities in the early school years, with subsequent chapters going beyond these rudimentary issues of nature and nurture to address three issues: the relationship between the normal and abnormal, longitudinal analyses of stability and change, and multivariate analyses of covariance within and between domains. After presenting an overview of the results based on the entire sample, we compare results for boys and girls and for children assessed by the same versus different teachers.

The simple cross-twin correlations tell most of the story of genetic and environmental influence. Table 10 lists cross-twin correlations at 7, 9, and 10 years for U.K. National Curriculum (NC) teacher ratings for English, Mathematics, and Science composite scores and for three components within each domain. These results for teacher ratings are followed by results for test data for reading (TOWRE at 7 years, PIAT at 10 years), Mathematics at 10, and “*g*” at 7, 9, and 10 years. Table 11 presents the model-fitting results for 43 separate analyses; the RMSEA values indicate that the full model with A, C, and E parameters fit the data well. We examine the results in detail below.

**TABLE 10:** INTRACLASS CORRELATIONS BY SEX AND ZYGOSITY

Measure	MZ	DZall	DZss	DZos	MZM	MZF	DZM	DZF
*1.NC measures*								
NC measures at 7 years								
English: speaking and listening	.80	.49	.53	.45	.80	.79	.49	.56
English: reading	.75	.41	.45	.38	.74	.76	.45	.45
English: writing	.67	.36	.38	.34	.68	.66	.36	.39
English: composite	.82	.50	.52	.47	.83	.81	.51	.53
Mathematics: using and applying	.71	.40	.45	.35	.73	.70	.43	.47
Mathematics: numbers and algebra	.70	.39	.41	.35	.72	.68	.38	.45
Mathematics: shapes, space and measures	.74	.43	.46	.39	.76	.73	.44	.48
Mathematics: composite	.78	.44	.47	.40	.78	.77	.46	.49
NC measures at 9 years								
English: speaking and listening	.68	.43	.42	.45	.68	.69	.37	.46
English: reading	.75	.42	.42	.43	.73	.77	.43	.42
English: writing	.72	.37	.38	.35	.72	.72	.36	.41
English: composite	.78	.46	.45	.46	.79	.78	.43	.46
Mathematics: using and applying	.73	.37	.41	.34	.74	.72	.40	.42
Mathematics: numbers and algebra	.71	.38	.40	.35	.68	.74	.38	.41
Mathematics: shapes, space and measures	.72	.41	.43	.38	.70	.75	.40	.46
Mathematics: composite	.76	.41	.44	.38	.75	.78	.43	.44
Science: scientific enquiry	.71	.41	.43	.40	.69	.72	.38	.48
Science: life processes	.74	.41	.40	.41	.76	.71	.39	.42
Science: physical processes	.72	.41	.44	.38	.75	.70	.43	.44
Science: composite	.76	.44	.45	.43	.76	.76	.43	.47
NC measures at 10 years								
English: speaking and listening	.73	.44	.48	.40	.78	.69	.50	.45
English: reading	.72	.46	.49	.42	.72	.71	.52	.46
English: writing	.73	.42	.46	.38	.73	.72	.49	.41
English: composite	.80	.49	.53	.45	.81	.78	.56	.50
Mathematics: using and applying	.72	.41	.45	.37	.71	.73	.50	.40
Mathematics: numbers and algebra	.72	.41	.43	.40	.69	.75	.46	.40
Mathematics: shapes, space and measures	.71	.42	.48	.37	.68	.74	.51	.45
Mathematics: composite	.76	.44	.49	.40	.74	.78	.52	.45
Science: scientific enquiry	.71	.47	.53	.41	.72	.71	.57	.48
Science: life processes	.72	.48	.54	.42	.72	.73	.58	.49
Science: physical processes	.72	.49	.56	.41	.71	.73	.60	.52
Science: composite	.76	.51	.57	.44	.75	.76	.62	.52
*2. Test measures*								
Reading tests at 7 years								
Towre: word	.83	.49	.52	.46	.85	.82	.54	.49
Towre: non-word	.80	.46	.50	.42	.81	.79	.51	.48
Towre: composite	.85	.50	.54	.45	.85	.84	.56	.52
Reading test at 10 years								
PIAT	.64	.44	.44	.44	.66	.63	.44	.43
Mathematics test at 10 years								
Understanding Number	.59	.38	.40	.36	.57	.60	.36	.43
Nonnumerical Processes	.56	.40	.40	.39	.58	.54	.41	.39
Computation and Knowledge	.52	.30	.37	.23	.52	.53	.35	.38
Mathematics: composite	.68	.44	.49	.37	.66	.70	.46	.52
*3. General cognitive ability*								
“*g*” at 7 years	.66	.48	.49	.47	.68	.64	.52	.48
“*g*” at 9 years	.76	.59	.61	.57	.75	.77	.56	.65
“*g*” at 10 years	.72	.51	.54	.47	.72	.72	.53	.55

*Note*.—All correlations were significant at *p*<.01.

The numbers of pairs were as follows:

NC measures at 7 years: MZ=1888–1944; DZall=3385–3474.

NC measures at 9 years: MZ=877–915; DZall=1547–1620.

NC measures at 10 years: MZ=913–954; DZall=1612–1676.

Tests at 7 years (TOWRE and General Cognitive Ability): MZ=1769–1791; DZall=3107–3166.

Tests at 9 years (General Cognitive Ability): MZ=1139; DZall=1265.

Tests at 10 years (PIAT, Mathematics and General Cognitive Ability): MZ=722–931; DZall=1265–1610.

MZ, monozygotic; DZall, dizygotic same-sex and opposite-sex twins; DZss, same-sex dizygotic twins; DZos, opposite-sex dizygotic twins; MZM, monozygotic male twins; MZF, monozygotic female twins; DZM, dizygotic male twins; DZF, dizygotic female twins.

**TABLE 11:** ACE MODEL-FITTING ESTIMATES WITH 95% CONFIDENCE INTERVALS IN PARENTHESES^a^

Measure	RMSEA	A	C	E
*1. NC measures*				
NC measures at 7 years				
English: speaking and listening	.023	.60 (.54–.65)	.20 (.15–.24)	.21 (.19–.22)
English: reading	.014	.68 (.62–.74)	.07 (.02–.12)	.25 (.23–.27)
English: writing	.018	.65 (.58–.70)	.03 (.00–.09)	.32 (.30–.34)
English: composite	.007	.65 (.60–.71)	.17 (.12–.22)	.18 (.17–.19)
Mathematics: using and applying	.034	.65 (.59–.72)	.07 (.01–.12)	.28 (.26–.30)
Mathematics: numbers and algebra	.025	.64 (.57–.70)	.06 (.01–0.12)	.30 (.28–.32)
Mathematics: shapes, space and measures	.024	.66 (.60–.72)	.09 (.04–.15)	.25 (.23–.27)
Mathematics: composite	.039	.68 (.63–.74)	.09 (.04–.15)	.22 (.21–.24)
NC measures at 9 years				
English: speaking and listening	.024	.51 (.41–.60)	.17 (.09–.25)	.32 (.29–.35)
English: reading	.031	64 (.56–.73)	.10 (.02–.18)	.25 (.23–.28)
English: writing	.007	.70 (.61–.75)	.02 (.00–.10)	.28 (.25–.31)
English: composite	.020	.67 (.59–.76)	.11 (.04–.19)	.21 (.19–.23)
Mathematics: using and applying	.027	.73 (.64–.76)	.01 (.00–.09)	.26 (.24–.29)
Mathematics: numbers and algebra	.031	.67 (.58–.74)	.04 (.00–.12)	.29 (.26–.32)
Mathematics: shapes, space and measures	.031	.63 (.54–.72)	.09 (.01–.17)	.28 (.25–.31)
Mathematics: composite	.022	.72 (.64–.79)	.04 (.00–.12)	.23 (.21–.26)
Science: scientific enquiry	.013	.58 (.49–.67)	.13 (.04–.21)	.29 (.27–.32)
Science: life processes	.010	.65 (.56–.74)	.09 (.00–.17)	.27 (.24–.29)
Science: physical processes	.000	.65 (.56–.75)	.08 (.00–.16)	.27 (.24–.30)
Science: composite	.012	.63 (.55–.72)	.12 (.04–.20)	.24 (.22–.27)
NC measures at 10 years				
English: speaking and listening	.034	.56 (.47–.65)	.17 (.09–.24)	.28 (.25–.30)
English: reading	.016	.52 (.43–.61)	.20 (.12–.27)	.28 (.26–.31)
English: writing	.044	.64 (.55–.72)	.10 (.02–.17)	.27 (.24–.30)
English: composite	.020	.60 (.52–.67)	.20 (.12–.26)	.21 (.19–.23)
Mathematics: using and applying	.041	.63 (.54–.72)	.09 (.01–.17)	.28 (.25–.31)
Mathematics: numbers and algebra	.034	.62 (.53–.71)	.10 (.02–.18)	.28 (.25–.31)
Mathematics: shapes, space and measures	.047	.59 (.50–.68)	.13 (.05–.21)	.28 (.26–.31)
Mathematics: composite	.044	.64 (.56–.72)	.12 (.04–.19)	.24 (.22–.26)
Science: scientific enquiry^b^	.046	.48 (.39–.56)	.23 (.16–.31)	.29 (.27–.32)
Science: life processes	.039	.48 (.40–.57)	.24 (.16–.31)	.28 (.25–.31)
Science: physical processes	.047	.45 (.36–.53)	.27 (.19–.34)	.29 (.26–.32)
Science: composite	.047	.48 (.41–.56)	.27 (.19–.34)	.25 (.22–.27)
*2. Test measures*				
Reading tests at 7 years				
Towre: word	.013	.69 (.63–.74)	.15 (.10–.20)	.17 (.16–.18)
Towre: non-Word	.025	.67 (.61–.73)	.13 (.07–.18)	.20 (.19–.22)
Towre: composite	.030	.70 (.64–.75)	.15 (.10–.20)	.15 (.14–.17)
Reading test at 10 years				
PIAT	.012	.39 (.28–.50)	.25 (.15–.34)	.36 (.33–.41)
Mathematics test at 10 years:				
Understanding Number	.008	.41 (.30–.52)	.18 (.09–.26)	.41 (.38–.46)
Nonnumerical Processes	.009	.33 (.22–.44)	.23 (.14–.31)	.44 (.40–.48)
Computation and Knowledge	.020	.46 (.34–.56)	.07 (.00–.17)	.47 (.43–.52)
Mathematics: composite	.024	.49 (.40–.58)	.19 (.11–.27)	.32 (.29–.35)
*3. General cognitive ability*				
“*g*” at 7 years	.002	.36 (.29–.42)	.30 (.25–.36)	.34 (.32–.36)
“*g*” at 9 years	.030	.36 (.29–.42)	.41 (.35–.46)	.24 (.22–.26)
“*g*” at 10 years	.002	.41 (.33–.50)	.30 (.23–.37)	.28 (.26–.31)

*Note*.—

a A full sex-limitation model was used to test for quantitative (boy vs. girls) and qualitative (same-sex vs. opposite-sex DZ pairs), as explained in Chapter II. For all 43 analyses, no significant quantitative or qualitative sex differences emerged. Therefore, estimates are equated for boys and girls and for same-sex and opposite-sex DZ pairs, described in Chapter II as the “null” model.

b Although the best-fitting model was the null model for 42 analyses, the best-fitting model, for Scientific Enquiry at 10 years was the scalar model which allows variance differences between boys and girls but no quantitative or qualitative sex differences.

RMSEA root mean square error of approximation: √[*χ*^2^/df−1)/(*N*−1)], where *N* is the sample size and df the degrees of freedom of the model. Models that provide a good fit to the data have a RMSEA less than or equal to .05. A, additive genetic influence; C, shared environmental influence; E, non-shared environmental influence.

#### NC TEACHER RATINGS

The pattern of twin correlations in Table 10 for NC teacher ratings consistently suggests substantial genetic influence and modest shared and nonshared environmental influence across domains and across ages. Consider the first two columns of correlations, which are based on the entire sample of MZ twins and DZ twins. (The other columns show results separately by sex, which we will discuss later.) The first row shows twin correlations for English: Speaking and Listening at 7 years. The MZ and DZ correlations of .80 and .49 suggest substantial heritability of .62 [2 (rMZ−rDZ)], .18 shared environmental influence (rMZ−heritability), and .20 nonshared environmental influence (1−rMZ), which includes error of measurement.

A similar pattern of results is seen for all three components of English at 7 years, although Writing shows the least shared environmental influence (.05). A remarkably similar pattern of results can also be seen at 9 and 10 years for the three components of English. For the English composite, the twin correlations yield heritability estimates of .64, .64, and .62, respectively, at 7, 9, and 10 years; estimates of shared environmental influence are .18, .14, and .18.

Results are also similar for the Mathematics composite at 7, 9, and 10 years for heritability (.68, .70, and .64) and shared environment (.10, .06, .12). The Science composite also yielded similar results at 9 years (.64 heritability, .12 shared environment), but at 10 years it suggested somewhat less heritability (.50) and somewhat greater shared environment (.26), a pattern seen for all three components of Science at 10 years.

The model-fitting results for NC measures shown in Table 11 confirm these estimates and conclusions based on the twin correlations: Heritabilities are substantial and shared environmental estimates are modest within each domain, across domains, and across ages. For example, as noted above, the twin correlations for the first row of Table 10 suggested estimates of .62 for heritability, .18 for shared environment, and .20 for nonshared environment. The model-fitting estimates shown in Table 11 are .60, .20, and .21, respectively. The twin correlations suggested that Writing at 7 years showed less shared environmental influence (.05), and this is also confirmed with a model-fitting estimate of .03; the nonoverlapping confidence intervals in the shared environmental estimates indicate that the shared environmental estimate for Writing is significantly lower than for Speaking/Listening. Also, the twin correlations suggested that the Science composite at 10 years was less heritable (.50) and more influenced by shared environment (.26) than any of the other domains. Model-fitting estimates were similar (.48 and .27, respectively); however, the overlapping confidence intervals from the model-fitting analyses indicate that these differences are not significant for the composite measures. The average model-fitting heritability estimate across the NC composite scores and across age is .63, the average estimate of shared environment is .14 and the average estimate of nonshared environment is .22.

#### TESTS OF READING AND MATHEMATICS

The second panel of Table 10 presents MZ and DZ twin correlations for two subtests and a composite of the TOWRE at 7 years, the PIAT reading recognition test at 10 years, and three subtests and a composite of the Mathematics test at 10 years. The TOWRE at 7 years yields results similar to those for NC teacher ratings: heritability of .70 and shared environment of .15 for the composite measure. The word and nonword subtests of the TOWRE yield very similar results. However, the results for the PIAT at 10 years differ from the results for the NC teacher ratings and for the TOWRE: MZ correlations are lower, which results in somewhat lower heritability estimates (.40) and higher shared environment estimates (.24).

Twin correlations for the web-based mathematics tests also suggest moderate heritability (.48 for the composite) and modest shared environment (.20 for the composite). Results for the three component tests in the mathematics battery are similar.

The model-fitting results shown in the second panel of Table 11 confirm these conclusions for the tests of reading and mathematics. The confidence intervals indicate that the heritability of the TOWRE at 7 years is significantly greater than for the PIAT at 10 years.

#### TESTS OF GENERAL COGNITIVE ABILITY (“*g*”)

Despite the different modes of measurement for “*g*” at 7 (telephone), 9 (mailed booklets), and 10 (web-based tests), the results are similar across the three ages. Heritability estimates for “*g*” based on the twin correlations shown in the third panel of Table 10 are lower than for the measures of academic performance: .36 at 7 years, .34 at 9 years, and .42 at 10 years. Shared environmental estimates are higher than for the measures of academic performance: .30, .42, .30, respectively.

The model-fitting estimates shown in Table 11 are similar. Nonoverlapping confidence intervals suggest that at each age “*g*” is significantly less heritable and shows significantly more shared environmental influence than the NC composite measures except for Science at 10. One possibile explanation of the greater heritability of achievement scores is that the expression of genetic potential for achievement takes place in the context of active genotype-environment correlations, driven by variance in interest, motivation, and engagement. In other words, in respect to genetic influences on achievement, genes code for appetites, not aptitudes. Finding genes for both general cognitive ability and different areas of academic achievement will facilitate understanding the mechanisms that lead to the observed differences in heritability between *g* and achievement.

#### BOYS VERSUS GIRLS

So far we have focused on results for the entire sample without regard to sex. The last four columns of Table 10 show the twin correlations separately for boys and girls. In general across measures and ages, estimates derived from the twin correlations are similar for boys and girls. For example, for the NC ratings, the average heritability estimates are .56 for boys and .59 for girls; estimates of shared environment are .21 for boys and .19 for girls. The largest differences are for NC Mathematics and NC Science at 10 years where heritability is lower for boys than girls (.44 vs. .66 for NC Mathematics composite and .26 vs. .48 for NC Science composite) and shared environment is greater for boys than girls (.30 vs. .12 and .49 vs. .28). However, for the Mathematics test-score data at 10 years, the results are similar for both boys and girls: The average heritability estimates are .42 for boys and .38 for girls; shared environment estimates are .30 and .33.

These interpretations based on the twin correlations are confirmed by fitting sex-limitation models, which yielded no significant quantitative sex differences. The best-fitting sex-limitation model for all but one of the 43 analyses was the “null” model that allows no sex differences in parameter estimates.

In summary, ACE parameter estimates are similar for boys and girls in our most powerful analyses that capitalize on the entire TEDS dataset. Analyses in subsequent chapters are less powerful in that they involve sub-samples—sub-samples at the low extremes of the distribution (Chapter IV), sub-samples with longitudinal data at all three ages (Chapter V), and sub-samples with complete data on all measures for multivariate analyses (Chapter VI). In order to maximize power for these analyses and to simplify our presentation of the results, we will focus on analyses of the total dataset with sexes combined.

#### SAME-SEX VERSUS OPPOSITE-SEX DZ TWINS

Table 10 shows that twin correlations are similar for same-sex and opposite-sex DZ twins. On average, correlations for the same-sex DZ twins are .06 greater than for opposite-sex DZ twins for NC ratings, reading and mathematics tests, and “*g*.” Even with these large sample sizes, such differences are not nearly significant. As expected from these results, sex-limitation model fitting shows no significant qualitative sex differences. Because same-sex and opposite-sex DZ twins yield similar results, subsequent analyses will maximize power by combining same-sex and opposite-sex DZ twins.

#### SAME TEACHERS VERSUS DIFFERENT TEACHERS

As mentioned in Chapter II, some twins were in the same classroom: 67% at 7 years, 63% at 9 years, and 58% at 10 years. For the teacher ratings, when twins were in the same classroom, they were rated by the same teacher; when they were in different classrooms, they were rated by different teachers. In this section, we examine the effect of having the same versus different teachers on ACE estimates.

##### Teacher Ratings

In general, twin correlations were higher when the same teacher rather than different teachers rated members of a twin pair. However, differences in twin correlations for MZ and DZ twins were similar for same-teacher and different-teacher ratings. In other words, heritability estimates were similar regardless of whether the same teacher or two different teachers rated members of a twin pair, but shared environment estimates were greater when twins were rated by the same teacher. For example, for the English composite at 7 years, the MZ and DZ_all_ correlations for same-teacher ratings were .88 and .55; for different-teacher ratings the correlations were .71 and .39. These twin correlations suggest heritabilities of .66 for same-teacher ratings and .64 for different-teacher ratings. Shared environment estimates were greater for same-teacher ratings (.22) than for different-teacher ratings (.07).

Similar results were obtained for all of the NC ratings at all three years: The average heritability estimates were .64 for same-teacher ratings and .59 for different-teacher ratings; the average shared environment estimates were .22 and .05, respectively. Differences of this magnitude in heritabilities and shared environment were not significantly different in model-fitting analyses, despite the large sample size.

###### Tests

Although the estimates of shared environment for same-teacher ratings as compared with different-teacher ratings are not significantly different, the somewhat higher shared environment estimates for same-teacher ratings might signal a bias when one teacher rates both twins. However, children rated by the same teacher have been in the same classroom and experiencing the same instruction, so another possibility is that they might show a true shared environmental effect as compared with children in different classrooms. These hypotheses can be tested by comparing the results for NC ratings to those for test data. If the rating bias hypothesis is correct, test data should not show a difference in shared environment estimates for twins in the same classroom versus twins in different classrooms. On the other hand, if the twins in the same classroom truly evidence more effect of shared environment than children in different classrooms for NC teacher ratings, the test data should show the same pattern of results.

For three of the tests, the results appear to support the rating bias hypothesis: Averaged across the three ages, the shared environmental estimates for children in the same classroom versus in different classrooms were, respectively, .24 and .24 for the PIAT, .17 and .21 for the web-based Mathematics composite, and .35 and .31 for “*g*.” In contrast, the results for the TOWRE at 7 years appear to support the second hypothesis: Shared environment estimates for twin pairs taught by the same teacher in the same classroom and those taught by different teachers in different classrooms were .17 and .07 for the TOWRE composite, although this difference in estimates of shared environment is not nearly significant.

Averaging estimates of shared environment across all four tests at all ages, the test-score data yield highly similar estimates for twins with the same teacher (.27) and those with different teachers (.24), suggesting that there is no added shared environmental effect when children share the same classroom. Taken together, these findings for the test-score data support the rating bias interpretation of the teacher-rating results (when one teacher rates both twins, estimates of shared environment are inflated).

However, there is another twist: The results for the test-score data do not support the most straightforward version of the rating bias hypothesis, because the average shared environment estimate for test-score data across ages (.22) is similar to the average shared environment estimate for same-teacher ratings (.20), not to the average estimate for different-teacher ratings (.07) as might have been expected. Thus, same-teacher ratings of academic performance do not inflate estimates of shared environment as compared with test-score data. Instead, it is possible that different-teacher ratings inflate estimates of nonshared environment by introducing between-rater variance. For example, although it seems reasonable to assume that the results for different-teacher assessments are more valid as they eliminate rating bias, seeing the two twins together all day long may make same-teacher ratings more valid. However, a less interesting, but more parsimonious, hypothesis is that the nonsignificant differences in shared environmental estimates for NC ratings by same versus different teachers are not real.

As was the case for same-teacher and different-teacher NC ratings, heritability estimates for the test-score data were similar for members of twin pairs, whether taught by the same teacher or by different teachers: .68 and .78 for TOWRE composite, .38 and .42 for PIAT, .52 and .46 for web-based Mathematics composite, and .35 and .41 for “*g*” on average across the 3 years.

Twin correlations and model-fitting estimates for same- and different-teacher ratings are available from the authors. Because results are generally similar for twins taught by the same teacher and those taught by different teachers, subsequent analyses were conducted on the combined sample in order to maximize power and simplicity of presentation.

#### SUMMARY

Individual differences in early academic performance show substantial genetic influence and modest shared environmental influence. The magnitude of genetic influence—about 65% for year-long teacher assessments based on U.K. NC criteria and about 55% for test data—is surprising. Heritabilities are greater for academic performance than for general cognitive ability (35% on average). We were also surprised to find such consistently high heritabilities of academic performance at 7, 9, and 10 years despite major changes in content across these years. Our hypothesis was not confirmed that heritability would increase during the early school years as skills training developed into the application of these skills—for example, from learning to read to reading to learn. Even though heritabilities are as high at 7 years as at 9 years, it is possible that the genetic correlates of academic performance reflect changing patterns of component skills from 7 to 9 years. We will return to this issue in Chapter VI. High heritabilities were not only found across all three ages but also across the components of each domain (e.g., for English: Speaking and Listening, Reading, and Writing) and across domains (English, Mathematics, and Science). However, this finding does not imply that the same genetic factors affect these diverse domains of academic performance; multivariate genetic analysis is needed to address this issue (see Chapter VI). An interesting sideline is that estimates of heritability were similar for teacher assessments when the same teacher assesses members of a twin pair and when different teachers assess them. The similarity of results across domains, across ages, and across methods of assessment indicates the robustness of these findings.

Just as surprising is the modest role for shared environmental influence for pairs of children growing up in the same family and being taught in the same school, often by the same teacher in the same classroom. Measures of general cognitive ability showed more shared environmental influence than do teacher assessments and tests of academic performance. Nevertheless, nonshared environment accounts for more variance than shared environment, although it should be acknowledged again that nonshared environment also includes variance due to measurement error.

Results are similar for boys and girls, as well as for same-sex and opposite-sex DZ twins. These results suggest that quantitative and qualitative sex differences do not play a major role in the origins of individual differences in learning abilities.

These results raise several questions to which we will return in the final chapter. For example, why do teacher ratings of academic performance show greater genetic influence than test scores? Why do tests of academic performance show more genetic influence and less shared environmental influence than measures of general cognitive ability? Why is the TOWRE measure of word recognition at 7 years significantly more heritable than the PIAT reading recognition at 10 years?

Although these analyses of genetic and environmental influences on individual differences in learning abilities in the early school years have yielded some surprising results, the main goal of this monograph is to go beyond these rudimentary issues of nature and nurture in order to address the relationship between the normal and abnormal, longitudinal analyses of stability and change, and multivariate analyses of covariance within and between domains. These are the topics of the next three chapters.

### IV. THE ABNORMAL IS NORMAL

The previous chapter investigated the sources of individual differences throughout the normal distribution, which we refer to as learning abilities. In this chapter, we focus on the lower end of the distribution, learning disabilities. To what extent are learning disabilities etiologically distinct from the normal range of variation? Our hypothesis follows from Quantitative Trait Locus (QTL) theory, which posits that genetic influence on common disorders and complex traits is caused by many genes (loci) of small effect rather than by one gene or even by a few genes of large effect (Plomin, Owen, & McGuffin, 1994). Unlike single-gene effects, that are necessary and sufficient for the development of a disorder, QTLs contribute interchangeably and additively as probablilistic risk factors. If QTL theory is correct, common disorders such as learning disabilities are likely to be the quantitative extreme of the same genetic factors responsible for variation throughout the distribution. The QTL model refers to quantitative traits even in relation to disorders because if many genes affect a disorder, then it necessarily follows that there will be a quantitative distribution rather than a dichotomy. Stated more provocatively, there is no disability, just low ability—the abnormal is normal. The QTL model is discussed in greater detail in Chapter VII. The ultimate proof of the QTL model will come when QTLs identified for learning disabilities are found to be associated with the normal range of variation in abilities and vice versa.

With a large and representative twin sample like TEDS, it is possible to study disabilities in the context of abilities. We can compare estimates of genetic and environmental influence for abilities and for disabilities in the same sample. Finding that genetic or environmental estimates differ for abilities and disabilities indicates that there are etiological differences between abilities and disabilities. However, differences in the magnitude of genetic and environmental estimates for abilities and disabilities are merely quantitative differences, not qualitative differences. That is, even if heritability differed quantitatively for disabilities and abilities, the same genes could nonetheless be associated with disabilities and abilities. Conversely, heritabilities could be the same for disabilities and abilities and yet different genes could be associated with disabilities and abilities.

What we would most like to know is whether there are qualitative differences between disabilities and abilities. That is, are genes associated with learning disability different from the genes associated with normal variation in ability? As discussed in Chapter II, we believe that DF extremes analysis addresses this issue of qualitative differences directly. Finding group heritability implies genetic links between disabilities and abilities, and finding group shared environment implies shared environmental links between disabilities and abilities.

The structure of this chapter is similar to the previous chapter. However, instead of presenting twin correlations and ACE model fitting for the entire sample, this chapter focuses on twin concordances, twin “group” correlations, and DF extremes analyses for children with low scores. For reasons discussed in the previous chapter, we maximized power for these extremes analyses by combining boys and girls, same-sex, and opposite-sex DZ twins, and assessments by same and different teachers. Furthermore, because results presented in the previous chapter were so similar for components of each domain, the present chapter simplifies presentation of results by focusing on the composite scores for each domain at each age.

We selected twin pairs for whom at least one twin scored in the lowest 15% on the composite score. We have also conducted analyses using a 5% cut-off and generally find similar results. We have chosen to present results for the 15% cut-off for three reasons. First, performance one standard deviation below the mean, which corresponds to a 15.9% cut-off in a perfectly normal distribution, is often used as a cut-off for common disorders. Second, for the U.K. National Curriculum (NC), a 15% cut-off corresponds to children identified as performing below their grade expectation and failing items that are solved correctly by the majority of much younger children (Kovas, Haworth, Petrill, & Plomin, in press). Third, in TEDS, a 15% cut-off strikes a balance between extremity of scores and sample size needed to attain reasonable power in DF extremes analysis. The number of probands in the lowest 15% of the distribution for each measure and the number of MZ and DZ pairs with at least one proband are listed in the footnote to Table 12. In our individual differences analyses in the previous chapter, we excluded all twin pairs in which one or both twins scored 3 or more standard deviations below or above the mean so that our individual differences results would not be affected by very extreme scores. However, children with low extreme scores were restored for the present analyses because our focus here is on children with low scores and because DF extremes analysis is an analysis of means rather than variances and analyses of means are not inordinately affected by outliers.

**TABLE 12:** MZ AND DZ PROBANDWISE CONCORDANCES AND RESULTS OF DF EXTREMES ANALYSIS USING 15% CUTOFFS

	Probandwise concordance		Twin group correlation		DF estimates	
	MZ	DZ	MZ	DZ	h^2^g (SE)	c^2^g (SE)
7-year NC English	.76	.44	.85	.47	.75 (.07)	.10 (.05)
7-year NC Math	.73	.46	.81	.46	.69 (.08)	.11 (.06)
9-year NC English	.71	.38	.82	.40	.83 (.09)	.00 (.07)
9-year NC Math	.68	.36	.75	.31	.89 (.10)	.00 (.07)
9-year NC Science	.77	.44	.79	.42	.75 (.10)	.04 (.07)
10-year NC English	.71	.37	.82	.40	.84 (.09)	.00 (.07)
10-year NC Math	.70	.40	.79	.42	.75 (.10)	.04 (.07)
10-year NC Science	.68	.46	.76	.46	.58 (.11)	.17 (.08)
7-year TOWRE	.71	.47	.88	.55	.65 (.06)	.23 (.05)
10-year PIAT	.48	.36	.63	.42	.43 (.11)	.20 (.08)
10-year Web Math	.46	.36	.64	.40	.47 (.10)	.16 (.07)
7-year *g*	.52	.38	.68	.47	.42 (.07)	.26 (.05)
9-year *g*	.60	.44	.73	.55	.37 (.08)	.37 (.06)
10-year *g*	.57	.36	.72	.46	.52 (.09)	.20 (.07)

*Note*.—7-year NC English: 1,170 families (1,632 probands) 392 MZ pairs, 778 DZ pairs.

7-year NC Math: 1,174 families (1,634 probands) 386 MZ pairs, 788 DZ pairs.

9-year NC English: 566 families (756 probands) 183 MZ pairs, 383 DZ pairs.

9-year NC Math: 554 families (732 probands) 198 MZ pairs, 356 DZ pairs.

9-year NC Science: 531 families (739 probands) 172 MZ pairs, 359 DZ pairs.

10-year NC English: 580 families (775 probands) 197 MZ pairs, 383 DZ pairs.

10-year NC Math: 571 families (769 probands) 193 MZ pairs, 378 DZ pairs.

10-year NC Science: 560 families (772 probands) 207 MZ pairs, 353 DZ pairs.

7-year TOWRE: 1,063 families (1,476 probands) 369 MZ pairs, 694 DZ pairs.

10-year PIAT: 470 families (589 probands) 176 MZ pairs, 294 DZ pairs.

10-year Web Math: 615 families (768 probands) 221 MZ pairs, 394 DZ pairs.

7-year *g*: 1,142 families (1,461 probands) 429 MZ pairs, 713 DZ pairs.

9-year *g*: 674 families (901 probands) 247 MZ pairs, 427 DZ pairs.

10-year *g*: 580 families (745 probands) 214 MZ pairs, 366 DZ pairs.

MZ, monozygotic; DZ, dizygotic; h^2^g, group heritability; c^2^g, group shared environment.

Table 12 summarizes the results in terms of MZ and DZ probandwise concordances, twin group correlations, and DF extremes estimates of group heritability and group shared environment. These results are examined in the following sections.

#### NC TEACHER RATINGS

The concordances and twin group correlations (see Chapter II) yield similar results for all NC domains at all ages. For example, for the first row of results (7-year NC English), the much greater MZ concordance (76%) than DZ concordance (44%) suggests substantial heritability. The pattern of MZ and DZ concordances also suggests little influence of shared environment, especially when the base rate of 15% is taken into account. As explained in Chapter II, concordances are based on dichotomous data (affected or not), and cannot in themselves be used to estimate genetic and environmental parameters unless they are converted into liability correlations.

DF extremes analysis uses quantitative trait data to produce twin group correlations that indicate the extent to which co-twins of probands resemble the probands on a quantitative trait. For example, for 7-year NC English, the MZ group correlation of .85 indicates that the mean of the co-twins of MZ probands is 85% below the population mean as compared with the probands. That is, the mean standard score for both MZ and DZ probands for all measures is −1.6. The mean standard score of the MZ co-twins is −1.3, very similar to the proband mean. Doubling the difference between the MZ and DZ group correlations of .85 and .47 suggests a group heritability of .76. Group shared environment can be estimated as the extent to which group heritability does not explain MZ similarity: .85−.76=.09. These estimates are highly similar to those derived from DF extremes regression analysis shown in Table 12 (i.e., .75 for group heritability and .10 for group shared environment). Doubling the standard errors of these estimates (see Table 12) indicates that group heritabilities are statistically significant but that group shared environment is not.

Across the eight NC composite measures at 7, 9, and 10 years, the average group heritability estimate is .76 and the average group shared environment estimate is .06. As explained in Chapter II, it should be emphasized that these group statistics refer to group means, not to individual differences within the extreme group. For example, a group heritability of .76 means that 76% of the difference between the proband and population means can be attributed to genetic influences.

#### TESTS OF READING AND MATHEMATICS

As compared with NC teacher ratings, the test scores yield lower estimates of group heritability and higher estimates of group shared environment. For the TOWRE at 7 years, group heritability is .65 and group shared environment is .23. Group heritabilities are also significantly lower than those for the NC teacher ratings for the web-based tests of reading and mathematics at 10 years (.43 and .47, respectively); group shared environments are .20 and .16.

#### TESTS OF GENERAL COGNITIVE ABILITY (“*g*”)

The results for “*g*” at 7, 9, and 10 are similar to those for the web-based tests of reading and mathematics, despite the different modes of measurements of “*g*” at 7 (telephone), 9 (mailed booklets), and 10 (web-based tests). Group heritabilities range from .37 to .52 and group shared environments range from .20 to .37.

#### DISABILITIES AND ABILITIES

**FIGURE 6:**
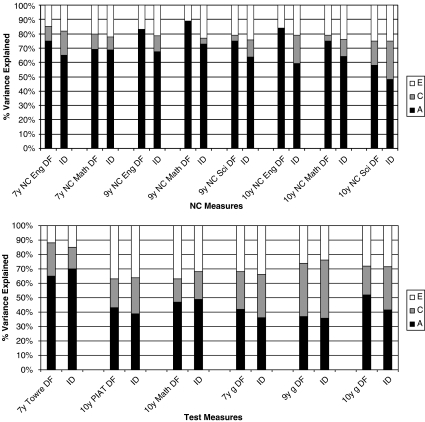
—Comparison between genetic and environmental estimates for the lowest-performing 15% (DF extremes analyses from this chapter) and for individual differences throughout the entire distribution (Chapter III). The top panel compares extremes and the entire distribution for NC teacher assessments; the bottom panel summarizes results for the reading, mathematics and “*g*” test data.

These results support the hypothesis that the abnormal is normal, both quantitatively and qualitatively. Evidence for the quantitative similarity of genetic and environmental estimates for the abnormal and normal can be seen in Figure 6, which summarizes visually the DF extremes results reported in this chapter and compares them with the individual differences results from the previous chapter. For example, in the previous chapter, the average model-fitting estimate of heritability across the NC composite scores and across age was .63, the average-shared environment was .14 and the average of non-shared environment was .22. In the present chapter, across the same NC composite scores and ages, DF extremes analyses yielded an average group heritability of .76, an average group shared environment of .06, and an average group nonshared environment of .18. The test measures of reading and mathematics and the measures of “*g*” at the three ages also yield similar results for disability and ability. The slight differences in results for the low extremes and the whole sample are well within their 95% confidence intervals. The similarity in results is especially remarkable because the analyses are so different. DF extremes analysis is based on means for the probands, the co-twins, and the population in which probands were selected from the lowest 15% of the distribution, whereas analyses from the previous chapter are based on individual differences throughout the distribution.

If ACE estimates had differed for disabilities and abilities, this would indicate an etiological difference between them. However, as noted earlier and explained in Chapter II, such quantitative differences, for example in heritability, could be due to the same genes affecting disabilities and abilities but differing in the magnitude of their effect at the low end of the distribution. This could occur, for example, if shared environmental influences had a stronger effect at the low end of the distribution.

In the present situation in which similar quantitative ACE estimates were found for disabilities and abilities, it is possible that different genes are associated with disabilities and abilities even though the net effects of such genes are of a similar magnitude for disabilities and abilities. As mentioned earlier, what we would like to know is whether there are qualitative differences between disabilities and abilities; that is, whether different genes or different environmental factors affect disabilities and abilities. We suggest that DF extremes analysis itself, not the comparison between the results for DF extremes analysis and analysis of individual differences, speaks to qualitative differences between disabilities and abilities. Although this is a complicated issue (for details, see Plomin & Kovas, 2005), group heritability and group shared environment can be observed only to the extent that there are links between disability and ability. That is, if the measure of disability is unrelated to the measure of ability, there can be no group heritability or group shared environment—the co-twins' mean would regress back to the population mean in DF extremes analysis. However, finding genetic and environmental links between disability and ability does not imply that all effects are in common. Indeed it is likely that there are rare single-gene effects and rare environmental trauma that lead to learning disability but account for little variance in the population as a whole (Plomin & Kovas, 2005).

Thus, we conclude that the results presented in this chapter are consistent with the hypothesis that the abnormal is normal both quantitatively and qualitatively. The strongest test of the hypothesis that the abnormal is normal will come when genes are found that are associated with disabilities or abilities. Our prediction is that any gene associated with reading disability, for example, will also be associated with individual differences in reading ability throughout the distribution, including good readers. This hypothesis is consistent with QTL theory, which posits that common disorders are the quantitative extreme of the same genetic factors that create variation throughout the distribution.

It is possible that these conclusions do not apply to more severe forms of disability identified by different criteria and more severe cut-offs than the ones used in this study. However, as discussed earlier, the cut-off for disability used in this study selected children with very low performance, failing items that are successfully solved by the majority of younger children. Also, as discussed earlier, we repeated all analyses with a more severe cut-off of 5% of the whole sample, and obtained very similar results. Finally, it is possible that some rare variants of learning disabilities have a distinct etiology from that of most common disabilities, as discussed in the concluding chapter.

Although the results reported in this chapter have no immediate implications for teaching or for remediating disabilities, we believe that it is important for parents, teachers and policy makers to recognize that common learning disabilities are etiologically the low end of quantitative continua of ability, rather than being driven by unique genetic and environmental factors. This finding has far-reaching implications for defining learning disability as well as for research into factors that are responsible for learning disability, as discussed in Chapter VII.

#### SUMMARY

ACE results for the lowest 15% of children at each age for all measures are remarkably similar to the individual differences results presented in the previous chapter for the entire distribution. That is, for disability as well as for ability, heritability for NC teacher ratings is very high (≈.70) and shared environment is very low (≈.10). For web-based measures of reading and mathematics at 10 years as well as for tests of “*g*” at all three ages, disability as well as ability shows less heritability (≈.40) and more shared environmental influence (≈.20).

The similarity of ACE results for disability and ability indicates that the quantitative etiologies of disability and ability are similar. The group heritability estimates suggest that the etiologies of disability and ability are also similar qualitatively.

### V. GENETIC STABILITY, ENVIRONMENTAL CHANGE

In this chapter, we go beyond estimating heritability by investigating the causes of change and continuity in the development of individual differences in learning abilities during middle childhood. There are two types of questions that can be asked using the twin method: quantitative differences in genetic and environmental influences on a trait, and qualitative changes in those influences. In the former, we ask whether the magnitude of genetic and environmental effects differs from age to age. This question can be addressed to a limited extent using cross-sectional data, although longitudinal data are better because the same sample is assessed across ages and therefore cohort differences are eliminated. The question of qualitative changes is the extent to which the same genetic and environmental effects operate across ages. Such analyses of age-to-age change and continuity require longitudinal data.

As in the previous chapter, we have maximized power by combining boys and girls, same-sex and opposite-sex DZ twins, and assessments by same and different teachers. We have also, for reasons of length and clarity of interpretation, reported results only for composite scores at each age. Unlike the previous chapter, which focused on the lower 15% of the distribution, the present chapter primarily reports analyses for the entire sample, again for reasons of power. However, we also present an example of a longitudinal extremes analysis of genetic and environmental contributions to change and continuity for reading disability at 7 and 10 years.

#### QUANTITATIVE AGE DIFFERENCES IN ETIOLOGY

**FIGURE 7:**
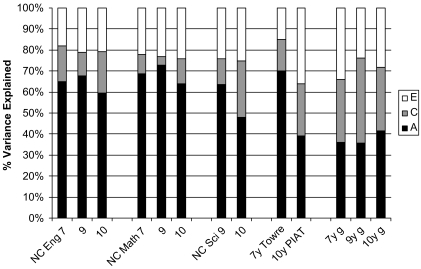
—Quantitative age differences in ACE parameter estimates at 7, 9, and 10 years

Chapter III included estimates of genetic and environmental influence at 7, 9, and 10 years. Figure 7 presents the main results, organized to facilitate comparisons across age for each composite measure. For example, for the NC English composite, heritabilities were similar at 7 (.65), 9 (.67), and 10 (.60) years. Shared environment estimates were also similar across the ages (.17, .11, and .20). Comparably stable estimates can be seen for the NC mathematics composite at 7, 9, and 10 years. NC Science between 9 and 10 years showed some difference in heritability (.64 vs. .50) and shared environment (.12 vs. .26), although these differences were not significant as indicated by their overlapping 95% confidence intervals (see Table 11).

ACE estimates were also stable for “*g*.” The only significant difference for age in Figure 7 is the difference in heritability between TOWRE at 7 years (.70) and PIAT at 10 years (.39). However, these two tests are very different: TOWRE at 7 years is a brief telephone-administered test of word recognition, whereas the PIAT at 10 years is a web-based test of reading comprehension. Thus, the ACE differences between these two measures might be due to measurement differences in content or method rather than age differences.

We were surprised to find such similar ACE results for learning abilities across ages despite major changes in content and complexity of the curriculum from 7 to 10 years (a transition between the two key stages on U.K. National Curriculum). Although 3 years represents a third of these children's life span, it may be too short a time to assess quantitative changes. For example, it is well documented that the heritability of “*g*” increases almost linearly during development—about 20% in infancy, 30% in middle childhood, 40% in adolescence, 50% in young adulthood, 60% in middle adulthood, and 70% in late adulthood (Boomsma, 1993; McGue, Bouchard, Jr., Iacono, & Lykken, 1993; Plomin, 1986). In our study, heritability of “*g*” was .36 at 7, .36, and 9 and .41 at 10, remarkably similar results despite the very different methodologies used to assess “*g*” at 7 (telephone), 9 (booklets), and 10 (internet) (Davis, Arden, & Plomin, 2007). Taking a longer view of development, we find the expected increase in heritability from early childhood to middle childhood. At 2–4 years, the average heritability of “*g*” was .26 (Spinath, Ronald, Harlaar, Price, & Plomin, 2003) as compared with the present average heritability of .38 at 7, 9, and 10 years. However, the TEDS measures of “*g*” in early childhood are very different from the measures used in middle childhood; for this reason, the apparent age difference could be due to the difference in measures.

These results indicating little age difference in ACE estimates raise the problem of power in detecting age differences in genetic and environmental parameter estimates. As mentioned earlier, for NC Science, heritability estimates and 95% confidence intervals are .63 (.55–.72) at 9 years and .48 (.41–.56) at 10 years. For shared environment, the estimates are .12 (.04–.20) and .27 (.19–.34). Even these relatively large differences in genetic and environmental parameter estimates are not quite statistically significant despite the relatively large sample size. Only heritability estimates that differ by as much as .20 (e.g., .65 vs. .45) would be detected as significantly different with these sample sizes. The only significant difference in Table 11 is between the heritability estimates for TOWRE at 7 years and PIAT at 10 years; these heritability estimates differ by .31. Moreover, significance only represents 50% power, which means that a difference of this magnitude would not be detected as significant half of the time.

#### QUALITATIVE AGE CHANGES IN ETIOLOGY

The longitudinal design of TEDS makes it possible to go beyond these essentially cross-sectional comparisons in ACE parameter estimates to investigate genetic and environmental influences on age-to-age change and continuity. As explained in Chapter II, longitudinal genetic analysis is a special case of multivariate genetic analysis, a technique that analyzes the covariance between traits rather than the variance of each trait separately. Longitudinal genetic analysis addresses the covariance across age for the same trait and decomposes the covariance (which indexes continuity) as well as the variance that does not covary (which indexes change) into genetic and environmental sources. In other words, longitudinal genetic analysis asks the extent to which genetic and environmental factors mediate phenotypic continuity and change from age to age. The statistic *bivariate heritability* describes the extent to which genetic factors account for the phenotypic correlation between two traits in multivariate genetic analysis or between the same trait at two ages in longitudinal genetic analysis.

A second question that longitudinal and multivariate genetic analyses address is the extent to which the same genetic and environmental factors operate across age, independent of the magnitude of their effect on the phenotype. For example, regardless of the heritability of a trait at two ages, to what extent do the genes that affect that trait at one age also affect the trait at another age? This second statistic is the *genetic correlation*. Comparable longitudinal correlations can be derived for shared and nonshared environment.

Longitudinal genetic analysis begins with phenotypic stability. If there is no phenotypic stability, genetic, and environmental influences at one age are independent of those at a second age. If there were no phenotypic stability, all genetic and environmental influences would contribute to change and the analysis reduces to separate analyses of genetic and environmental influences at each age. For learning abilities, age-to-age phenotypic correlations are substantial from 7 to 9, from 9 to 10, and even from 7 to 10. For NC English, the correlations are .63, .68, and .62, respectively; for NC mathematics, the correlations are .58, .63, and .55; for NC science from 9 to 10, the correlation is .49. Stability between the TOWRE word recognition test at 7 years and the PIAT reading comprehension test at 10 years is .44, but this lower stability might reflect differences in the measures. Similarly moderate stability is seen for “*g*” (.42 from 7 to 9, .55 from 9 to 10, and .40 from 7 to 10) despite the differences in measurement at 7 (telephone), 9 (booklet), and 10 (internet). A full phenotypic matrix across measures and across ages is included as Appendix E.

##### Longitudinal Model-Fitting Results

Longitudinal genetic analysis is based on cross-age twin correlations in which, for example, one twin's English score at 7 years is correlated with the co-twin's English score at 9 years. Genetic mediation of stability is indicated to the extent that cross-age twin correlations are greater for MZ than DZ twins. Rather than presenting the cross-age twin correlations, we have summarized the results visually, as derived from fitting a longitudinal model called Cholesky decomposition, which is described and illustrated in Chapter II.

**FIGURE 8:**
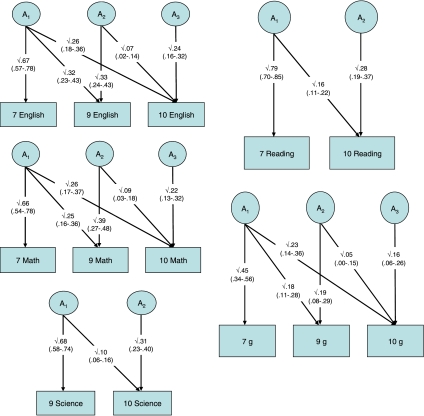

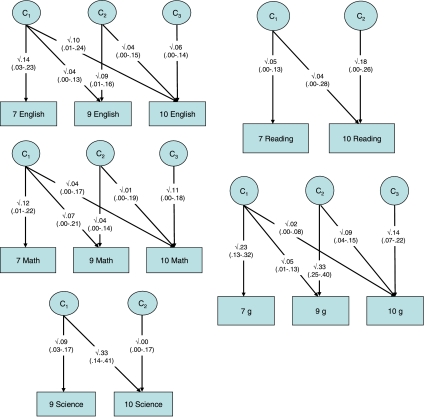

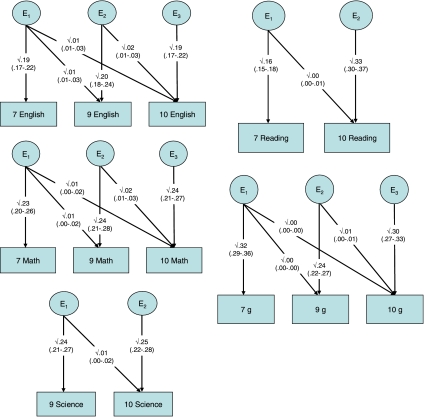
—Results of longitudinal genetic model-fitting analyses, presented separately for genetic (A), shared environmental (C), and nonshared environmental (E) influences (95% confidence intervals in parentheses). See text for explanation.

The model-fitting results for our longitudinal genetic analyses are shown in Figure 8, for genetic (A), shared environmental (C), and nonshared environmental (E) influences. The first factor A_1_ captures genetic influences in common across 7, 9, and 10 years. The loadings (95% confidence intervals in parentheses) indicate the variance of each variable that is accounted for by that factor. For the NC English composite, all of the genetic variance at 7 years is assigned to the first genetic factor −.67 is the heritability of English at 7 years in this longitudinal analysis, which is similar to the cross-sectional estimate of .65 shown in Table 11. (The square root sign is shown before .67 because the path coefficient itself, which is a standardized partial regression, is actually the square root of .67, i.e., .82. As seen below, it is easier to interpret the results using these squared path coefficients.) The significant and substantial loadings of English at 9 and 10 years on this general factor (A_1_) indicate strong genetic continuity. Of the genetic variance at 9 years (.32+.33=.65), about half (.32÷.65=49%) is shared with genetic variance at 7 years. Of the genetic variance at 10 years (.26+.07+.24=.57), almost half (.26÷.57=46%) is shared with genetic variance at 7 years.

Beyond this strong general genetic factor (A_1_), most of the remaining genetic variance is unique to each age, signaling genetic change. However, the second genetic factor (A_2_) indicates that some of the new genetic variance that emerges at 9 years contributes to continuity at 10 years. That is, 12% (i.e., .07÷.57) of the genetic variance at 10 years is shared with genetic variance at 9 years, independent of genetic variance shared across all three ages. NC mathematics at 7, 9, and 10 years also suggests genetic continuity and change in almost equal measure. NC science at 9 and 10 years, however, suggests more change than continuity, as do the reading tests at 7 years (word recognition) and 10 years (reading comprehension). “*g*,” however, shows as much genetic continuity as change.

The shared environmental (C) results in Figure 8 can be interpreted similarly but with one caveat. The effect of shared environment (that is, shared by the twins) is modest; the average estimate of C for NC composite scores across age is only .14. Because the variance of C is modest, attempts to decompose its covariance across age entail large confidence intervals as indicated in Figure 8. Despite this limitation, the general picture that emerges for C in Figure 8 is similar to A: C contributes to continuity and change. For example, the total C contribution to the variance of English at 10 years is .20 (.10+.04+.06) in this longitudinal analysis, which is the same estimate as in our cross-sectional analysis (Table 11). Half (.10÷.20) of the C variance at 10 years is shared in common with 7 and 9 years, one-fifth (.04÷.20) of the C variance at 10 years is shared with 9 years independent of 7 years, and one-third (.06÷.20) of the C variance at 10 years is independent of C variance at 7 and 9 years. The C contribution to continuity is a little less for mathematics, for the tests of reading, and for “*g*.” For science, C contributes entirely to continuity (there is no new C variance at 10 years).

The nonshared environmental (E) results in Figure 8 yield a very different result: E contributes entirely to change for all measures. One caution, however, is that estimates of E include error of measurement which looks like change.

##### Bivariate ACE

The results of the Cholesky model-fitting analyses shown in Figure 8 generally indicate that genetic influences contribute to continuity and change in about equal measure. However, this interpretation focuses on the heritability at each age and the extent to which these genetic influences are shared across ages. Two additional statistics which can be derived from these Cholesky analyses address somewhat different questions, and they indicate even greater genetic stability. As described in Chapter II, bivariate heritability indexes the extent to which the phenotypic correlation between ages is mediated genetically. The rest of the phenotypic correlation is explained by bivariate shared environment and bivariate nonshared environment.

As shown in Table 13, bivariate heritabilities across 7, 9, and 10 for NC English and NC mathematics range from .70 to .79, indicating that genetic factors largely account for the age-to-age phenotypic correlations, which are about .60 to .70. Even from 7 to 10 years, the bivariate heritabilities are .71 for NC English and .78 for NC mathematics. For NC science from 9 to 10 years, bivariate heritability is .54, suggesting that only about half of the phenotypic correlation of .52 from 9 to 10 years is genetically mediated. Although the reading tests at 7 and 10 years showed only modest phenotypic stability (.44) and significant differences in heritability, 83% of their stability can be attributed to genetic mediation. The longitudinal correlations for “*g*” are also largely mediated genetically. Because bivariate heritabilities indicate that genetic influence largely accounts for phenotypic stability, C and E bivariate stability estimates must be smaller, and Table 13 shows that they are.

**TABLE 13:** LONGITUDINAL ANALYSIS: PROPORTION OF PHENOTYPIC CORRELATION (*r*_P_) BETWEEN AGES MEDIATED BY A (*a*_*x*_*a*_*y*_*r*_A_*/r*_P_), C (*c*_*x*_*c*_*y*_*r*_C_*/r*_P_), AND E (*e*_*x*_*e*_*y*_*r*_E_*/r*_P_), AND A (*r*_A_), C (*r*_C_), AND E (*r*_E_) CORRELATIONS (95% CIS IN PARENTHESES)

	A (*a*_*x*_*a*_*y*_*r*_A_*/r*_P_)	C (*c*_*x*_*c*_*y*_*r*_C_*/r*_P_)	E (*e*_*x*_*e*_*y*_*r*_E_*/r*_P_)	*r*_A_	*r*_C_	*r*_E_
NC English: 7, 9, 10						
1. English at 7	*Biv a*^*2*^*1–2*: .79 (.65–.95)	*Biv c*^*2*^*1–2*: .12 (.00–.25)	*Biv e*^*2*^*1–2*: .09 (.06–.12)	*r*_A1_–*r*_A2_: .70 (.61–.79)	*r*_C1_–*r*_C2_: .54 (.00–.91)	*r*_E1_–*r*_E2_: .25 (.16–.34)
2. English at 9	*Biv a*^*2*^*1–3*: .71 (.57–.86)	*Biv c*^*2*^*1–3*: .20 (.06–.33)	*Biv e*^*2*^*1–3*: .09 (.06–.13)	*r*_A1_–*r*_A3_: .67 (.58–.77)	*r*_C1_–*r*_C3_: .71 (.32–1.00)	*r*_E1_–*r*_E3_: .26 (.17–.34)
3. English at 10	*Biv a*^*2*^*2–3*: .70 (.56–.85)	*Biv c*^*2*^*2–3*: .19 (.05–.31)	*Biv e*^*2*^*2–3*: .11 (.08–.15)	*r*_A2_–*r*_A3_: .73 (.64–.81)	*r*_C2_–*r*_C3_: .74 (.36–1.00)	*r*_E*2*_–*r*_E3_: .33 (.24–.41)
NC mathematics: 7, 9, 10						
1. Mathematics at 7	*Biv a*^*2*^*1–2*: .76 (.59–.93)	*Biv c*^*2*^*1–2*: .17 (.01–.32)	*Biv e*^*2*^*1–2*: .07 (.03–.12)	*r*_A1_–*r*_A2_: .62 (.53–.73)	*r*_C1_–*r*_C2_: .80 (.10–1.00)	*r*_E1_–*r*_E2_: .16 (.07–.25)
2. Mathematics at 9	*Biv a*^*2*^*1–3*: .78 (.61–.96)	*Biv c*^*2*^*1–3*: 13 (.00–.28)	*Biv e*^*2*^*1–3*: .09 (.05–.14)	*r*_A1_–*r*_A3_: .68 (.56–.79)	*r*_C1_–*r*_C3_: .52 (.00–.95)	*r*_E1_–*r*_E3_: .20 (.11–.28)
3. Mathematics at 10	*Biv a*^*2*^*2–3*: .75 (.59–.92)	*Biv c*^*2*^*2–3*: .13 (.00–.27)	*Biv e*^*2*^*2–3*: .12 (.08–.16)	*r*_A2_–*r*_A3_: .73 (.63–.84)	*r*_C2_–*r*_C3_: .55 (.00–.96)	*r*_E2_–*r*_E3_: .27 (.19–.36)
NC science: 9, 10						
1. Science at 9	*Biv a*^*2*^*1–2*: .54 (.39–.71)	*Biv c*^*2*^*1–2*: .36 (.21–.49)	*Biv e*^*2*^*1–2*: .10 (.06–.14)	*r*_A1_–*r*_A2_: .49 (.39–.61)	*r*_C1_–*r*_C2_: 1.00 (.69–1.00)	*r*_E1_–*r*_E2_: .19 (.11–.27)
2. Science at 10						
Reading: 7, 10						
1. Reading at 7	*Biv a*^*2*^*1–2*: .83 (.68–.99)	*Biv c*^*2*^*1–2*: .11 (.00–.25)	*Biv e*^*2*^*1–2*: .06 (.02–.10)	*r*_A1_–*r*_A2_: .60 (.51–.71)	*r*_C1_–*r*_C2_: .45 (.00–1.00)	*r*_E1_–*r*_E2_: .11 (.04–.17)
2. Reading at 10						
*g*: 7, 9, 10						
1. *g* at 7	*Biv a*^*2*^*1–2*: .71 (.53–.89)	*Biv c*^*2*^*1–2*: .27 (.11–.42)	*Biv e*^*2*^*1–2*: .02 (.00–.07)	*r*_A1_–*r*_A2_: .70 (.54–.87)	*r*_C1_–*r*_C2_: .37 (.16–.57)	*r*_E1_–*r*_E2_: .03 (.00–.10)
2. *g* at 9	*Biv a*^*2*^*1–3*: .80 (.61–1.00)	*Biv c*^*2*^*1–3*: .18 (.00–.34)	*Biv e*^*2*^*1–3*: .02 (.00–.08)	*r*_A1_–*r*_A3_: .72 (.57–.90)	*r*_C1_–*r*_C3_: .30 (.01–.54)	*r*_E1_–*r*_E3_: .03 (.00–.10)
3. *g* at 10	*Biv a*^*2*^*2–3*: .55 (.42–.69)	*Biv c*^*2*^*2–3*: .38 (.26–.49)	*Biv e*^*2*^*2–3*: .07 (.04–.11)	*r*_A*2*_–*r*_A3_: .74 (.60–.89)	*r*_C2_–*r*_C3_: .66 (.50−.82)	*r*_E2_–*r*_E3_: .15 (.08–.22)

*Note*.—CI, confidence interval.

Why do the Cholesky analyses shown in Figure 8 suggest genetic continuity and change in equal amounts, whereas the bivariate heritabilities shown in Table 13 suggest substantial genetic stability? There can be no conflict here because the bivariate heritabilities are derived directly from the Cholesky analyses, as illustrated below. As mentioned earlier, these analyses focus on different issues. The Cholesky analysis of genetic influence (first part of Figure 8) is focused exclusively on genetic variance (heritability) in the sense that it refers to the extent to which heritability at one age is shared with heritability at another age. In contrast, bivariate heritability focuses on the phenotypic correlation and the extent to which it is mediated by genetic factors.

This contrast can be seen more clearly if we work out an example of the A, C, and E contributions to the phenotypic correlation as derived from the Cholesky analysis in Figure 8 by the chain of paths connecting ages. For example, for English at 7 and 9 years, the A contribution to the phenotypic correlation is .46, as estimated by the product of the path coefficients (√.67 ×√.32=.46). The C contribution is .07 (√.14 ×√.04=.07). The E contribution is .04 (√.19 ×√.01=.04). These A, C, and E contributions sum to .57 which is reasonably similar to the phenotypic correlation of .63 estimated for all individuals without taking into account the paired (twin) structure of the data. Bivariate heritability, the genetic contribution to the phenotypic correlation, is .81 (.46÷.57=.81), which is also similar to the bivariate heritability estimate of .79 shown in Table 13. In other words, 81% of the phenotypic correlation is genetically mediated. In contrast, the Cholesky analysis described above indicates that, of the genetic variance at 9 years (.32+.33=.65), only 49% is shared with genetic variance at 7 years (.32÷.65=.49).

##### ACE Correlations

The second statistic that aids interpretation of longitudinal or multivariate genetic results is the genetic correlation. Bivariate heritability indexes the genetic contribution to the phenotypic correlation; the genetic correlation represents the extent to which genetic influences at one age correlate with genetic influences at the other age regardless of their heritability. Genetic correlations are particularly useful in relation to molecular genetics because they can be thought about as the probability that a gene associated with one trait or age will also be associated with the other trait or age. Shared environment correlations and nonshared environment correlations can be conceptualized similarly, and the full set of correlations are called ACE correlations (see Chapter II for details).

A, C, and E correlations are shown in Table 13. These correlations can be derived from the bivariate ACE estimates. For example, the genetic correlation between NC English at 7 and 9 years is shown in Table 13 as .70, which was obtained from the model-fitting analysis. The origin of this genetic correlation can be seen in Figure 8. The genetic contribution to the correlation between NC English at 7 and 9 years is .46, the product of √.67 and √.32. From the basic multivariate genetic model, this genetic contribution to the phenotypic correlation can be shown to be equivalent to the product of the square roots of the heritabilities of the two variables and their genetic correlation (Plomin & DeFries, 1979). Knowing the heritabilities of the two variables, we can solve for the genetic correlation simply by dividing by the product of the square roots of the heritabilities. For example, the heritabilities for NC English are .67 at 7 years and .65 at 9 years. Dividing .46 (the genetic contribution to the phenotypic correlation) by the product of the square roots of their heritabilities estimates the genetic correlation as .70 [.46÷(√.67 ×√.65)=.70], which is exactly the same as the model-fitting estimate of the genetic correlation shown in Table 13.

The genetic correlations in Table 13 are substantial. Even between 7 and 10 years, the genetic correlations are .67 for NC English, .68 for NC mathematics, and .72 for “*g*.” Even TOWRE at 7 years and PIAT at 10 years yield a high genetic correlation of .60 despite their significant differences in heritability and their modest phenotypic correlation. In what is becoming a familiar pattern, NC science is the odd one out; in this case, it shows the lowest genetic correlation in Table 13, indicating greater qualitative differences in genetic influences from 9 to 10 years.

Unlike bivariate ACE estimates which sum to the phenotypic correlation, ACE correlations are independent; they could all be zero or they could all be unity. In Table 13, the C correlations are in fact quite similar to the A correlations for NC English and mathematics: Between 7 and 10 years, the C correlations are .71 for NC English and .52 for NC mathematics. For “*g*” between 7 and 10 years, the C correlation is more modest, 30. NC science yields the highest C correlation: 1.0 between 9 and 10 years. TOWRE reading at 7 years and PIAT reading at 10 years yield a C correlation of .45. The significant but modest E correlations for all measures (except “*g*”) suggest that nonshared environment is not completely due to error of measurement, which would not be expected to be stable longitudinally.

##### Longitudinal DF Extremes Analysis

In the previous chapter, DF extremes analyses indicated that the abnormal is normal. As described in Chapter II and illustrated in Chapter IV, univariate DF extremes analysis begins with probands selected for extreme scores (or diagnoses) and analyzes how similar the mean of their co-twins is to the mean of the probands on a quantitative measure. By comparing MZ and DZ co-twin means, “group” heritability can be estimated indicating the extent to which the mean quantitative trait score difference between the probands and the population can be attributed to genetic influence.

Univariate DF extremes analysis can be extended to bivariate analysis (Light & DeFries, 1995; Plomin & Kovas, 2005). Although not discussed previously, the same considerations apply to the application of DF extremes analysis to longitudinal data—rather than analyzing two traits at the same measurement occasion, we can analyze the same trait at two measurement occasions. Longitudinal DF extremes analysis can address the issue of genetic mediation of continuity and change for learning disabilities rather than for learning abilities—that is, for the low-performing extremes rather than for individual differences throughout the entire distribution. This form of analysis is especially relevant for the goal of understanding both the antecedents and the long-term consequences of early disability, an issue with theoretical and applied significance.

A brief description of bivariate DF extremes analysis follows. In contrast to univariate DF extremes analysis which selects probands as extreme on *X* and compares the quantitative scores of their MZ and DZ co-twins on *X*, bivariate DF extremes analysis selects probands on *X* and compares the quantitative scores of their co-twins on *Y*, a cross-trait twin group correlation. The genetic contribution to the phenotypic difference between the means of the probands on trait *X* and the population on *Y* can be estimated by doubling the difference between the cross-trait twin group correlations for MZ and DZ twins. *Bivariate group heritability* is the ratio between this genetic estimate and the phenotypic difference between the probands on trait *X* and the population on *Y*. Unlike bivariate analysis of individual differences in unselected samples, such as those done earlier in this chapter, bivariate DF extremes analysis is directional in the sense that selecting probands on *X* and examining quantitative scores of co-twins on *Y* could yield different results as compared with selecting probands on *Y* and examining quantitative scores of co-twins on *X*. A group genetic correlation can be derived from four group parameter estimates: bivariate group heritability estimated by selecting probands for *X* and assessing co-twins on *Y*, bivariate group heritability estimated by selecting probands for *Y* and assessing co-twins on *X*, and univariate group heritability estimates for *X* and for *Y* (see Knopik, Alarcón, & DeFries, 1997). The group genetic correlation is the most informative summative index of genetic effects on low extremes in a longitudinal context. Although it is possible to conduct similar analyses of environmental influences, in this example of a longitudinal DF extremes analysis we will focus on genetic influences.

As an example of bivariate DF extremes analysis applied for the first time to longitudinal data, we analyzed the relationship between 7-year TOWRE scores and 10-year PIAT scores. Because bivariate DF extremes analyses are bidirectional, we conducted two separate analyses: (1) selecting children in the lowest 15% of 7-year TOWRE and analyzing their co-twins' scores on the 10-year PIAT (TOWRE → PIAT), and (2) selecting children in the lowest 15% of 10-year PIAT and analyzing their co-twins' scores on the 7-year TOWRE (PIAT→TOWRE).

For the TOWRE→PIAT longitudinal analysis, the phenotypic group correlation was .58, indicating that children with low scores on the TOWRE at 7 also had low scores on the PIAT at 10–they were 1.5 *SD* below the mean on the TOWRE at 7 and .87 *SD* below the mean on the PIAT at 10. Bivariate group heritability was .61; that is, most of the phenotypic group correlation between the TOWRE and PIAT is mediated genetically. In other words, genetic factors explain 61% of the difference between the mean TOWRE score of probands at 7 years and the population mean on the PIAT at 10 years. That is, to the extent the low scores (lowest 15%) on the TOWRE predict below average scores on the PIAT 3 years later, this is due in large part to common genetic factors.

As noted earlier, the results of bi-directional bivariate extremes analyses need not be symmetrical, as is the case for the analysis of PIAT→TOWRE. The phenotypic group correlation was .39 and bivariate group heritability was only .37, which indicates that genetic factors explain 37% of the difference between the mean PIAT score of probands at 10 years and the population mean on the TOWRE at 7 years. Nonetheless, combining the results for the TOWRE→PIAT and the PIAT→TOWRE analyses yielded a genetic correlation of .90. This suggests that despite the differences in bivariate group heritability for the two analyses, the genetic correlation is substantial between the extremes of TOWRE at 7 years and PIAT at 10 years.

Table 14 compares these results from our longitudinal analyses at the extreme to those presented earlier in this chapter for the entire distribution. Although it is noteworthy that there is lower bivariate heritability at the extremes than across the full distribution, the key result is the genetic correlation, which provides the strongest evidence for genetic links between low scores on the TOWRE at 7 years and low scores on the PIAT at 10 years. Because the TOWRE and PIAT are so different (word recognition versus reading comprehension), this analysis could be viewed as a multivariate analysis as well as a longitudinal analysis. Indeed, it could be argued that all longitudinal analyses are also multivariate analyses because it cannot be assumed that the “same” measure assessed at two ages involves the same cognitive processes at the two ages. In the next chapter, which focuses on multivariate genetic analysis, we include an example of a bivariate extremes analysis between poor performance on reading and on mathematics that is clearly bivariate and not longitudinal.

**TABLE 14:** COMPARISON OF GENETIC RESULTS FROM LONGITUDINAL GENETIC ANALYSES OF THE TOWRE AT 7 YEARS AND PIAT AT 10 YEARS FOR THE ENTIRE DISTRIBUTION (INDIVIDUAL DIFFERENCES) VERSUS THE LOWEST 15% EXTREMES

		DF extremes	
	Individual differences	TOWRE→PIAT	PIAT→TOWRE
Phenotypic correlation	.44	.58	.39
Bivariate heritability	.83	.61	.37
Genetic correlation	.60	.90	

#### SUMMARY

##### Genetic Stability

These developmental analyses lead to the conclusion that genetic influences on learning abilities and disabilities primarily contribute to stability. Genetic correlations from age to age are substantial, about .70, even from 7 to 10 years for NC English, NC mathematics, and “*g*.” Even TOWRE at 7 years and PIAT at 10 years yield a substantial genetic correlation of .60 despite the differences in these measures. In the first application of bivariate DF extremes analysis to longitudinal data, we showed that an even higher genetic correlation of .90 was obtained for the low extremes of the TOWRE and PIAT.

These substantial genetic correlations suggest that genes found to be associated with learning abilities and disabilities at one age are also likely to yield associations at other ages. However, the fact that the genetic correlations are not unity indicates that some gene associations will differ from age to age. Molecular genetic studies that assess learning abilities and disabilities longitudinally are needed to detect all of the genes responsible for their substantial heritability.

ACE correlations are useful in understanding the nature of genetic stability and change regardless of their contribution to phenotypic variance. Bivariate ACE estimates are useful in the more practical sense of understanding genetic and environmental contributions to phenotypic stability and change from age to age. These statistics also indicate genetic stability and environmental change. For NC English, NC mathematics, TOWRE and PIAT reading, and “*g*” from 7 years to 10 years, bivariate heritabilities are .71, .78, .83, and .80, respectively. In other words, about 80% of the phenotypic correlations from 7 to 10 years are mediated genetically.

We know very little about the mechanisms by which genes have their effects on individual differences on cognition and we know even less about how genes affect change and continuity. Many hypothetical mechanisms can be proposed. For example, any DNA variation that contributes to the whole brain efficiency (e.g., myelination) would continue to have its effects across ages. However, until the actual polymorphisms are discovered, these hypotheses will remain speculative.

##### Environmental Change

In contrast to genetic stability, environmental influences, especially nonshared environment, involve change. Shared environment correlations between 7 and 10 years are also high for NC English (.71) but lower for NC mathematics (.52) and for TOWRE and PIAT reading (.45), and much lower for “*g*” (.30). It is not difficult to think of shared environmental factors such as socioeconomic status or school quality that might make twins growing up in the same family and attending the same schools stable longitudinally in their learning abilities. However, these results suggest that shared environmental factors are as much involved in change as continuity; shared environmental factors that make change in a similar way such as changing neighborhoods or schools. However, it should be noted that shared environment accounts for only a modest amount of variance, .14 on average for NC composite scores.

Nonshared environment correlations are lower still: .26, .20, .11, and .03, respectively. Although progress has been slow in identifying specific nonshared environmental factors that make twins different from one another, whatever these factors may be they also largely change from age to age. The remaining 20% of the phenotypic correlation is explained primarily by shared environment.

ACE correlations and bivariate ACE estimates are based on longitudinal analyses of qualitative age changes using multivariate genetic models. The chapter began with an analysis of quantitative age differences in ACE parameter estimates at each age. These analyses indicate stability of both genetic and environmental influences in that ACE parameter estimates are remarkably similar at 7, 9, and 10 years. However, such quantitative age comparisons are much less informative than analyses of qualitative age changes.

The following chapter uses the same multivariate genetic techniques to investigate the causes of relationships within components of learning abilities and between different domains at each age.

#### VI. GENERALIST GENES, SPECIALIST ENVIRONMENTS

The previous chapter moved beyond the basic nature–nurture question by investigating the causes of change and continuity in learning abilities. It began with a brief section on quantitative differences in which genetic and environmental parameter estimates were compared across age. However, its focus was on the etiology of continuity and change, which was addressed by the application of multivariate genetic analysis to longitudinal data. The present chapter uses the same multivariate genetic techniques to investigate common and unique causes within and between learning abilities at each age. For example, to what extent do the genes that affect one aspect of reading ability also affect others? To what extent do genes that affect reading ability also affect mathematics? To what extent do genes that affect reading and mathematics also affect general cognitive ability?

As in the two previous chapters, we have maximized power by combining boys and girls, same-sex and opposite-sex DZ twins, and assessments by same and different teachers. Also, as in the previous chapter, the present chapter primarily reports analyses on individual differences for the entire sample, again for reasons of power. However, similar to the previous chapter, we present an example of a multivariate extremes analysis, in this case for web-based assessments of reading and mathematics at 10 years.

##### PHENOTYPIC CORRELATIONS

If there were no phenotypic correlation between traits, no multivariate genetic analysis would be needed to conclude that different genetic and environmental factors affect the two traits. If traits are correlated phenotypically, as found in decades of research on cognitive and academic abilities, multivariate genetic analysis is essential to determine the extent to which their phenotypic correlation is mediated genetically or environmentally.

Table 15 lists phenotypic correlations within domains at each age. (Appendix E is a complete intercorrelation matrix within and between ages.) The average correlation for the three components within each domain across ages is .69 for NC English, .85 for NC mathematics, and .85 for NC science. These substantial intercorrelations were not limited to teacher NC ratings. Despite the different cognitive processes assumed to underlie reading words and nonwords, these two components on the TOWRE test at 7 years correlate .83. The average correlation between the three components of our web-based battery of mathematics at 10 years was somewhat lower, .59. Even when very different methods were employed, phenotypic correlations could be substantial: At 7 years, NC reading correlated .67 with the TOWRE composite. The correlations between NC ratings and our web-based tests at 10 years were less substantial: .44 for reading and .49 for mathematics.

**TABLE 15:** PHENOTYPIC CORRELATIONS WITHIN DOMAIN AT EACH AGE FOR ONE RANDOMLY SELECTED MEMBER OF EACH TWIN PAIR

	1	2	3
7-year NC English:			
1. Speaking	1		
2. Reading	.64**	1	
3. Writing	.56**	.66**	1
7-year NC mathematics:			
1. Using	1		
2. Numbers	.81**	1	
3. Shapes	.80**	.84**	1
9-years NC English:			
1. Speaking	1		
2. Reading	.69**	1	
3. Writing	.67**	.77**	1
9-year NC mathematics:			
1. Using	1		
2. Numbers	.87**	1	
3. Shapes	.84**	.86**	1
9-year NC science:			
1. Enquiry	1		
2. Life	.80**	1	
3. Physical	.83**	.88**	1
10-year NC English:			
1. Speaking	1		
2. Reading	.71**	1	
3. Writing	.71**	.77**	1
10-year NC mathematics:			
1. Using	1		
2. Numbers	.88**	1	
3. Shapes	.87**	.90**	1
10-year NC science:			
1. Enquiry	1		
2. Life	.83**	1	
3. Physical	.85**	.91**	1
7-year TOWRE			
1. Word	1		—
2. Nonword	.83**	1	—
1. 7-year TOWRE composite	1		—
2. 7-year NC reading	.67**	1	—
1. 10-year PIAT	1		—
2. 10-year NC reading	.44**	1	—
10-year web mathematics:			
1. Understanding Number	1		
2. Nonnumerical Processes	.61**	1	
3. Computation and Knowledge	.64**	.51**	1
1. 10-year web mathematics	1		—
2. 10-year NC mathematics	.49**	1	—

*Note*.—NC, National Curriculum; TOWRE, Test of Word Reading Efficiency; PIAT, Peabody Individual Achievement Test.

** Indicates significance at .01 alpha level.

Correlations were also substantial between domains, as shown in Table 16. The average correlation across the NC composites for the three ages was .75. Web-based tests of reading and mathematics at 10 years correlated .50.

**TABLE 16:** PHENOTYPIC CORRELATIONS BETWEEN DOMAINS AT EACH AGE FOR ONE RANDOMLY SELECTED MEMBER OF EACH TWIN PAIR

	1	2	3	4
7 years				
1. *g*	1			—
2. NC English	.41**	1		—
3. NC mathematics	.39^**^	.74**	1	—
9 years				
1. *g*	1			
2. NC English	.40**	1		
3. NC mathematics	.41**	.74**	1	
4. NC science	.37**	.74**	.75**	1
10 years				
1. *g*	1			
2. NC English	.41**	1		
3. NC mathematics	.38**	.76**	1	
4. NC science	.37**	.78**	.77**	1
7 years				
1. *g*	1		—	—
2. TOWRE	.41**	1	—	—
10 years				
1. *g*	1			—
2. PIAT	.55**	1		—
3. web mathematics	.61**	.50**	1	—

*Note*.—NC, National Curriculum; TOWRE, Test of Word Reading Efficiency; PIAT, Peabody Individual Achievement Test.

** Indicates significance at .01 alpha level.

Also shown in Table 16 are correlations with “*g*”: On average across the ages, “*g*” correlated .41 with NC English, .39 with NC mathematics, and .37 with NC science. With the other test scores, “*g*” correlated .41 with 7-year TOWRE, .55 with 10-year PIAT, and .61 with 10-year mathematics.

In summary, there are substantial phenotypic correlations within and between these domains. In this chapter, we use multivariate genetic analysis to assess genetic and environmental mediation of these phenotypic correlations.

##### MULTIVARIATE GENETIC MODEL-FITTING ANALYSIS

As described in the previous chapter in relation to longitudinal analysis and more generally in Chapter II, multivariate genetics can be used to analyze the covariance between traits rather than the variance of each trait separately in order to estimate the extent to which the same (common) or different (unique) genetic and environmental factors affect the traits. We will not repeat here the description of the two basic sets of statistics of multivariate genetic analysis: bivariate ACE statistics that specify the extent to which the phenotypic correlation is mediated by genetic and environmental factors, and ACE correlations that index the extent to which the same genetic and environmental factors affect the traits regardless of their effect on the phenotype.

The only difference in approach is that the model used in most of the analyses in this chapter is a correlated factors model rather than a Cholesky model. The Cholesky model is most suited to variables that can be ordered, as was the case for the longitudinal analyses presented in the previous chapter in which the variables can be ordered by age. The correlated factors model is more appropriate for most of the multivariate genetic analyses in this chapter because it is not dependent on the order in which the variables are included and it also has the advantage of focusing on ACE correlations. We do use the Cholesky model in the final set of analyses in which we enter “*g*” first in order to investigate the multivariate ACE structure of academic performance in reference to “*g*.” Despite these presentational issues, it should be kept in mind that the correlated factors model and the Cholesky model are algebraic transformations of each other and thus the same bivariate ACE estimates and ACE correlations can be obtained from the two models, as illustrated in the previous chapter.

**FIGURE 9:**
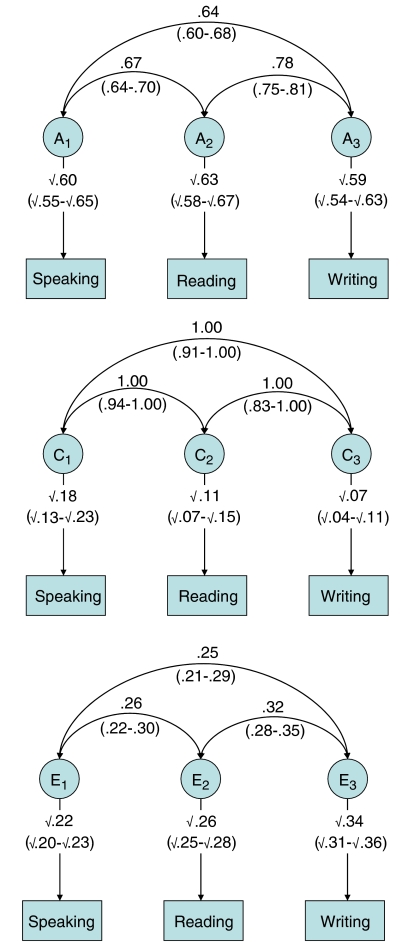
—Trivariate genetic model-fitting results at 7 years for three domains of NC English.

In Figure 9, the three components of NC English at 7 years are used as an example of a correlated factor model-fitting analysis, with results depicted as a path diagram. In the first panel, the coefficients from the latent A variables to the measured traits (shown in rectangles) are heritability estimates (the path coefficient itself is the square root of this heritability). The analogous coefficients in the other panels are shared (C) and nonshared (E) environment contributions to variance. Heritabilities are substantial (61% on average), shared environment is modest (12%) and nonshared environment is moderate (27%). These heritability estimates are similar but not identical to those presented in Chapter III because a univariate full sex-limitation model was used in Chapter III, whereas the present analyses are based on a multivariate model with sexes combined.

As seen in Figure 9, the common factor model directly displays the ACE correlations, which are substantial for A (genetic correlation of .70 on average), unity for C (shared environment correlation of 1.0 on average), and modest for E (nonshared environment correlation of .28 on average). Bivariate heritability is a function of these heritabilities and genetic correlations. For example, multiplying the chain of paths between speaking and reading in the top panel of Figure 9 indicates that the genetic contribution to the phenotypic correlation between speaking and reading is .41 (i.e.,√.60 × .67 ×√.63=.41). The C and E contributions to the phenotypic correlation are .14 and .06. Thus, the model-fitting estimate of the phenotypic correlation is .61 (i.e., .41+.14+.06=.61), which is close to the correlation of .64 shown in Table 15. The bivariate heritability is the proportion of the phenotypic correlation that is mediated genetically, which is 67% (i.e., .41÷.61=.67). The bivariate C and E estimates are 23% and 10%.

##### BIVARIATE ACE ESTIMATES

Rather than showing path diagrams like Figure 9 for all of the multivariate genetic analyses, Table 17 summarizes the bivariate ACE estimates and the ACE correlations within domains. The first row shows a model-fitting estimate of 67% for bivariate heritability for NC speaking and NC reading at 7 years, which is the same as the estimate calculated above. The bivariate ACE estimates indicate that the phenotypic correlations within domains are largely mediated genetically. Bivariate heritabilities are substantial for NC ratings at 7 and 9 years—the average is 72%, meaning that 72% of the phenotypic correlations within domains are mediated genetically. At 10 years, bivariate heritabilities are somewhat (but not nearly significantly) lower—64% on average for English and mathematics and 51% for science. Similar results indicating substantial genetic mediation emerged for Word and Nonword components of the TOWRE at 7 years (73%) and for the three components of the web-based battery of mathematics tests (58%). Bivariate heritabilities were also substantial in comparisons between NC teacher assessments and tests: 83% for NC reading and TOWRE at 7 years, 79% for NC reading and PIAT at 10 years, and 85% for NC mathematics and the composite of the three mathematics components at 10 years.

**TABLE 17:** WITHIN-DOMAIN MULTIVARIATE GENETIC ANALYSES: BIVARIATE GENETIC (A), SHARED ENVIRONMENT (C), AND NONSHARED ENVIRONMENT (E) ESTIMATES AND BIVARIATE A, C, AND E CORRELATIONS (95% CIS IN PARENTHESES)

	A *(a*_*x*_*a*_*y*_*r*_A_*/r*_P_*)*	C *(c*_*x*_*c*_*y*_*r*_C_*/r*_P_*)*	E *(e*_*x*_*e*_*y*_*r*_E_*/r*_P_*)*	*r*_A_	*r*_C_	*r*_E_
7-year NC English						
1. Speaking and listening	*Biv a*^*2*^*1–2:* .67 (.60–.74)	*Biv c*^*2*^*1*–*2:* .23 (.16–.29)	*Biv e*^*2*^*1*–*2:* .10 (.08–.12)	*r*_*A1–*_*r*_*A2*_*:* .67 (.64–.70)	*r*_*C1–*_*r*_*C2*_*:* 1.00 (.94–1.00)	*r*_*E1–*_*r*_*E2*_*:* .26 (.22–.30)
2. Reading	*Biv a*^*2*^*1*–*3:* .67 (.60–.75)	*Biv c*^*2*^*1*–*3:* .21 (.14–.27)	*Biv e*^*2*^*1*–*3:* .12 (.10–.14)	*r*_*A1–*_*r*_*A3*_*:* .64 (.60–.68)	*r*_*C1–*_*r*_*C3*_*:* 1.00 (.91–1.00)	*r*_*E1–*_*r*_*E3*_*:* .25 (.21–.29)
3. Writing	*Biv a*–*3:* .72 (.66–.78)	*Biv c*–*3:* .14 (.09–.19)	*Biv e*–*3:* .14 (.12–.17)	*r*_*A2–*_*r*_*A3*_*:* .78 (.75–.81)	*r*_*C2–*_*r*_*C3*_*:* 1.00 (.83–1.00)	*r*_*E2–*_*r*_*E3*_*:* .32 (.28–.35)
7-year NC mathematics						
1. Using and applying	*Biv a*^*2*^*1*–*2:* .72 (.65–.78)	*Biv c*^*2*^*1*–*2:* .08 (.03–.14)	*Biv e*^*2*^*1*–*2:* .20 (.18–.22)	*r*_*A1–*_*r*_*A2*_*:* .89 (.88–.92)	*r*_*C1–*_*r*_*C2*_*:* 1.00 (.79–1.00)	*r*_*E1–*_*r*_*E2*_*:* .55 (.52–.58)
2. Numbers	*Biv a*^*2*^*1*–*3:* .71 (.64–.78)	*Biv c*^*2*^*1*–*3:* .10 (.04–.16)	*Biv e*^*2*^*1*–*3:* .19 (.17–.21)	*r*_*A1–*_*r*_*A3*_*:* .87 (.85–.89)	*r*_*C1–*_*r*_*C3*_*:* 1.00 (.85–1.00)	*r*_*E1–*_*r*_*E3*_*:* .55 (.52–.58)
3. Shapes	*Biv a*–*3:* .70 (.64–.77)	*Biv c*–*3:* .09 (.03–.15)	*Biv e*–*3:* .21 (.19–.23)	*r*_*A2–*_*r*_*A3*_*:* .91 (.89–.92)	*r*_*C2–*_*r*_*C3*_*:* 1.00 (.89–1.00)	*r*_*E2–*_*r*_*E3*_*:* .62 (.60–.65)
9-Year NC English						
1. Speaking and listening	*Biv a*^*2*^*1*–*2:* .75 (.64–.85)	*Biv c*^*2*^*1*–*2:* .11 (.01–.21)	*Biv e*^*2*^*1*–*2:* .14 (.12–.17)	*r*_*A1–*_*r*_*A2*_*:* .81 (.76–.87)	*r*_*C1–*_*r*_*C2*_*:* .94 (.39–1.00)	*r*_*E1–*_*r*_*E2*_*:* .36 (.31–.42)
2. Reading	*Biv a*^*2*^*1*–*3:* .72 (.62–.83)	*Biv c*^*2*^*1*–*3:* .13 (.04–.22)	*Biv e*^*2*^*1*–*3:* .15 (.12–.18)	*r*_*A1–*_*r*_*A3*_*:* .78 (.73–.83)	*r*_*C1–*_*r*_*C3*_*:* .99 (.72–1.00)	*r*_*E1–*_*r*_*E3*_*:* .33 (.28–.39)
3. Writing	*Biv a*–*3:* .80 (.71–.87)	*Biv c*–*3:* .07 (.01–.15)	*Biv e*–*3:* .13 (.11–.16)	*r*_*A2–*_*r*_*A3*_*:* .89 (.86–.92)	*r*_*C2–*_*r*_*C3*_*:* .98 (.46–1.00)	*r*_*E2–*_*r*_*E3*_*:* .37 (.31–.42)
9-year NC mathematics						
1. Using and applying	*Biv a*^*2*^*1*–*2:* .76 (.67–.82)	*Biv c*^*2*^*1*–*2:* .04 (.00–.12)	*Biv e*^*2*^*1*–*2:* .20 (.17–.23)	*r*_*A1–*_*r*_*A2*_*:* .95 (.94–.98)	*r*_*C1–*_*r*_*C2*_*:* .88 (.00–1.00)	*r*_*E1–*_*r*_*E2*_*:* .62 (.58–.66)
2. Numbers	*Biv a*^*2*^*1*–*3:* .73 (.64–.81)	*Biv c*^*2*^*1–3:* .07 (.00–.16)	*Biv e*^*2*^*1*–*3:* .20 (.18–.23)	*r*_*A1–*_*r*_*A3*_*:* .91 (.89–.94)	*r*_*C1–*_*r*_*C3*_*:* .99 (.65–1.00)	*r*_*E1–*_*r*_*E3*_*:* .62 (.58–.66)
3. Shapes	*Biv a*–*3:* .74 (.64–.82)	*Biv c*–*3:* .06 (.00–.14)	*Biv e*–*3:* .21 (.18–.23)	*r*_*A2–*_*r*_*A3*_*:* .97 (.94–1.00)	*r*_*C2–*_*r*_*C3*_*:* .82 (.00–1.00)	*r*_*E2–*_*r*_*E3*_*:* .65 (.61–.68)
9-year NC science						
1. Scientific enquiry	*Biv a*^*2*^*1*–*2:* .69 (.59–.79)	*Biv c*^*2*^*1*–*2:* .11 (.02–.20)	*Biv e*^*2*^*1*–*2:* .20 (.17–.23)	*r*_*A1–*_*r*_*A2*_*:* .88 (.84–.93)	*r*_*C1–*_*r*_*C2*_*:* .90 (.51–1.00)	*r*_*E1–*_*r*_*E2*_*:* .55 (.50–.59)
2. Life processes	*Biv a*^*2*^*1*–*3:* .67 (.57–.77)	*Biv c*^*2*^*1*–*3:* .11 (.02–.20)	*Biv e*^*2*^*1*–*3:* .22 (.19–.25)	*r*_*A1–*_*r*_*A3*_*:* .89 (.86–.93)	*r*_*C1–*_*r*_*C3*_*:* .97 (.66–1.00)	*r*_*E1–*_*r*_*E3*_*:* .62 (.58–.66)
3. Physical processes	*Biv a*–*3:* .70 (.61–.78)	*Biv c*–*3:* .07 (.00–.16)	*Biv e*–*3:* .23 (.20–.25)	*r*_*A2–*_*r*_*A3*_*:* .93 (.90–.95)	*r*_*C2–*_*r*_*C3*_*:* .90 (.17–1.00)	*r*_*E2–*_*r*_*E3*_*:* .74 (.71–.77)
10–year NC English						
1. Speaking and listening	*Biv a*^*2*^*1*–*2:* .58 (.48–.68)	*Biv c*^*2*^*1*–*2:* .25 (.16–.33)	*Biv e*^*2*^*1*–*2:* .17 (.15–.20)	*r*_*A1–*_*r*_*A2*_*:* .77 (.72–.83)	*r*_*C1–*_*r*_*C2*_*:* .98 (.83–1.00)	*r*_*E1–*_*r*_*E2*_*:* .43 (.38–.48)
2. Reading	*Biv a*^*2*^*1*–*3:* .63 (.54–.73)	*Biv c*^*2*^*1*–*3:* .22 (.12–.30)	*Biv e*^*2*^*1*–*3:* .15 (.12–.18)	*r*_*A1–*_*r*_*A3*_*:* .79 (.75–.83)	*r*_*C1–*_*r*_*C3*_*:* 1.00 (.91–1.00)	*r*_*E1–*_*r*_*E3*_*:* .38 (.32–.43)
3. Writing	*Biv a*–*3:* .61 (.52–.70)	*Biv c*–*3:* .22 (.13–.30)	*Biv e*–*3:* .17 (.15–.20)	*r*_*A2–*_*r*_*A3*_*:* .86 (.82–.92)	*r*_*C2–*_*r*_*C3*_*:* .99 (.86–1.00)	*r*_*E2–*_*r*_*E3*_*:* .45 (.40–.50)
10-year NC mathematics						
1. Using and applying	*Biv a*^*2*^*1*–*2:* .69 (.60–.78)	*Biv c*^*2*^*1*–*2:* .09 (.01–.18)	*Biv e*^*2*^*1*–*2:* .22 (.19–.25)	*r*_*A1–*_*r*_*A2*_*:* .96 (.93–.99)	*r*_*C1–*_*r*_*C2*_*:* .85 (.35–.99)	*r*_*E1–*_*r*_*E2*_*:* .68 (.64–.71)
2. Numbers	*Biv a*^*2*^*1*–*3:* .64 (.55–.74)	*Biv c*^*2*^*1*–*3:* .13 (.04–.21)	*Biv e*^*2*^*1*–*3:* .23 (.20–.26)	*r*_*A1–*_*r*_*A3*_*:* .92 (.90–.95)	*r*_*C1–*_*r*_*C3*_*:* .98 (.81–1.00)	*r*_*E1–*_*r*_*E3*_*:* .70 (.67–.73)
3. Shapes	*Biv a*–*3:* .67 (.58–.76)	*Biv c*–*3:* .11 (.02–.19)	*Biv e*–*3:* .22 (.20–.25)	*r*_*A2–*_*r*_*A3*_*:* .98 (.95–1.00)	*r*_*C2–*_*r*_*C3*_*:* .90 (.62–1.00)	*r*_*E2–*_*r*_*E3*_*:* .71 (.68–.74)
10-year NC science						
1. Scientific enquiry	*Biv a*^*2*^*1*–*2:* .53 (.44–.63)	*Biv c*^*2*^*1*–*2:* .26 (.18–.34)	*Biv e*^*2*^*1*–*2:* .20 (.18–.23)	*r*_*A1–*_*r*_*A2*_*:* .93 (.88–.99)	*r*_*C1–*_*r*_*C2*_*:* .90 (.81–.98)	*r*_*E1–*_*r*_*E2*_*:* .59 (.54–.63)
2. Life processes	*Biv a*^*2*^*1*–*3:* .50 (.42–.60)	*Biv c*^*2*^*1*–*3:* .27 (.19–.35)	*Biv e*^*2*^*1*–*3:* .22 (.20–.25)	*r*_*A1–*_*r*_*A3*_*:* .93 (.88–.98)	*r*_*C1–*_*r*_*C3*_*:* .94 (.86–1.00)	*r*_*E1–*_*r*_*E3*_*:* .65 (.62–.69)
3. Physical processes	*Biv a*–*3:* .49 (.41–.58)	*Biv c*–*3:* .26 (.19–.34)	*Biv e*–*3:* .24 (.22–.27)	*r*_*A2–*_*r*_*A3*_*:* .95 (.92–.98)	*r*_*C2–*_*r*_*C3*_*:* .98 (.94–1.00)	*r*_*E2–*_*r*_*E3*_*:* .78 (.75–.80)
7-year TOWRE						
1. Word	*Biv a*^*2*^*1*–*2:* .73 (.67–.79)	*Biv c*^*2*^*1*–*2:* .16 (.10–.21)	*Biv e*^*2*^*1*–*2:* .11 (.10–.12)	*r*_*A1–*_*r*_*A2*_*:* .88 (.87–.90)	*r*_*C1–*_*r*_*C2*_*:* 1.00 (.90–1.00)	*r*_*E1–*_*r*_*E2*_*:* .50 (.47–.53)
2. Nonword						
7-year reading						
1. TOWRE	*Biv a*^*2*^*1*–*2:* .83 (.75–.91)	*Biv c*^*2*^*1*–*2:* .09 (.01–.17)	*Biv e*^*2*^*1*–*2:* .08 (.06–.10)	*r*_*A1–*_*r*_*A2*_*:* .78 (.73–.82)	*r*_*C1–*_*r*_*C2*_*:* .77 (.23–1.00)	*r*_*E1–*_*r*_*E2*_*:* .28 (.23–.33)
2. NC reading						
10–year web mathematics						
1. Understanding Number	*Biv a*^*2*^*1*–*2:* .57 (.44–.70)	*Biv c*^*2*^*1*–*2:* .26 (.15–.37)	*Biv e*^*2*^*1*–*2:* .17 (.12–.21)	*r*_*A1–*_*r*_*A2*_*:* .95 (.80–1.00)	*r*_*C1–*_*r*_*C2*_*:* .81 (.65–1.00)	*r*_*E2–*_*r*_*E3*_*:* .23 (.18–.29)
2. Nonnumerical Processes	*Biv a*^*2*^*1*–*3:* .59 (.45–.72)	*Biv c*^*2*^*1*–*3:* .18 (.08–.29)	*Biv e*^*2*^*1*–*3:* .23 (.18–.28)	*r*_*A1–*_*r*_*A3*_*:* .90 (.81–.99)	*r*_*C1–*_*r*_*C2*_*:* .95 (.63–1.00)	*r*_*E1–*_*r*_*E3*_*:* .31 (.26–.36)
3. Computation and Knowledge	*Biv a*–*3:* .57 (.42–.74)	*Biv c*–*3:* .24 (.11–.37)	*Biv e*–*3:* .18 (.13–.24)	*r*_*A2–*_*r*_*A3*_*:* .76 (.64–.93)	*r*_*C1–*_*r*_*C3*_*:* .95 (.63–1.00)	*r*_*E2–*_*r*_*E3*_*:* .20 (.14–.25)
10–year reading						
1. PIAT	*Biv a*^*2*^*1*–*2:* .79 (.62–.97)	*Biv c*^*2*^*1*–*2:* .18 (.02–.33)	*Biv e*^*2*^*1*–*2:* .03 (.00–.08)	*r*_*A1–*_*r*_*A2*_*:* .78 (.62–.96)	*r*_*C1–*_*r*_*C2*_*:* .39 (.06–.65)	*r*_*E1–*_*r*_*E2*_*:* .04 (.00–.11)
2. NC reading						
10-year mathematics						
1. Web mathematics	*Biv a*^*2*^*1*–*2:* .85 (.67–1.00)	*Biv c*^*2*^*1*–*2:* .04 (.00–.19)	*Biv e*^*2*^*1*–*2:* .11 (.07–.16)	*r*_*A1–*_*r*_*A2*_*:* .74 (.63–.86)	*r*_*C1–*_*r*_*C2*_*:* .14 (.00–.52)	*r*_*E1–*_*r*_*E2*_*:* .19 (.12–.26)
2. NC mathematics						

*Note*.—NC, National Curriculum; TOWRE, Test of Word Reading Efficiency; PIAT, Peabody Individual Achievement Test.

Bivariate heritabilities were also substantial between domains, as shown in Table 18. The average bivariate heritability across the NC composites for the three ages was 64%. Bivariate heritability was 49% for web-based assessments of reading and mathematics at 10 years. Bivariate heritabilities with “*g*” were 76% on average for NC English, NC mathematics, and NC science composites at 7, 9, and 10 years. Bivariate heritabilities between “*g*” and test scores were 62% with 7-year TOWRE, 51% with 10-year PIAT, and 59% with 10-year mathematics (see Table 19).

**TABLE 19:** “*g*” VERSUS NC COMPOSITES: BIVARIATE GENETIC (A), SHARED ENVIRONMENT (C), AND NONSHARED ENVIRONMENT (E) ESTIMATES AND BIVARIATE A, C, AND E CORRELATIONS (95% CIS IN PARENTHESES)

	A (*a*_*x*_*a*_*y*_*r*_A_*/r*_P_)	C (*c*_*x*_*c*_*y*_*r*_C_*/r*_P_)	E (*e*_*x*_*e*_*y*_*r*_E_*/r*_P_)	*r*_A_	*r*_C_	*r*_E_
7 years						
1. *g*	*Biv a*^*2*^*1–2:* .73 (.60*–*.87)	*Biv c*^*2*^*1–2:* .21 (.09*–*.32)	*Biv e*^*2*^*1–2:* .06 (.03*–*.10)	*r*_*A1–*_*r*_*A2*_*:* .59 (.50*–*.70)	*r*_*C1–*_*r*_*C2*_*:* .38 (.17*–*.56)	*r*_*E1–*_*r*_*E2*_*:* .10 (.04*–*.15)
2. NC English	*Biv a*^*2*^*1–3:* .73 (.59*–*.88)	*Biv c*^*2*^*1–3:* .19 (.06*–*.31)	*Biv e*^*2*^*1–3:* .08 (.04*–*.12)	*r*_*A1–*_*r*_*A3*_*:* .54 (.45*–*.65)	*r*_*C1–*_*r*_*C3*_*:* .38 (.13*–*.61)	*r*_*E1–*_*r*_*E3*_*:* .10 (.05*–*.16)
3. NC mathematics						
9 years						
1. *g*	*Biv a*^*2*^*1–2:* .68 (.52*–*.86)	*Biv c*^*2*^*1–2:* .27 (.11*–*.41)	*Biv e*^*2*^*1–2:* .05 (.01*–*.10)	*r*_*A1–*_*r*_*A2*_*:* .58 (.45*–*.71)	*r*_*C1–*_*r*_*C2*_*:* .42 (.19*–*.65)	*r*_*E1–*_*r*_*E2*_*:* .09 (.01*–*.16)
2. NC English	*Biv a*^*2*^*1–3:* .70 (.54*–*.86)	*Biv c*^*2*^*1–3:* .22 (.07*–*.36)	*Biv e*^*2*^*1–3:* .08 (.03*–*.13)	*r*_*A1–*_*r*_*A3*_*:* .62 (.49*–*.75)	*r*_*C1–*_*r*_*C3*_*:* .44 (.20*–*.71)	*r*_*E1–*_*r*_*E3*_*:* .13 (.05*–*.20)
3. NC mathematics	*Biv a*^*2*^*1–4:* .63 (.45*–*.81)	*Biv c*^*2*^*1–4:* .32 (.15*–*.47)	*Biv e*^*2*^*1–4:* .06 (.01*–*.11)	*r*_*A1–*_*r*_*A4*_*:* .50 (.37*–*.64)	*r*_*C1–*_*r*_*C4*_*:* .48 (.25*–*.75)	*r*_*E1–*_*r*_*E4*_*:* .09 (.01*–*.16)
4. NC science						
10 years						
1. *g*	*Biv a*^*2*^*1–2:* .83 (.63*–*1.00)	*Biv c*^*2*^*1–2:* .12 (.00*–*.29)	*Biv e*^*2*^*1–2:* .05 (.00*–*.11)	*r*_*A1–*_*r*_*A2*_*:* .65 (.52*–*.80)	*r*_*C1–*_*r*_*C2*_*:* .21 (.00*–*.47)	*r*_*E1–*_*r*_*E2*_*:* .08 (.00*–*.16)
2. NC English	*Biv a*^*2*^*1–3:* .91 (.69*–*1.00)	*Biv c*^*2*^*1–3:* .04 (.00*–*.23)	*Biv e*^*2*^*1–3:* .05 (.00*–*.12)	*r*_*A1–*_*r*_*A3*_*:* .67 (.52*–*.83)	*r*_*C1–*_*r*_*C3*_*:* .08 (.00*–*.40)	*r*_*E1–*_*r*_*E3*_*:* .06 (.00*–*.14)
3. NC mathematics	*Biv a*^*2*^*1–4:* .89 (.66*–*1.00)	*Biv c*^*2*^*1–4:* .08 (.00*–*.27)	*Biv e*^*2*^*1–4:* .04 (.00*–*.11)	*r*_*A1–*_*r*_*A4*_*:* .69 (.53*–*.88)	*r*_*C1–*_*r*_*C4*_*:* .10 (.00*–*.35)	*r*_*E1–*_*r*_*E4*_*:* .04 (.00*–*.12)
4. NC science						

*Note*.—NC, National Curriculum.

**TABLE 18:** BETWEEN-DOMAIN MULTIVARIATE GENETIC ANALYSES: BIVARIATE GENETIC (A), SHARED ENVIRONMENT (C), AND NONSHARED ENVIRONMENT (E) ESTIMATES AND BIVARIATE A, C, AND E CORRELATIONS (95% CIS IN PARENTHESES)

	A (*a*_*x*_*a*_*y*_*r*_A_*/r*_P_)	C (*c*_*x*_*c*_*y*_*r*_C_*/r*_P_)	E (*e*_*x*_*e*_*y*_*r*_E_*/r*_P_)	*r*_A_	*r*_C_	*r*_E_
7-year tests						
1. *g*	*Biv a*^*2*^*1–2:* .62 (.52*–*.73)	*Biv c*^*2*^*1–2:* .30 (.20*–*.39)	*Biv e*^*2*^*1–2:* .08 (.06*–*.11)	*r*_*A1–*_*r*_*A2*_*:* . .47 (.40*–*.55)	*r*_*C1–*_*r*_*C2*_*:* .63 (.45*–*.85)	*r*_*E1–*_*r*_*E2*_*:* .14 (.10*–*.19)
2. TOWRE						
7-year NC						
1. English	*Biv a*^*2*^*1–2:* .69 (.63*–*.75)	*Biv c*^*2*^*1–2:* .20 (.14*–*.26)	*Biv e*^*2*^*1–2:* .11 (.10*–*.13)	*r*_*A1–*_*r*_*A2*_*:* .78 (.76*–*.80)	*r*_*C1–*_*r*_*C2*_*:* 1.00 (.97*–*1.00)	*r*_*E1–*_*r*_*E2*_*:* .40 (.36*–*.43)
2. Mathematics						
9-year NC						
1. English	*Biv a*^*2*^*1–2:* .73 (.64*–*.82)	*Biv c*^*2*^*1–2:* .14 (.06*–*.23)	*Biv e*^*2*^*1–2:* .13 (.11*–*.15)	*r*_*A1–*_*r*_*A2*_*:* .79 (.76*–*.83)	*r*_*C1–*_*r*_*C2*_*:* 1.00 (.81*–*1.00)	*r*_*E1–*_*r*_*E2*_*:* .40 (.35*–*.46)
2. Mathematics	*Biv a*^*2*^*1–3:* .69 (.60*–*.79)	*Biv c*^*2*^*1–3:* .17 (.08*–*.26)	*Biv e*^*2*^*1–3:* .14 (.12*–*.16)	*r*_*A1–*_*r*_*A3*_*:* .78 (.74*–*.83)	*r*_*C1–*_*r*_*C3*_*:* 1.00 (.78*–*1.00)	*r*_*E1–*_*r*_*E3*_*:* .46 (.41*–*.51)
3. Science	*Biv a*^*2*^*2–3:* .72 (.62*–*.81)	*Biv c*^*2*^*2–3:* .15 (.06*–*.23)	*Biv e*^*2*^*2–3:* .14 (.11*–*.16)	*r*_*A2–*_*r*_*A3*_*:* .82 (.79*–*.86)	*r*_*C2–*_*r*_*C3*_*:* 1.00 (.85*–*1.00)	*r*_*E2–*_*r*_*E3*_*:* .41 (.36*–*.46)
10-year tests						
1. *g*	*Biv a*^*2*^*1–2:* .51 (.38*–*.63)	*Biv c*^*2*^*1–2:* .39 (.28*–*.50)	*Biv e*^*2*^*1–2:* .10 (.06*–*.14)	*r*_*A1–*_*r*_*A2*_*:* .63 (.52*–*.76)	*r*_*C1–*_*r*_*C2*_*:* .85 (.68*–*.99)	*r*_*E1–*_*r*_*E2*_*:* .17 (.11*–*.23)
2. PIAT	*Biv a*^*2*^*1–3:* .59 (.48*–*.70)	*Biv c*^*2*^*1–3:* .30 (.20*–*.40)	*Biv e*^*2*^*1–3:* .11 (.08*–*.14)	*r*_*A1–*_*r*_*A3*_*:* .76 (.66*–*.86)	*r*_*C1–*_*r*_*C3*_*:* .84 (.66*–*.99)	*r*_*E1–*_*r*_*E3*_*:* .22 (.16*–*.28
3. Web mathematics	*Biv a*^*2*^*2–3:* .49 (.36*–*.63)	*Biv c*^*2*^*2–3:* .41 (.29*–*.51)	*Biv e*^*2*^*2–3:* .10 (.06*–*.15)	*r*_*A2–*_*r*_*A3*_*:* .52 (.43*–*.64)	*r*_*C2–*_*r*_*C3*_*:* 1.00 (.77*–*1.00)	*r*_*E2–*_*r*_*E3*_*:* .16 (.09*–*.21)
10-year NC						
1. English	*Biv a*^*2*^*1–2:* .60 (.52*–*.69)	*Biv c*^*2*^*1–2:* .26 (.18*–*.33)	*Biv e*^*2*^*1–2:* .14 (.12*–*.16)	*r*_*A1–*_*r*_*A2*_*:* .79 (.75*–*.82)	*r*_*C1–*_*r*_*C2*_*:* 1.00 (.94*–*1.00)	*r*_*E1–*_*r*_*E2*_*:* .45 (.40*–*.50)
2. Mathematics	*Biv a*^*2*^*1–3:* .52 (.44*–*.61)	*Biv c*^*2*^*1–3:* .33 (.25*–*.40)	*Biv e*^*2*^*1–3:* .15 (.13*–*.18)	*r*_*A1–*_*r*_*A3*_*:* .78 (.74*–*.82)	*r*_*C1–*_*r*_*C3*_*:* 1.00 (.95*–*1.00)	*r*_*E1–*_*r*_*E3*_*:* .51 (.46*–*.56)
3. Science	*Biv a*^*2*^*2–3:* .55 (.47*–*.64)	*Biv c*^*2*^*2–3:* .29 (.20*–*.36)	*Biv e*^*2*^*2–3:* .16 (.14*–*.19)	*r*_*A2–*_*r*_*A3*_*:* .80 (.76*–*.84)	*r*_*C2–*_*r*_*C3*_*:* 1.00 (.95*–*1.00)	*r*_*E2–*_*r*_*E3*_*:* .49 (.44*–*.54)

*Note*.—NC, National Curriculum; TOWRE, Test of Word Reading Efficiency; PIAT, Peabody Individual Achievement Test.

In summary, bivariate heritabilities were substantial within and between domains. Both shared and nonshared environment account for significant portions of the remainder of the phenotypic correlations not mediated by genetics. Across all of the comparisons in Tables 16 and 17, the average bivariate estimates were 18% for shared environment and 16% for nonshared environment. Relatively greater bivariate shared environment estimates were found for correlations involving test scores: “*g*” and TOWRE at 7 years (30%), “*g*” and PIAT at 10 years (39%), “*g*” and the mathematics composite at 10 years (30%) and between the PIAT and the mathematics composite at 10 years (41%). This might reflect test-taking skills.

##### ACE CORRELATIONS

Tables 17 and 18 also include ACE correlations. To reiterate, ACE correlations index the extent to which the same genetic and environmental factors affect traits regardless of the magnitude of their effects on the phenotypes. A genetic correlation can be viewed as the probability that a DNA marker found to be associated with one trait will also be associated with the other trait. The first row in Table 17 shows a genetic correlation (*r*_A_) of .67 between NC speaking and reading at 7 years, which is the source of the genetic correlation shown in the path model in Figure 9.

The genetic correlations within all of the domains (Table 17) are strikingly high: The average of the 31 genetic correlations in Table 17 is .86. For NC teacher ratings at all three ages, genetic correlations within the English domain are somewhat higher between reading and writing (.84) than between speaking/listening versus reading or writing (.74), although the main point is that both sets of genetic correlations are exceptionally high. High genetic correlations are found for tests as well as teacher ratings. The word and nonword subtests of the TOWRE at 7 years yielded a genetic correlation of .88, and the average genetic correlation among the mathematics subtests at 10 years was .87. Even when different measurement methods were compared, genetic correlations within domains are high: .78 for NC teacher-rated reading versus PIAT web-based test scores at 10 years and .74 between NC teacher-rated mathematics versus web-based mathematics tests scores at 10 years.

Genetic correlations were also substantial between domains (Table 18). The average genetic correlation across the NC composites for the three ages was .79. The genetic correlation was .52 between web-based assessments of reading and mathematics at 10 years. The genetic correlation between “*g*” and test scores was .47 with 7-year TOWRE, .63 with 10-year PIAT, and .76 with 10-year mathematics.

In summary, genetic correlations were very high within and between domains. Because bivariate ACE estimates sum to 100%, if bivariate genetic estimates are high, as they are in our analyses, bivariate shared (C), and nonshared (E) environmental estimates must be low. In contrast, ACE correlations can all be high or low. As shown in Tables 17 and 18, C correlations are extremely high—near unity both within and between domains—with just two striking exceptions. For NC reading and PIAT at 10 years, the C correlation is only .39, although the C correlation is .77 for NC reading and TOWRE at 7 years. The second exception is NC mathematics and web-based mathematics at 10 years, which yielded a C correlation of .14. It is interesting that these exceptions involve comparisons between NC teacher ratings and web-based tests, suggesting that different shared environmental influences affect NC teacher ratings and web-based tests at 10 years.

E correlations are on average half the magnitude of the genetic correlations. Across Tables 17 and 18, the average E correlation is .42, suggesting that different nonshared environmental factors are at work within and between learning abilities. However, E correlations vary considerably across domains. They are consistently lower for the three components of NC English at all ages (.35 on average) than for NC mathematics (.63) and NC science (.66). Similar to the pattern of results for C correlations, E correlations were extremely low for NC reading versus PIAT at 10 years (.04) and for NC mathematics versus web-based mathematics at 10 years (.19).

##### CHOLESKY ANALYSES OF “*g*” AND NC TEACHER ASSESSMENTS

**FIGURE 10:**
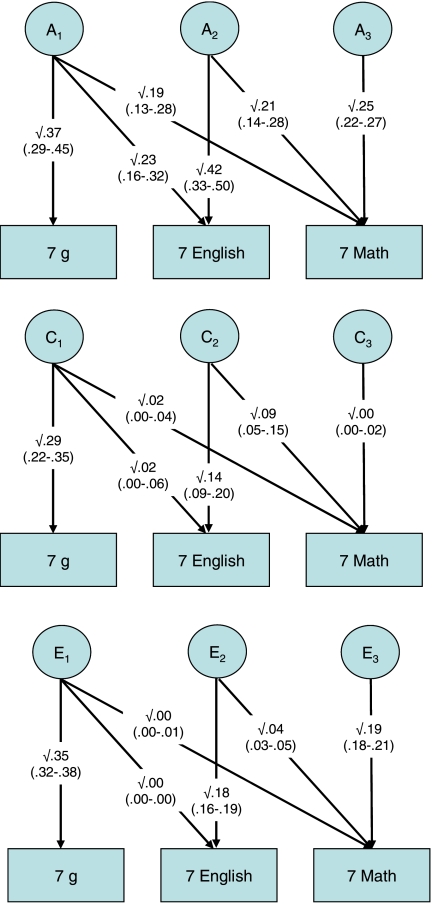
—Seven years: Multivariate genetic model-fitting results for “*g*,” NC English, and NC mathematics (95% confidence intervals shown in parentheses).

**FIGURE 11:**
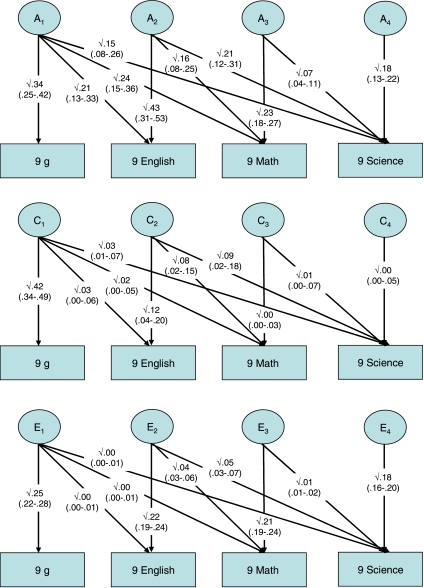
—Nine years: Multivariate genetic model-fitting results for “*g*,” NC English, NC mathematics, and NC science.

**FIGURE 12:**
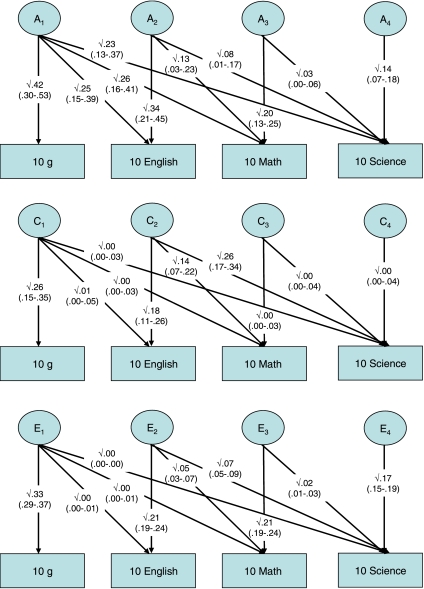
—Ten years: Multivariate genetic model-fitting results for “*g*,” NC English, NC mathematics, and NC science (95% confidence intervals shown in parentheses).

These substantial genetic correlations suggest that the same genes largely affect performance in different academic subjects. Some of these genetic effects are even more general in that they also affect “*g*.” To what extent is it all “*g*”? We incorporated “*g*” and NC ratings of English, mathematics and science in a multivariate genetic analysis that explored the genetic structure of academic performance in relation to “*g*.” As mentioned earlier, a Cholesky model, similar to the model used in the previous chapter, is best suited to address this issue. Separate Cholesky analyses were conducted at 7, 9, and 10 years, with the results shown as path diagrams in Figures 10–12, respectively.

The A_1_ latent variable extracts genetic variance that is in common between “*g*” and academic performance. For example, in Figure 10 (7 years), .37 is the heritability of “*g*.” The A_1_ loadings of .23 for English and .19 for mathematics indicate that a significant and substantial amount of the genetic variance on English and mathematics is shared in common with “*g*.” However, English and mathematics are more highly heritable than “*g*,” as shown in Chapter III. The heritability estimates from the Cholesky model are .65 for English (i.e., .23+.42=.65) and .65 for mathematics (.19+.21+.25=.65). Thus, only a third of the genetic variance on English is shared in common with “*g*” (.23÷.65=.35). Similarly, only a third of the genetic variance on mathematics is shared in common with “*g*” (.19÷.65=.29).

An important feature of the Cholesky model is that it can be used to estimate genetic variance shared by English and mathematics that is independent of “*g*.” This analysis is captured by the A_2_ latent variable. The significant and substantial loadings of English and mathematics on the A_2_ latent variable indicate that English and mathematics share genetic variance independent of “*g*.” For mathematics, about a third of its genetic variance is shared with English independent of “*g*” (.21÷.65=.32). The A_3_ latent variable indexes genetic variance that is unique to mathematics, that is, not shared with either “*g*” or English. Focusing on mathematics, the results suggest that about a third of its genetic variance is in common with both “*g*” and English, about a third is in common with English independent of “*g*,” and the remaining third is unique to mathematics. A similar conclusion would be reached for English if it were the last variable in the Cholesky analysis.

At 9 and 10 years (Figures 11 and 12), NC science is also included in the Cholesky analyses. Focusing on science, which is the last variable in the Cholesky analysis, a similar conclusion emerges at 9 years (Figure 11). The heritability of science at 9 years is estimated in this model as .61 (.15+.21+.07+.18=.61). Of the genetic variance on science, 25% is in common with “*g*,” English, and mathematics (.15÷.61=.25); 34% is independent of “*g*” but in common with English and mathematics (.21÷.61=.34); 11% is independent of “*g*” and English but in common with mathematics; and 30% is unique to science.

At 10 years (Figure 12), the heritability of science is estimated as .48; this lower estimate at 10 years is the same as the model-fitting estimate presented earlier in Table 11. Of this genetic variance, 48% is in common with “*g*,” English, and mathematics; 17% is independent of “*g*” but in common with English and mathematics; 6% is independent of “*g*” and English but in common with mathematics; and 29% is unique to science. This suggests that science at 10 years may have more to do with “*g*” genetically. However, the results for English and mathematics are similar at 10 years in suggesting that only about a third of their genetic variance is shared in common with “*g*.”

The main point of these genetic analyses is that academic performance is not just “*g*.” That is, although about a third of the genetic variance of English and mathematics is in common with “*g*,” about a third of the genetic variance is general to academic performance but not “*g*,” and about a third is specific to each domain.

The results for shared environment suggest that shared environment effects on “*g*” are different from shared environment effects on academic performance. However, the same shared environment factors affect performance in English, mathematics, and science. Nonshared environment is largely unique to “*g*” and unique to each domain of academic performance.

The bivariate ACE estimates and the ACE correlations between “*g*” and NC composites are listed in Table 19 based on the Cholesky model-fitting analyses summarized in Figures 10–12. The bivariate ACE estimates follow directly from the figures: Most (76%) of the phenotypic correlations between “*g*” and NC composites is mediated genetically, and the remainder of the phenotypic correlations are due primarily to shared environment (18%). Nonshared environment accounts for a negligible amount of overlap between “*g*” and NC composites (6%).

The ACE correlations in Table 19 underline the conclusions drawn from the Cholesky analyses. Although the genetic correlations between “*g*” and NC composites are substantial (.61 on average), they are lower, often significantly lower, than the genetic correlations between NC composites, which are about .80 on average (see Table 18). In other words, the general effects of genes on learning abilities is not all “*g*”—learning abilities are more highly correlated genetically with each other than they are with “*g*.” The C correlations between “*g*” and NC composites are moderate (.31 on average) and the E correlations are modest (.08 on average), suggesting again that environmental influences, especially nonshared environmental influences, contribute to differences between learning abilities.

##### MULTIVARIATE DF EXTREMES ANALYSIS

In this chapter, we have focused on multivariate genetic analyses based on individual differences for the entire sample (abilities) rather than extremes (disabilities) for two reasons. First, power is much greater for the entire sample and power is especially critical for multivariate genetic analyses. Second, our univariate analyses of extremes in Chapter III indicate that the results for the extremes are highly similar to results for the entire sample; this is the basis for the conclusion that the abnormal is normal. However, multivariate genetic results could be different for extremes and the entire sample and there are very few examples of multivariate extremes analyses. For these reasons, an example of a bivariate DF extremes analysis is presented in this section, based on two tests administered via the internet at 10 years of age: PIAT reading comprehension and mathematics.

We will not repeat here the description of bivariate DF extremes analysis from the previous chapter, which applied extremes analysis to longitudinal data on reading from 7 years (TOWRE) to 10 years (PIAT). In summary review, bivariate group heritability in our example addresses the genetic contribution to the phenotypic difference between the proband mean on reading and the population mean on mathematics (see Kovas, Haworth, Harlaar, Petrill, Dale, & Plomin, in press). Because bivariate DF extremes analysis is directional, two analyses need to be conducted that could yield different results: Selecting probands for poor reading performance and comparing co-twin quantitative trait scores on mathematics (reading → mathematics) and vice versa (mathematics → reading). From these two analyses, a bivariate extremes genetic correlation can be derived. Similar bivariate extreme estimates can be obtained for shared and nonshared environment but in this example we focus on genetic factors in order to simplify the presentation.

As in previous analyses of extremes, we selected probands for scores in the lowest 15% of reading and mathematics. For the reading→mathematics analysis, the phenotypic group correlation was .60 indicating that children with the lowest reading scores also had low mathematics scores. More specifically, the reading probands had reading scores that were 1.6 SD below the population mean on reading and .96 SD below the population mean on mathematics (−.96÷−1.6=.60). Bivariate group heritability was .38. That is, genetic factors explained 38% of the difference between the mean reading score of probands and the population mean on mathematics.

Results for the mathematics→reading analysis were similar. The phenotypic group correlation was .46 and bivariate group heritability was .24. Although the bivariate group heritabilities at the extremes were lower than the bivariate heritabilities described earlier, combining the results for the reading→mathematics analysis and the mathematics → reading analysis yielded a genetic correlation of .67. That is, two-thirds of the genetic effects on low reading and low mathematics are in common.

Table 20 compares the bivariate extremes results to bivariate results for the entire sample. The results are roughly similar, suggesting general genetic effects that encompass not only reading and mathematics abilities but also disabilities.

**TABLE 20:** COMPARISON BETWEEN BIVARIATE GENETIC RESULTS FOR THE LOW EXTREMES AND FOR THE ENTIRE SAMPLE FOR READING AND MATHEMATICS TEST SCORES AT 10 YEARS

		DF Extremes	
	Individual Differences	PIAT→ Mathematics	Mathematics→ PIAT
Phenotypic correlation	.50	.60	.46
Bivariate heritability	.49	.38	.24
Genetic correlation	.52	.67	

*Note*.—PIAT, Peabody Individual Achievement Test

##### SUMMARY

###### Generalist Genes

These multivariate genetic results are consistent with other research (Plomin & Kovas, 2005) in yielding high genetic correlations within and between learning abilities. Within domains, the average of the 31 genetic correlations reported in Table 17 was .86. High average genetic correlations within domains emerged not just for NC teacher ratings (.87) but also for subtests of the TOWRE (.88) and the mathematics battery (.87). Even across methods (NC teacher ratings of reading and mathematics versus tests of reading and mathematics), genetic correlations within domains were high (.76). Finding such high genetic correlations within domains is striking because most of the components within domains seem to require quite different cognitive processes (e.g., reading words and nonwords in the TOWRE test for which the genetic correlation was .88). As another example, the three mathematics subtests represent three very different aspects of mathematics—straightforward computations, nonnumerical mathematical processes including concepts such as rotational or reflective symmetry, and understanding the numerical and algebraic processes that need to be applied to solve particular problems—and yet their average genetic correlation was .87.

Even more surprising are the high genetic correlations among the NC composites of English, mathematics, and science, where the average genetic correlation at 7, 9, and 10 years was .79. The web-based tests of reading and mathematics at 10 years yielded a genetic correlation of .52. Although the multivariate genetic analyses in this chapter are primarily based on the entire sample, a bivariate extremes analysis yielded results similar to those based on the entire sample. We emphasize genetic correlations because they indicate the extent to which the same genes affect different traits regardless of the heritability of the traits. However, bivariate heritabilities, which specify the extent to which genetic factors mediate the phenotypic correlation between traits, were also substantial. For example, the average bivariate heritability for NC ratings at 7, 9, and 10 years was 67% within domains (Table 17) and 64% between domains (Table 18).

These results lead us to conclude that the same set of genes is largely responsible for genetic influence on these diverse areas of learning abilities and disabilities. In order to highlight this general effect of genes, we refer to them as “generalist genes” (Plomin & Kovas, 2005). When DNA research identifies any of the many genes responsible, for example, for the high heritability of reading ability and disability, we predict that most (but not all) of these genes will also be associated with mathematics ability and disability. The notion of generalist genes has far-reaching implications for diagnosis and treatment of learning disabilities and for understanding the cognitive and brain mechanisms that mediate the effects of generalist genes on behavior. These implications of generalist genes are discussed in the following final chapter.

Our multivariate genetic analyses between learning abilities and general cognitive ability (“*g*”) suggest that some generalist genes that affect learning abilities are even more general in that they also affect other sorts of cognitive abilities included in “*g*.” However, generalist genes are not just “*g*” because learning abilities are more strongly correlated genetically with each other than they are with “*g*.” About a third of the genetic variance of English and mathematics is in common with “*g*,” about a third of the genetic variance is general to academic performance independent of “*g*,” and about a third is specific to each domain. Science at 10 years is more genetically related to “*g*” than are English and mathematics. One possible explanation is that the general environment (TV, newspapers, etc.) may play more of a role in science than in English and mathematics, which are more formally taught. The hallmark of “*g*” is the ability to pick up knowledge from a relatively unstructured environment.

As with longitudinal analyses, the mechanisms through which generalist genes have their effects on covariation between different traits are as yet to be discovered. Existing cognitive theories that attempt to explain the positive manifold among cognitive tasks propose different mechanisms for this phenomenon (see Van der Maas et al., 2006, for review). When the DNA polymorphisms involved in individual differences in each ability are discovered, the generalist genes hypothesis, and its relation to various cognitive theories, can be definitively tested.

###### Specialist Environments

Like the genetic correlations, the shared environmental correlations are very high. Thus, what differentiates learning abilities is largely nonshared environmental factors that make children growing up in the same family different from one another. Nonshared environment correlations are on average about .40, in contrast to the average genetic correlation of about .80. In other words, the nonshared environmental factors that affect one domain are mostly different from those that affect another domain. Bivariate nonshared environment estimates are 16% on average, indicating that nonshared environmental factors do not contribute much to the substantial correlations among learning abilities.

Unlike shared environment, for which it is easy to point to possible influences with general effects such as socioeconomic status or school quality, it is more difficult to imagine nonshared environmental influences that might affect siblings differently—in this case, even clones (MZ twins) growing up in the same family, attending the same schools, and sitting in the same classrooms. Even though we have a long way to go to understand such nonshared environmental influences, we now have another reason to promote research in this area: These influences are the source of specialist environments contributing to perturbations in children's profiles of performance across academic subjects. One implication is that educational programs might have their greatest impact on remediating discrepant performances between learning abilities (such as differences in reading and mathematics). That is, if the environment contributes most to differences in performance in reading and mathematics, it seems reasonable to expect that such performance profile differences might be most susceptible to intervention. The same speculation might apply to discrepancies between learning abilities and cognitive abilities, which is one way to view over-achievement and under-achievement.

We hypothesize that the effects of nonshared environments will be similar to those of genes: There will be many environments, each having only a small effect on a particular phenotype. Finding such influences will be a difficult task and will require innovative methods, such as focusing on perceptions rather than “objective measures” and using genetically sensitive designs, such as studying discordant monozygotic twins. We predict that once specific measured genes and environments are available, this information will be widely utilized to predict and prevent learning disabilities, to evaluate interventions, and to study gene–environment interplay.

##### VII. CONCLUSIONS AND IMPLICATIONS

In this monograph, we have investigated the genetic and environmental origins of individual differences in performance in academic subjects (English, mathematics, and science) and general cognitive ability during the early school years. We began with the basic nature–nurture question about the relative influence of genes and environment (Chapter III). However, our main goal was to address three questions that go beyond this rudimentary question: (1) the etiological relationship between the normal (learning abilities) and the abnormal (learning disabilities), (2) genetic and environmental contributions to longitudinal stability and change from 7 to 10 years, and (3) genetic and environmental heterogeneity and homogeneity within and between learning abilities (English, mathematics, and science) as well as their relationship to general cognitive ability. These three themes were the topics of Chapters IV, V, and VI, respectively. In this final chapter, we begin by discussing our findings in relation to these three themes and then we return to more general issues related to nature and nurture that emerge from the results presented in Chapter III.

###### THE ABNORMAL IS NORMAL

The results presented in Chapter IV lead us to conclude that learning disabilities are the quantitative extreme of the same genetic and environmental influences that operate throughout the normal distribution of learning abilities. Stated more provocatively, these results suggest that there are no learning disabilities, just the low end of the normal distribution of learning abilities. Using reading disability as an example, when the genes responsible for the several replicated linkages are identified (McGrath, Smith, & Pennington, 2006; Fisher & Francks, 2006), we predict that these same genes will be associated with normal variation in reading ability, not just with reading disability. That is, even in pairs of siblings who are both good readers, we would expect that siblings with one or two copies of the “beneficial” allele will be better readers than their co-siblings who only have the other allele. Similarly, both shared and nonshared environmental factors associated with poor reading are also expected to be associated with variation throughout the normal distribution including good reading. This conclusion may appear counterintuitive, given the long tradition in psychology and education of viewing reading disability as a qualitatively distinct category. In fact, there is considerable convergence with an emerging cognitive view of variability in reading that emphasizes a continuum of variability. For example, poor readers have the same difficulties and make the same kinds of errors in reading as average readers, just more of them, and they last longer (cf., Catts & Kamhi, 2005).

Although we presented results using a 15% cutoff for reasons discussed in Chapter IV, we have conducted similar analyses using a 5% cutoff and found similar results. Of course, different results could emerge if different phenotypes were used for selection, such as selecting for a syndrome of multiple traits or selecting for specific learning disability in which children with poor performance are also required to have normal “*g*.” In a study of this latter type comparing specific versus nonspecific language impairment in 4-year-olds in TEDS, some differences appeared although power to detect such differences was modest (Hayiou-Thomas, Oliver, & Plomin, 2005). As indicated in Chapter VI, there is substantial genetic overlap between learning abilities and “*g*,” which suggests that specific language impairment with reduced variance in “*g*” could quite plausibly yield different results. One practical problem is that when selecting probands for a complex phenotype of this sort, it is difficult to know what quantitative trait to use for co-twins in DF extremes analyses.

####### High Ability

Although we have focused on low learning ability because of its educational and societal importance, to what extent is high ability also the quantitative extreme of the same genetic and environmental factors responsible for normal variation in ability? High ability has been a nature–nurture battleground. For example, some theorists have argued that high performance is driven entirely by the time and effort spent developing relevant skills (e.g., Ericsson, Krampe, & Tesch-Romer, 1993; Howe, Davidson, & Sloboda, 1998). Others have argued for the primacy of innate brain-based differences (e.g., Geschwind & Galaburda, 1987). However, very little is actually known about the origins of high academic performance (Plomin & Thompson, 1993). The only genetic studies of this type focused on “*g*” rather than academic performance (e.g., Ronald, Spinath, & Plomin, 2002). In the first genetic study of high mathematics ability (Petrill, Kovas, Hart, Thompson, & Plomin, submitted), TEDS' web-based test data at 10 years yielded results similar to results reported in Chapter IV for the low end of the distribution and for the entire distribution of individual differences (Kovas, Haworth, Petrill, & Plomin, in press): substantial genetic influence, modest shared environmental influence, and moderate nonshared environmental influence.

As with low ability, it would be fallacious to pose the question of the source of high ability as a question of nature versus nurture. The substantial heritability of high ability does not mean that genes simply turn on and cause a child to perform at high levels. Although skills can be taught and high levels of performance can be attained regardless of genetic propensities, even at high levels of performance differences will remain and genetics is likely to play just as large a role at this high end of the distribution. Moreover, nature and nurture are not separate tracks in development. It is clear that high-performing children are more likely to engage in activities such as deliberate practice that enhance their abilities (Ericsson, Krampe, & Tesch-Romer, 1993). The substantial genetic influence at the high end of the distribution suggests that engaging in deliberate practice is in part a function of genes influencing ability indirectly, but powerfully, through motivation. Put more simply, genes code for appetites, not just aptitudes. Such gene–environment transactions are important for understanding why some children fail to benefit fully from enriched environments and why others reach high levels of performance despite environmental privation.

####### Quantitative Trait Loci (QTLs)

A model for understanding why the abnormal is normal is the QTL hypothesis, which suggests that a polygenetic continuum of genetic risk underlies a continuum of variation in behavior in the population and that common disorders lie at the extreme end of this normal variation (see Plomin et al., in press, for more detail). The QTL model refers to quantitative traits even in relation to disorders because if many genes affect a disorder, then it necessarily follows that there will be a quantitative distribution rather than a dichotomy. As with all of our conclusions based on the quantitative genetic research presented in this monograph, definitive proof that the abnormal is normal will come when genes identified for learning disabilities are found to be associated with the normal range of variation in learning abilities and vice versa.

The conclusion that the abnormal is normal is limited to common disorders. For all complex disorders—including medical disorders such as obesity and heart disease as well as behavioral problems such as mental retardation—there are rare, highly penetrant mutations that can create extreme versions of a disorder, which may show qualitative differences from normal variation. For example, contrary to the QTL hypothesis, an apparently unique genetic contribution to language impairment was hailed in the discovery of the FOXP2 mutation in the KE family (Lai, Fisher, Hurst, Vargha-Khadem, & Monaco, 2001). The FOXP2 mutation appears to be both necessary and sufficient for the 15 affected members of the KE family with an unusual type of speech–language impairment that includes deficits in oro-facial motor control. However, the FOXP2 mutation was not found in a single one of 270 children with low language ability in TEDS (Meaburn, Dale, Craig, & Plomin, 2002). More generally, hundreds of rare mutations with effects on “*g*” have been identified (Inlow & Restifo, 2004), but together these mutations appear to account for <1% of cases of mental retardation. We predict that many QTLs of small effect rather than one or two genes of large effect will account for most of the genetic variation in learning disabilities. We also predict that these QTLs will relate to variation in learning ability throughout the normal distribution.

####### Quantitative Trait Neural Processes (QTNs)

If learning disabilities involve many QTLs of small effect, then there are also likely to be many brain mechanisms that mediate the effects of these QTLs on learning disabilities. In other words, learning disabilities may be the extremes of the same brain and cognitive processes that are responsible for normal variation, as opposed to a “broken brain” with one malfunctioning part like a lesion that lights up in neuroimaging studies. We offer the term “qualitative trait neural processes” (QTNs) to highlight the possible parallels with QTLs (Kovas & Plomin, 2006). Both QTNs and QTLs support a shift of thinking about diagnosed abnormal individuals toward thinking about normal variation.

###### GENETIC STABILITY, ENVIRONMENTAL CHANGE

The results of longitudinal genetic analyses presented in Chapter V suggest that age-to-age stability is primarily mediated genetically whereas the environment, especially nonshared environment, contributes to change from age to age. Chapter V began by reporting remarkably similar quantitative ACE estimates at 7, 9, and 10 years of age, even for “*g*” for which the measures were as different as could be at 7 (telephone testing), 9 (mailed booklet), and 10 (web-based testing). It is striking that ACE estimates are so similar across this third of the children's lives despite major changes in their cognitive development and in the content of the measures. Nonetheless, ACE estimates could be similar from age to age even if different ACE factors operated at each age. Longitudinal analyses of the etiology of age-to-age change and continuity are key to understanding the development of individual differences in learning abilities and disabilities.

####### Genetic Stability

As discussed in Chapter V, longitudinal genetic analyses yield two types of genetic statistics: bivariate heritability and genetic correlation. Bivariate heritabilities, which indicate the proportion of the phenotypic correlation from age to age that is mediated genetically, are about .75 on average for NC teacher ratings across 7, 9, and 10 years. The reading tests from 7 to 10 years yield a bivariate heritability of .83. These bivariate heritabilities suggest that age-to-age stability of academic and cognitive abilities is largely mediated genetically.

Genetic correlations estimate the extent to which genetic influences at one age correlate with genetic influences at another age regardless of their heritability—that is, bivariate heritability could be low but genetic correlations could be high. Genetic correlations can be considered as the probability that a gene associated with a trait at one age is also associated with the trait at the other age. The genetic correlations from 7 to 10 years are .67 and .68 for NC teacher ratings for English and mathematics, respectively, .60 for reading tests, and .72 for “*g*.” These high genetic correlations across one-third of the children's lives indicate that genetic effects are largely stable, which is remarkable given the developmental changes during middle childhood. However, because the genetic correlations are <1.0, they also suggest some changes in genetic effects from age to age.

Molecular genetic studies that identify the genes responsible for the high heritability of learning abilities and disabilities will provide the definitive test of this conclusion derived from quantitative genetic analyses. These quantitative genetic analyses predict that the chances are about two-thirds that a gene found to be associated with learning abilities at 7 years would also be associated with learning abilities at 10 years.

Nonetheless, this glass can also be seen as about one-third empty: The chances are about one-third that a gene associated at 7 years would not be associated at 10 years. What about molecular genetic studies with samples of a wide range, as is the case for most genetic studies? Longitudinal analyses typically yield a simplex pattern of correlations in which correlations are lower as age intervals increase. If age-to-age genetic correlations follow this simplex pattern, genetic stability will be less the longer the age interval. For a wide age interval—for example, from childhood to adulthood—age-to-age genetic correlations might be quite low. If this were the case, molecular genetic studies with a wide age range would only be able to detect the most age-general genes. Although it would be important to identify such age-general genes, given the evidence for age changes in genetic effects, many genetic effects across a wide age range would not be age-general and such studies would be unlikely to detect these age-specific genes. Given that genes largely contribute to stability both for ability and disability, longitudinally stable phenotypes—for example, children who have shown low performance for a particular learning ability throughout childhood—seem to be the best targets for molecular genetic studies. The most important benefit of identifying genes that put children at risk for developing learning disabilities is that the genes can be used as an early-warning system to predict problems before they occur. Genes associated with learning problems at 7 years can be used to predict early in life a child's risk for developing learning problems at 7 years. Although most of the genes associated with learning problems at 7 years will also be associated with learning problems at 10 years, the genetic prediction could be sharpened by focusing on those genes that are stably associated with learning problems at 7 and 10 years and beyond. In addition, genes that contribute to change from 7 to 10 years could be used to predict problems that are unlikely to develop until 10 years or transitory problems at 7 years that will be resolved by 10.

The value of early prediction is the opportunity it affords for prevention. Identifying children in early childhood who are genetically at risk for learning problems in middle childhood will encourage research that charts the developmental course of the learning problems and research that intervenes to change the course of development. This goal is achievable even in the case of skills such as reading that do not occur until later in development. Reading is a good example because there is a large and widely accepted body of evidence that phonology—and specifically the ability to reflect on the sound structure of spoken words—lies at the core of reading development and reading problems (Goswami & Bryant, 1990). These issues are discussed more fully later in this chapter.

####### Environmental Change

Because about 75% of phenotypic stability from 7 to 10 years is mediated genetically, it necessarily follows that about 25% is mediated environmentally. Nearly all of this environmental stability is due to shared environment, as indicated by the bivariate shared environment estimates in Table 14. In terms of environmental correlations rather than bivariate environmental estimates, we found that shared environmental correlations from 7 to 10 years are almost as high as the genetic correlations: .71 for NC English, .52 for NC mathematics, and .45 for reading tests, but only .30 for “*g*.” However, nonshared environmental correlations are uniformly low: .26, .20, .11, and .03, respectively. In other words, nonshared environment largely contributes to change.

What are these nonshared environmental sources of change? Nonshared environment, which accounts for more variance than shared environment, is a major mystery for learning abilities and disabilities because the twins live in the same family, attend the same school, and are often even in the same classroom. Nonshared environment is discussed later in this chapter, but for now we simply mention another piece of this puzzle: Not only do nonshared environmental influences on learning abilities and disabilities make two children in the same family different from one another, they also make children at one age different from themselves at another age. The motivation for identifying significant nonshared environmental features should be at least as strong as the motivation for identifying DNA markers because nonshared environment appears to be the major source of change, and change is the essence of education.

###### GENERALIST GENES, SPECIALIST ENVIRONMENTS

Multivariate genetic analyses presented in Chapter VI lead to the conclusion that genes are generalists and nonshared environments are specialists. That is, genes largely contribute to similarity in performance within and between learning abilities, and between learning abilities and general cognitive ability, whereas nonshared environment contributes to differences in performance.

####### Generalist Genes

Within domains, genetic correlations were extraordinarily high: .87 on average for the three components of each domain of NC teacher ratings, .88 for the two subtests of the TOWRE, and .87 for the three components of the mathematics battery. This suggests that the components within each domain are nearly the same thing from a genetic perspective. Even more surprising were the high genetic correlations between domains. The average genetic correlation among NC teacher ratings of English, mathematics, and science at 7, 9, and 10 years was .79. The genetic correlation was .52 between the web-based tests of reading and mathematics at 10 years. Bivariate heritabilities were also substantial: .67 within domains and .64 between domains for NC ratings for the three ages, which indicates that about two-thirds of the phenotypic correlation between these domains is mediated genetically.

Our results are similar to those of other multivariate genetic studies on learning abilities and disabilities, which consistently yield high genetic correlations. For example, the first study in this area using standard measures of reading and mathematics reported a genetic correlation of .98 between reading and mathematics (Thompson, Detterman, & Plomin, 1991). In a recent review, genetic correlations varied from .67 to 1.0 for reading versus language (five studies), from .47 to .98 for reading versus mathematics (three studies), and from .59 to .98 for language versus mathematics (two studies) (Plomin & Kovas, 2005). The average genetic correlation between domains was about .70. We refer to these genetic effects as “generalist genes” in order to highlight the general effect of genes within and between learning abilities and disabilities (Plomin & Kovas, 2005).

We also found that some of these generalist genes that affect learning abilities are even more general in that they also affect other sorts of cognitive abilities included in the “*g*” factor. As reported in Chapter VI, the average genetic correlation between learning abilities and “*g*” is about .60. We argued there that academic performance is not just “*g*.” Although about a third of the genetic variance of English and mathematics is in common with “*g*,” about a third of the genetic variance is general to academic performance but not “*g*.”

Similar to issues discussed above in relation to genetic stability, the fact that genetic correlations are <1.0 means that there are also genes that contribute to predisposing children to perform better in one domain than another. Because genetic influence on learning abilities is substantial, such specialist genes contribute importantly to dissociations among learning abilities even though most genes are generalists.

As mentioned in the previous section, definitive proof of the importance of generalist genes will come from molecular genetic research. The prediction is clear: Most (but not all) genes found to be associated with a particular learning ability or disability (such as reading) will also be associated with other learning abilities and disabilities (such as mathematics). In addition, most (but not all) of these generalist genes for learning abilities (such as reading and mathematics) will also be associated with other cognitive abilities (such as memory and spatial).

When these generalist genes are identified, they will greatly accelerate research on general mechanisms at all levels of analysis from genes to brain to behavior. Implications of generalist genes for cognitive and brain sciences have recently been discussed (Kovas & Plomin, 2006).

Implications of generalist genes for translational research are also far-reaching. The most immediate implication is that, from a genetic perspective, learning disabilities are not distinct diagnostic entities.

####### Specialist Environments

Multivariate genetic research also has an interesting story to tell about environmental influences on learning abilities and disabilities. Shared environmental influences are also generalists: Shared environmental correlations are at least as high as genetic correlations. However, nonshared environmental correlations are on average half the magnitude of the genetic correlations, about .40 on average within and between learning abilities, although they vary considerably across domains. Nonshared environmental correlations between learning abilities and “*g*” are very low, about .10 on average.

We conclude that nonshared environmental influence is largely specific to each learning ability. This adds another piece to the puzzle of nonshared environment. As noted in the previous section, not only do nonshared environmental influences on learning abilities make two children in the same family different from one another, but they also make children at one age different from themselves at another age. Now we add the additional clue that these nonshared environmental influences also make children different across domains of learning. In other words, nonshared environments are specialists. It is difficult to imagine what such nonshared environmental influences might be; we return to this issue in the following section. As mentioned in the previous chapter, one implication of this conclusion is that educational influences might have their greatest impact on remediating discrepant performances among learning abilities such as differences in children's performance in reading and mathematics.

###### LIMITATIONS

General limitations of the twin method and specific limitations of the present study were discussed in Chapter II. Three other limitations may be especially relevant to our finding of substantial heritability and modest shared environment. The first limitation involves the possibility of assortative mating. Assortative mating, which is the correlation between spouses, inflates the DZ twin correlation but does not affect the MZ twin correlation (Plomin et al., in press). Thus, assortative mating could have deflated our heritability estimates and inflated our shared environment estimates, even though the heritabilities are so high and the estimates of shared environment are so low. Assortative mating is substantial in the cognitive domain, about .40 for “*g*” (Jensen, 1978), although assortative mating for academic performance itself is not known. If assortative mating for parents' academic performance were also as high as .40, heritabilities adjusted for assortative mating could be as high as 80% and shared environment could be as low as 0% for learning abilities.

A second limitation that could also have inflated our estimates of shared environment is the possibility that twins share environmental experiences to a greater extent than nontwin siblings because twins are the same age and thus travel through life together. In early childhood, TEDS research indicated that for cognitive abilities, estimates of the role of shared environment were more than twice as large for twins as compared with nontwins siblings, suggesting that about half of twin study estimates of shared environment for cognitive abilities in early childhood are specific to twins (Koeppen-Schomerus, Spinath, & Plomin, 2003). We will be able to assess this possibility for learning abilities in middle childhood because younger siblings of the TEDS twins are also being assessed as they reach middle childhood.

Unlike the previous two limitations, which perversely suggest ways in which our high estimates of heritability could be even higher and our low estimates of shared environment could be even lower, the third limitation could explain why our heritability estimates are so high and our shared environmental estimates are so low. The U.K. National Curriculum provides similar curricula to all students, thus diminishing a potentially important source of environmental variation across schools, to the extent that the curriculum actually provides a potent source of environmental variation. In contrast, the educational system in the United States is one of the most decentralized national systems in the world. To the extent that these differences in educational policy affect children's academic performance, we would expect greater heritability and lower shared environment in the United Kingdom than in the United States. In other words, all other things being equal, greater equality in educational opportunity should lead to greater heritability. Differences in samples, ages, and measures among twin studies of learning abilities and disabilities make it difficult to compare U.K. and U.S. results. In particular, very large samples are needed to provide reasonable power to detect differences in heritability. The only large study other than TEDS is the U.S. study of bright twins in high school (Loehlin & Nichols, 1976). Results for that study are in the direction predicted by the “national curriculum” hypothesis: Heritability is lower than in TEDS' NC ratings (40% vs. 60%) and shared environment is higher (30% vs. 15%). One other U.S. study, with a smaller sample, also reported lower heritability (40%) and higher shared environment (40%) than TEDS (Thompson, Detterman, & Plomin, 1991). However, the national curriculum hypothesis is not supported by the results from studies in the Netherlands (Bartels, Rietveld, van Baal, & Boomsma, 2002b) where there is a national curriculum but one that is less prescriptive than the U.K. curriculum, and in Australia (Wainwright, Wright, Luciano, Geffen, & Martin, 2005) where there is no national curriculum other than for literacy (O'Donnell, 2004). The sample sizes in these other studies are not nearly large enough to provide adequate power to compare results across studies, however. We are currently coordinating our U.K. TEDS study with a U.S. study with the same measures at the same ages in order to be able to draw explicit comparisons between the United Kingdom and the United States (Petrill & Plomin, 2006). A comparison of ACE estimates for prereading skills and early literacy in United States, Australia, and Scandinavia generally found similar results across the countries but some evidence emerged in favor of the national curriculum hypothesis despite small sample sizes that limited the power to detect differences (Samuelsson et al., 2005).

Another, and conceptually more significant limitation of this *Monograph* is that it has not tackled an issue of great importance: the interplay between nature and nurture. Issues of gene–environment interaction and correlation interest us greatly (e.g., Asbury, Wachs, & Plomin, 2005; Plomin & Davis, 2006) and we believe that they will be important topics in relation to school environments and learning abilities and disabilities. However, our initial forays in this direction, mentioned above, have been disappointing. We found that school characteristics and children's perceptions of their school environment account for little variance in children's academic performance (Walker, Petrill, & Plomin, 2005; Walker & Plomin, 2006). We are currently attempting to improve our measures of children's perceptions of school environment by conducting interviews with 50 pairs of MZ twins for 10 consecutive school days in collaboration with David Almeida, who has developed the use of diary methods to assess daily stressors (Almeida, 2005).

###### SURPRISES

In this final section, we return to more general issues about nature and nurture that emerge from the research reported in this monograph, beginning with three surprises.

(1) *Substantial heritability and modest shared environment*. The results surprised us by showing such substantial heritability and such modest shared environmental influence for learning abilities in the early school years. Heritabilities are about 65% for teacher assessments based on U.K. National Curriculum criteria and about 55% for test data. Heritabilities for learning abilities are considerably greater than for general cognitive ability (about 35%). The similarity of results across domains, across ages, and across methods of assessment indicates the robustness of these findings.

The modest contribution of shared environment was just as surprising because the twins grew up in the same family, attended the same school, and were often taught by the same teacher in the same classroom. Across domains and across age, the average estimate of shared environment is about 15% for the NC ratings and about 20% for the test data. In research reported elsewhere we have found that more than 80% of the shared environment for NC ratings can be accounted for by socioeconomic status (Walker, Petrill, & Plomin, 2005). The rest of the shared environment was accounted for by school characteristics as measured using U.K. government statistics on variables such as class size and student–teacher ratio, authorized and unauthorized absence, average NC achievement level, and percentage of students eligible for free school meals (Walker, Petrill, & Plomin, 2005).

Nonshared environment accounted for more variance than shared environment—20% for NC ratings and 25% for test data. Again, we note that nonshared environment includes error of measurement. However, nonshared environment is not solely error of measurement, as can be seen in the longitudinal and multivariate analyses in Chapters V and VI. The longitudinal analyses in Chapter V indicate that the average nonshared environmental correlation is .24 from 7 to 10 years for NC teacher ratings of English and mathematics (Table 14). This suggests that the chances are about one in four that a nonshared environmental factor associated with learning abilities at 7 will also be associated with learning abilities at 10. In other words, an environmental factor at 7 years that makes one member of an MZ pair better at mathematics than the co-twin also makes that same MZ co-twin better at mathematics at 10 years. In addition, the multivariate analyses in Chapter VI yield an average nonshared environmental correlation of .42 for NC teacher ratings of English and mathematics (Table 16). Although it is possible that such nonshared environmental factors could involve correlated error, they are at least systematic in their effect and independent of genetic and shared environmental influences and thus warrant further investigation.

What environmental factors could make siblings, even MZ twins, different from one another in learning abilities? As mentioned earlier, research reported in this monograph adds two more pieces to the puzzle of nonshared environment: Not only do nonshared environmental influences on learning abilities make two children in the same family different from one another, they also make children at one age different from themselves at another age and they make children different across domains of learning.

Nearly all research attempting to identify specific sources of nonshared environment has focused on family environments rather than school environments and on personality and behavior problems rather than learning abilities. Nonetheless, such research should be informative for future research that will attempt to identify nonshared school environments that affect learning abilities. A meta-analysis of 43 papers relating differential family experience of siblings to differential outcomes concluded that “measured nonshared environmental variables do not account for a substantial portion of nonshared variability” (Turkheimer & Waldron, 2000, p. 78). Although another review interpreted these results more optimistically (Plomin, Asbury, & Dunn, 2001), the search for nonshared environments in school might best begin outside the family.

For example, peers have been nominated as an important candidate for nonshared environment as siblings in a family make their own individual ways in the world outside their family (Harris, 1998), and initial research appears to confirm this prediction (Iervolino et al., 2002). However, peers would not seem likely to be able to explain why nonshared environmental factors change so much from year to year, nor would peers easily explain why nonshared environmental factors differ from one academic subject to another. We thought that children's perceptions of their school environment might be better able to address these new pieces to the puzzle because children's perceptions could differ across time and across subjects. We have conducted research that suggests that children's perceptions of their school environment are a potent source of nonshared environmental experience in school (Walker & Plomin, 2006). However, the problem is that these nonshared environmental experiences hardly relate to nonshared environmental variance in academic achievement.

We also need to consider the possibility that chance contributes to nonshared environment in terms of random noise, idiosyncratic experiences, or the subtle interplay of a concatenation of events (Plomin, Asbury, & Dunn, 2001). Chance is the most obvious candidate for explaining the age-specific and subject-specific nature of nonshared environment in learning abilities. Nonetheless, our view is that chance is the null hypothesis and that systematic sources of nonshared environment need to be thoroughly examined before we dismiss it as chance. Moreover, chance might only be a label for our current ignorance about the environmental processes by which children—even pairs of MZ twins—in the same family and same classroom come to be so different. Using differences within pairs of MZ twins is a particularly powerful strategy for identifying nonshared environmental effects independent of genetics (Asbury, Dunn, Pike, & Plomin, 2003; Asbury, Dunn, & Plomin, 2006).

(2) *Similar results for boys and girls*. The results are similar for boys and girls as well as for same-sex and opposite-sex twins, suggesting that neither quantitative nor qualitative sex differences play an important role in the origins of individual differences in learning abilities.

(3) *Similar results for same teacher and different teachers*. Heritability estimates for NC teacher ratings were similar when the same teacher assessed both members of a twin pair and when different teachers assessed them, which provides strong support for the validity of the heritability estimates.

###### PUZZLES

Three unsolved puzzles also emerged from our analyses:

(1) *Why are teacher ratings of academic performance at 10 years more heritable than test scores?* For teacher ratings of reading at 10 years, heritability was 52%; for the web-based PIAT test of reading comprehension, heritability was 39%. For mathematics, heritability was 64% for teacher ratings and 49% for the web-based mathematics composite test score. Neither of these differences was significant; moreover, the heritability difference was not seen for reading at 7 years where heritabilities were 68% for NC ratings and 70% for TOWRE. Nonetheless, the heritability differences at 10 years warrant further consideration because they are substantial and consistent, especially if we find similar differences in the future when the twins are assessed again at 12 years.

Although teacher ratings and test scores at 10 years correlate about .50 phenotypically and about .60 genotypically, this leaves plenty of room for differences in the two types of measures. We explored this difference by comparing patterns of correlations with other variables in TEDS for teacher ratings versus test scores. For example, the heritability differences might be due to the possibility that teachers' year-long evaluation of children yield deeper insights into children's capabilities, including their appetites as well as their attitudes. However, in analyses of children's self-perceptions of reading and mathematics ability and their liking of these subjects (Spinath, Spinath, Harlaar, & Plomin, 2006), correlations with teacher ratings were not greater than correlations with test scores. We also considered the possibility that web-based tests show less heritability because they entail more artifactual shared environmental influence due to differences in the testing situation in the home such as more or less chaotic homes or more or less experience and comfort with computers. In support of this hypothesis, shared environment is slightly greater for reading and mathematics test scores (25% and 19%) than for teacher ratings (20% and 12%). However, in multiple regression analyses of home measures such as chaos, parental discipline and socioeconomic status, we again found similar correlations for teacher ratings and test scores.

Because reliability creates a ceiling for heritability estimates, a third possibility is that teacher ratings might be more reliable than test scores. This hypothesis is supported by the finding that nonshared environment, which includes measurement error, is slightly but significantly lower for teacher ratings than for test scores: 28% and 24% for NC teacher ratings of reading and mathematics and 36% and 32% for tests of reading and mathematics (Table 11). However, as discussed in Chapter II, our web-based test scores show high internal consistency, and test–retest reliability of the PIAT across 7 months was .66 in a study of 55 TEDS children. Moreover, a study of 30 TEDS children yielded a correlation of .92 between our web-based mathematics test and a standard version of the test administered in person 2 months later, which suggests that the web-based test is both highly reliable and valid (Haworth et al., 2007). A direct test of this hypothesis of differential reliability and stability will be possible in TEDS when the 10-year results for teacher ratings and test scores can be compared with similar measures that will be included in the 12-year assessment.

(2) *Why is the TOWRE measure of word recognition at 7 years significantly more heritable than PIAT reading comprehension at 10 years?* Heritability estimates are 70% for the TOWRE at 7 years and 39% for the PIAT at 10 years, a significant difference in heritability. We had expected the reverse pattern of results—that is, the TOWRE would be less heritable than the PIAT—based on our naïve assumption that early skill at reading words (TOWRE) is more a matter of exposure and training and that later reading comprehension (PIAT) involves “*g*” to a greater extent. The simplest explanation for the finding is that our assumptions were wrong and early word recognition is in fact much more heritable than later reading comprehension. At the level of genetic correlations as well, our assumptions about differences between the tests were wrong: the TOWRE and the PIAT are highly correlated genetically (.60), even though the tests assessed such apparently different cognitive processes and were administered 3 years apart.

The same two methodological issues discussed above could contribute to the difference in heritability between the TOWRE and PIAT. That is, differences in the testing situation in the home could contribute to the difference in heritability by increasing shared environmental variance for the web-based tests at the expense of heritability. Support for this hypothesis comes from the greater shared environment estimate for the web-based PIAT (.25) as compared with the telephone-administered TOWRE (.15). The other methodological hypothesis is that the web-based PIAT might be less reliable than the telephone-administered TOWRE. Support for this hypothesis comes from the greater nonshared environment estimate for the PIAT (.36) than for the TOWRE (.15).

A more interesting possibility is that, despite the genetic correlation of .60 between TOWRE at 7 years and PIAT at 10 years, different cognitive processes contribute to the two measures. The early stages of word recognition are known to be closely related to levels of phonological awareness, the knowledge that the sound of a word is made up of smaller pieces of sound (Snowling & Hayiou-Thomas, 2006). Learning to decode is therefore less related to “*g*” than later stages of reading that emphasize reading comprehension (Vanderwood, McGrew, Flanagan, & Keith, 2001). Several studies have shown that the heritability of phonological awareness at the beginning of school is approximately .6 (e.g., Hohnen & Stevenson, 1999; Petrill, Deater-Deckard, Thompson, DeThorne, & Schatschneider, 2006). This is much higher than the heritability of “*g*” (.36, .36, .41 at 7, 9, 10 years in our sample). And the genetic correlation between the TOWRE at 7 and “*g*” (.47) is lower than the genetic correlation between the PIAT at 10 and “*g*” (.63). In other words, word recognition might depend, more than comprehension, on a highly heritable specific foundational skill of phonological awareness.

In summary, we are again left with a puzzle. And, again, the 12-year TEDS assessment should help to solve the puzzle because the TOWRE and the PIAT, as well as other reading measures (although not phonological awareness), are included concurrently in the ongoing 12-year assessment.

(3) *Why is science at 10 years less heritable and more influenced by shared environment than English or mathematics?* In contrast to the heritabilities of 60% for NC English and 64% for NC mathematics at 10 years, the heritability of NC science is 48%. Shared environment estimates for English and mathematics are 20% and 12%, whereas for science the estimate is 27% (Table 11). These differences might not be reliable because they are not significant and they did not occur at 9 years. Nonetheless, throughout our longitudinal and multivariate genetic analyses (Chapters V and VI), science at 10 years often yields results that differ from results for English and mathematics. For example, our multivariate genetic analyses with “*g*” and NC ratings indicate that science performance at 10 years has more to do with “*g*” genetically than do English and mathematics.

The possibility that science performance is etiologically different from other academic subjects warrants further exploration because hardly any genetic research has addressed science performance as compared with the many studies of reading and an increasing number of studies of mathematics. Although science is a very broad and diffusely defined domain, we are especially interested in science as a way of understanding the world rather than as a body of facts. From little problems of daily life to problems in the field of cosmology, the scientific method—that is, posing logical, testable, and falsifiable hypotheses; testing the hypotheses as convincingly as possible; and interpreting the results reasonably—is the best way to solve problems, at least those problems that are amenable to empirical solutions. Getting children to see that the scientific method works in a practical way may help to counter the trend toward declining interest in science during the school years (Murphy & Beggs, 2003) and the general societal trend toward mysticism (Koch & Smith, 2006).

###### IMPLICATIONS

In a recent survey, more than 90% of parents and teachers perceive genetics to be at least as important as the environment for learning abilities and disabilities (Plomin & Walker, 2003). However, if there are any parents, teachers or policymakers who do not yet realize the important contribution that genetics makes to learning abilities and disabilities, these findings are important at this most rudimentary level of nature and nurture.

Even though teachers appear to recognize substantial genetic influence on academic achievement, there is a wide gap between education and genetics. The field of education scarcely acknowledges genetics despite the evidence for its importance, which is unfortunate because schools are the primary societal mechanism for fostering cognitive development (Rutter & Maughan, 2002). For example, a 2003 review of major educational psychology textbooks revealed that no text included more than three pages devoted to the topic of genetics (Plomin & Walker, 2003). Also, very few papers on genetics have been published in educational psychology journals.

Some of the reluctance to embrace genetics may be specific to the history and epistemology of education and educational psychology (Wooldridge, 1994). However, much of the reluctance is likely to involve general misconceptions about what it means to say that genetics is important (Rutter & Plomin, 1997). A key misconception is environmental nihilism, that is, if a disorder is heritable there is nothing that can be done about it environmentally (Sternberg & Grigorenko, 1999). The myth of environmental nihilism feeds into a related myth that finding genetic influence will serve to justify social inequality. We do not accept this view. Knowledge alone does not account for educational, societal, or political decisions—values are just as important in the decision-making process. We are aware that the relationship between knowledge and values is a complicated area of philosophy; here we are merely making the simple point that decisions, both good and bad, can be made with or without knowledge. For example, finding specific genes associated with reading disability obviously does not mean that we ought to put all available resources into educating those children with the most favorable genes and forget the rest of the children. Depending on our values, genetics could be used to argue for the opposite policy: We need to devote more resources to helping disadvantaged children. Whether or not our view of policymaking is naïve, surely it cannot be good for the science of education to pretend that genetic differences do not exist. And it may not be too Pollyannaish to hope that better policy decisions can be made with knowledge than without.

Even further back in the shadows is a general uneasiness about genetics in terms of our view of the essence of humanity. Are we not all created equal? The authors of the U.S. Declaration of Independence did not mean that we are all created identical—we obviously differ in height, for example. They clearly meant that in a democracy we should all be treated equally before the law and, more optimistically, that we should all have equal opportunities including educational opportunity (Husén, 1978). Indeed, if we were all identical there would be no need for a legal equality because its purpose is to ensure equality of treatment despite our differences (Pinker, 2002).

It would be a pity if the nature–nurture battles fought two decades ago in other areas of the behavioral sciences had to be refought in the field of education. By coming late to genetics, educational psychology can from the start embrace a more balanced position that acknowledges the importance of nature as well as nurture and uses genetic research to ask questions that go beyond heritability, such as the developmental and multivariate questions that are the focus of this monograph. Finding genetic influence will not denigrate the role of education; it will suggest new ways of thinking about effective education, such as recognizing that children create their own experience within the educational process in part on the basis of their genetic propensities.

Just as important as finding substantial genetic influence on learning abilities and disabilities is the finding that shared environment accounts for <20% of the variance. In contrast, research and discussion of the environmental origins of academic performance has focused almost entirely on family background and the school and classroom viewed as shared environmental effects. It is time to change that assumption. In addition, the finding that nonshared environment accounts for at least as much environmental variation as does shared environment opens up a new area of research that considers how children in the same school—even clones (MZ twins) in the same classroom—experience different environments.

The most important implications of these findings will come to the fore when specific genes are identified that contribute to the high heritability of learning abilities and disabilities. Although progress has been slow, recent developments in molecular genetics are promising (Plomin, 2005). For reading disability, for example, four candidate genes are currently the target of intense research (McGrath, Smith, & Pennington, 2006; Fisher & Francks, 2006). Reports are also beginning to appear of genes associated with normal variation in cognitive abilities (Plomin, Kennedy, & Craig, 2006). Although few educational and psychological researchers are likely to become involved in the quest to find genes associated with learning abilities and disabilities, when the genes are found they will be widely used in research as DNA risk indicators in much the same way that demographic risk indicators are currently used (Plomin & Walker, 2003). It should be emphasized that, like demographic risk indicators, genetic prediction will be probabilistic because there will be many genes of small effect size, as suggested by the QTL model described earlier.

Acceptance of genetic influence will come more readily because identifying specific genes provides evidence for genetic influence that is much more direct than the evidence provided by quantitative genetic research such as twin studies. Moreover, DNA has a unique causal status in that correlations between DNA differences and behavioral differences can only be explained causally in one direction: DNA differences cause behavioral differences. This causal status of DNA is unique in the sense that correlations involving other biological variables such as brain variables are just correlations that can be explained in either causal direction—behavioral differences can cause brain differences. However, variation in DNA sequence, which is the basis of heredity, is not changed by behavior, biology, or the environment.

Although finding specific genes associated with learning disabilities is unlikely to have much direct effect on teachers in the classroom confronted with a particular child with a learning disability, such findings will have far-reaching ramifications in terms of diagnosis, treatment, and prevention. Finding genes responsible for the high heritability of learning disabilities will lead to new diagnostic classifications that are based on etiology rather than symptomatology. As discussed earlier, two crucial examples emerged from the research described in this monograph: Learning disabilities are the quantitative extreme of the same genetic and environmental factors responsible for normal variation in learning abilities and the same set of genes influence most learning disabilities and abilities.

In terms of treatment, genes will be used clinically or educationally to the extent that response to treatment depends on genetic risk. This goal is part of a “personalized medicine” movement toward individually tailored treatments rather than treatments that are “one size fits all” (Abrahams, Ginsburg, & Silver, 2005). As noted earlier, the most important benefit of identifying genes that put children at risk for developing learning disabilities is that the causal nature of genes means that they can serve as an early-warning system. This should facilitate research on interventions that prevent problems, rather than waiting until problems are so severe that they can no longer be ignored. The goal of early intervention fits with a general trend toward preventative medicine. Because vulnerability to learning disabilities involves many genes of small effect, genetic engineering is unimaginable for learning disabilities; interventions will rely on environmental engineering, primarily educational interventions.

When genes associated with learning abilities and disabilities are found, the next step in research is to understand how these genes have their effect, called *functional genomics*. Functional genomics is usually considered in terms of the bottom-up agenda of molecular biology, which begins with the analysis of molecules in cells. However, the behavioral level of analysis is also useful for understanding how genes have their effect in relation to the development of the whole child, for example, in understanding interactions and correlations between genes and environment as they affect development and in leading to new diagnoses, treatments and interventions. The phrase *behavioral genomics* has been proposed to emphasize the importance of such top-down levels of analysis for understanding how genes have their effect on behavior (Plomin & Crabbe, 2000). Bottom-up and top-down levels of analysis of developmental pathways between genes and behavior will eventually meet in the brain. The grandest implication for science is that DNA will serve as an integrating force across diverse life sciences relevant to understanding learning abilities and disabilities.

## APPENDIX A NATIONAL CURRICULUM KEY STAGES 1 AND 2 TEACHER ASSESSMENT SCALE FOR ENGLISH

Teachers ticked one of five boxes to indicate the twins' level of attainment in terms of the TA scores; the criteria are listed below:

### KEY STAGE 1

#### Speaking and Listening

W. Not yet functioning at Level 1.

1. Pupils talk about matters of immediate interest. They listen to others and usually respond appropriately. They convey simple meanings to a range of listeners, speaking audibly, and begin to extend their ideas or accounts by providing some detail.

2. Pupils begin to show confidence in talking and listening, particularly where the topics interest them. On occasions, they show awareness of the needs of the listener by including relevant detail. In developing and explaining their ideas they speak clearly and use a growing vocabulary. They usually listen carefully and respond with increasing appropriateness to what others say. They are beginning to be aware that in some situations a more formal vocabulary and tone of voice are used.

3. Pupils talk and listen confidently in different contexts, exploring and communicating ideas. In discussion, they show understanding of the main points. Through relevant comments and questions, they show they have listened carefully. They begin to adapt what they say to the needs of the listener, varying the use of vocabulary and the level of detail. They are beginning to be aware of Standard English and when it is used.

4+. Speaking and listening are substantially more advanced than most pupils at Level 3.

#### Reading

W. Not yet functioning at Level 1.

1. Pupils recognize familiar words in simple texts. They use their knowledge of letters and sound–symbol relationships in order to read words and to establish meaning when reading aloud. In these activities they sometimes require support. They express their response to poems, stories and nonfiction by identifying aspects they like.

2. Pupils' reading of simple texts shows understanding and is generally accurate. They express opinions about major events or ideas in stories, poems, and nonfiction. They use more than one strategy, such as phonic, graphic, syntactic, and contextual, in reading unfamiliar words and establishing meaning.

3. Pupils read a range of texts fluently and accurately. They read independently, using strategies appropriately to establish meaning. In responding to fiction and nonfiction they show understanding of the main points and express preferences. They use their knowledge of the alphabet to locate books and find information.

4+. Reading is substantially more advanced than most pupils at Level 3.

#### Writing

W. Not yet functioning at Level 1.

1. Pupils' writing communicates meaning through simple words and phrases. In their reading or their writing, pupils begin to show awareness of how full stops are used. Letters are usually clearly shaped and correctly orientated.

2. Pupils' writing communicates meaning in both narrative and nonnarrative forms, using appropriate and interesting vocabulary, and showing some awareness of the reader. Ideas are developed in a sequence of sentences, sometimes demarcated by capital letters and full stops. Simple, monosyllabic words are usually spelt correctly, and where there are inaccuracies the alternative is phonetically plausible. In handwriting, letters are accurately formed and consistent in size.

3. Pupils' writing is often organized, imaginative, and clear. The main features of different forms of writing are used appropriately, beginning to be adapted to different readers. Sequences of sentences extend ideas logically and words are chosen for variety and interest. The basic grammatical structure of sentences is usually correct. Spelling is usually accurate, including that of common, polysyllabic words. Punctuation to mark sentences—full stops, capital letters, and questions marks—is used accurately. Handwriting is joined and legible.

4+. Writing is substantially more advanced than most pupils at Level 3.

### KEY STAGE 2

#### Speaking and Listening

1. Not yet functioning at Level 2.

2. Pupils begin to show confidence in talking and listening, particularly where the topics interest them. On occasions, they show awareness of the needs of the listener by including relevant detail. In developing and explaining their ideas they speak clearly and use a growing vocabulary. They usually listen carefully and respond with increasing appropriateness to what others say. They are beginning to be aware that in some situations a more formal vocabulary and tone of voice are used.

3. Pupils talk and listen confidently in different contexts, exploring and communicating ideas. In discussion, they show understanding of the main points. Through relevant comments and questions, they show they have listened carefully they begin to adapt what they say to the needs of the listener, varying the use of vocabulary and the level of detail. They are beginning to be aware of Standard English and when it is used.

4. Pupils talk and listen with confidence in an increasing range of contexts. Their talk is adapted to the purpose: developing ideas thoughtfully, describing events, and conveying their opinions clearly. In discussion, they listen carefully, making contributions and asking questions that are responsive to others' ideas and views. They use appropriately some of the features of Standard English vocabulary and grammar.

5. Speaking and listening are substantially more advanced than most pupils at Level 4.

#### Reading

1. Not yet functioning at Level 2.

2. Pupil's reading of simple texts shows understanding and is generally accurate. They express opinions about major evens or ideas in stories, poems, and nonfiction. They use more than one strategy, such as phonic, graphic, syntactic, and contextual, in reading unfamiliar words and establishing meaning.

3. Pupils read a range of texts fluently and accurately. They read independently, using strategies appropriately to establish meaning. In responding to fiction and nonfiction they show understanding of the main points and express preferences. They use their knowledge of the alphabet to locate books and find information.

4. In responding to a range of texts, pupils show understanding of significant ideas, themes, events, and characters, beginning to use inference and deduction. They refer to the text when explaining their views. They locate and use ideas and information.

5. Reading is substantially more advanced than most pupils at Level 4.

#### Writing

1. Not yet functioning at Level 2.

2. Pupils' writing communicates meaning in both narrative and nonnarrative forms, using appropriate and interesting vocabulary, and showing some awareness of the reader. Ideas are developed in a sequence of sentences, sometimes demarcated by capital letters and full stops. Simple words are usually spelt correctly, and where there are inaccuracies the alternative is phonetically plausible. In handwriting, letters are accurately formed and consistent in size.

3. Pupils' writing is often organized, imaginative, and clear. The main features of different forms of writing are used appropriately, beginning to be adapted to different readers. Sequences of sentences extend ideas logically and words are chosen for variety and interest. The basic grammatical structure of sentences is usually correct. Spelling is usually accurate, including that of common, polysyllabic words. Punctuation to mark sentences—full stops, capital letters, and questions marks—is used accurately. Handwriting is joined and legible.

4. Pupils' writing in a range of forms is lively and thoughtful. Ideas are often sustained and developed in interesting ways and organized appropriately for the purpose and the reader. Vocabulary choices are often adventurous and words are used for effect. Pupils are beginning to use grammatically complex sentences, extending meaning. Spelling, including that of polysyllabic words that conform to regular patterns, is generally accurate. Full stop, capital letters, and questions marks are used correctly, and pupils are beginning to use punctuation within the sentence. Handwriting style is fluent, joined, and legible.

5. Writing is substantially more advanced than most pupils at Level 4.

## APPENDIX B NATIONAL CURRICULUM KEY STAGES 1 AND 2 TEACHER ASSESSMENT SCALE FOR MATHEMATICS

Teachers are asked to indicate the description that corresponds to the pupil's progress by ticking the appropriate box, giving a score of W, 1, 2, 3, or 4+ as follows:

### KEY STAGE 1

#### Using and Applying Mathematics

W. Not yet functioning at Level 1.

1. Pupils use mathematics as an integral part of classroom activities. They represent their work with objects or pictures and discuss it. They recognize and use a simple pattern or relationship.

2. Pupils select the mathematics they use in some classroom activities. They discuss their work using mathematical language and are beginning to represent it using symbols and simple diagrams. They explain why an answer is correct.

3. Pupils try different approaches and find ways of overcoming difficulties that arise when they are solving problems. They are beginning to organize their work and check results. Pupils discuss their mathematical work and are beginning to explain their thinking. They use and interpret mathematical symbols and diagrams. Pupils show that they understand a general statement by finding particular examples that match it.

4+. Use and application of mathematics is substantially more advanced than most pupils at Level 3.

#### Number and Algebra

W. Not yet functioning at Level 1.

1. Pupils count, order, add, and subtract numbers when solving problems involving up to 10 objects. They read and write the numbers involved.

2. Pupils count the sets of objects reliably, and use mental recall of addition and subtractions facts up to 10. They begin to understand the place value of each digit in a number and use this to order numbers up to 100. They choose the appropriate operation when solving addition and subtraction problems. They use the knowledge that subtraction is the inverse of addition. They use mental calculation strategies to solve number problems involving money and measures. They recognize sequences of numbers, including odd and even numbers.

3. Pupils show understanding of place value in numbers up to 1,000 and use this to make approximations. They begin to use decimal notation and to recognize negative numbers, in contexts such as money and temperature. Pupils use mental recall of addition and subtraction facts to 20 in solving problems involving larger numbers. They add and subtract numbers with two digits mentally and numbers with three digits using written methods. They use mental recall of the 2–5, and 10 multiplication tables and derive the associated division facts. They solve whole-number problems involving multiplication or division, including those that give rise to remainders. They use simple fractions that are several parts of a whole and recognize when two simple fractions are equivalent.

4+. Understanding of number and algebra is substantially more advanced than most pupils at Level 3.

#### Shapes, Space, and Measures

W. Not yet functioning at Level 1.

1. When working with 2D and 3D shapes, pupils use everyday language to describe properties and positions. They measure and order objects using direct comparison, and order events.

2. Pupils use mathematical names for common 3D and 2D shapes and describe their properties, including numbers of sides and corners. They distinguish between straight and turning movements, understand angle as a measurement of turn, and recognize right angles in turns. They begin to use everyday nonstandard and standard units to measure length and mass.

3. Pupils classify 3D and 2D shapes in various ways using mathematical properties such as reflective symmetry for 2D shapes. They use nonstandard units, standard metric units of length, capacity and mass, and standard units of time, in a range of contexts.

4+. Understanding of shapes, space, and measures is substantially more advanced than most pupils at Level 3.

### KEY STAGE 2

#### Using and Applying Mathematics

1. Not yet functioning at Level 2.

2. Pupils select the mathematics they use in some classroom activities. They discuss their work using mathematical language and are beginning to represent it using symbols and simple diagrams. They explain why an answer is correct.

3. Pupils try different approaches and find ways of overcoming difficulties that arise when they are solving problems. They are beginning to organize their work and check results. Pupils discuss their mathematical work and are beginning to explain their thinking. They use and interpret mathematical symbols and diagrams. Pupils show that they understand a general statement by finding particular examples that match it.

4. Pupils are developing their own strategies for solving problems and are using these strategies both in working within mathematics and in applying mathematics to practical contexts. They present information and results in a clear and organized way. They search for a solution by trying out ideas of their own.

5. Use and application of mathematics is substantially more advanced than most pupils at Level 4.

#### Number and Algebra

1. Not yet functioning at Level 2.

2. Pupils count the sets of objects reliably, and use mental recall of addition and subtractions facts up to 10. They begin to understand the place value of each digit in a number and use this to order numbers up to 100. They choose the appropriate operation when solving addition and subtraction problems. They use the knowledge that subtraction is the inverse of addition. They use mental calculation strategies to solve number problems involving money and measures. They recognize sequences of numbers, including odd and even numbers.

3. Pupils show understanding of place value in numbers up to 1,000 and use this to make approximations. They begin to use decimal notation and to recognize negative numbers, in contexts such as money and temperature. Pupils use mental recall of addition and subtraction facts to 20 in solving problems involving larger numbers. They add and subtract numbers with two digits mentally and numbers with three digits using written methods. They use mental recall of the 2–5, and 10 multiplication tables and derive the associated division facts. They solve whole-number problems involving multiplication or division, including those that give rise to remainders. They use simple fractions that are several parts of a whole and recognize when two simple fractions are equivalent.

4. Pupils use their understanding of place value to multiply and divide whole numbers by 10 or 100. In solving number problems, pupils use a range of mental methods of computation with the four operations, including mental recall of multiplication facts up to 10 × 10 and quick derivation of corresponding division facts. They use efficient written methods of addition and subtraction and of short multiplication and division. They add and subtract decimals to two places and order decimals to three places. In solving problems with or without a calculator, pupils check the reasonableness of their results by reference to their knowledge of the context or to the size of the numbers. They recognize approximate proportions of a whole and use simple fractions and percentages to describe these. Pupils recognize and describe number patterns, and relationships including multiple, factor, and square. They begin to use simple formulae expressed in words. Pupils use and interpret coordinates in the first quadrant.

5. Understanding of number and algebra is substantially more advanced than most pupils at Level 4.

#### Shapes, Space, and Measures

1. Not yet functioning at Level 2.

2. Pupils use mathematical names for common 3D and 2D shapes and describe their properties, including numbers of sides and corners. They distinguish between straight and turning movements, understand angle as a measurement of turn, and recognize right angles in turns. They begin to use everyday nonstandard and standard units to measure length and mass.

3. Pupils classify 3D and 2D shapes in various ways using mathematical properties such as reflective symmetry for 2D shapes. They use nonstandard units, standard metric units of length, capacity, and mass, and standard units of time, in a range of contexts.

4. Pupils make 3D mathematical models by linking given faces or edges, draw common 2D shapes in different orientations on grids. They reflect simple shapes in a mirror line. They choose and use appropriate units and instruments, interpreting, with appropriate accuracy, numbers on a range of measuring instruments. They find perimeters of simple shapes and find areas by counting squares.

5. Understanding of shapes, space, and measures is substantially more advanced than most pupils at Level 4.

## APPENDIX C NATIONAL CURRICULUM KEY STAGE 2 (AGES 9 AND 10) TEACHER ASSESSMENT SCALE FOR SCIENCE

Teachers are asked to indicate the description that corresponds to the twin's progress by checking a box corresponding to one of the categories listed below:

### KEY STAGE 2

#### Scientific Enquiry

1. Not yet functioning at Level 2.

2. Pupils respond to suggestions about how to find things out and, with help, make their own suggestions about how to collect data to answer questions. They use simple texts, with help, to find information. They use simple equipment provided and make observations related to their task. They observe and compare objects, living things, and events. They describe their observations using scientific vocabulary and record them, using simple tables when appropriate. They say whether what happened was what they expected.

3. Pupils respond to suggestions and put forward their own ideas about how to find the answer to a question. They recognize why it is important to collect data to answer questions. They use simple texts to find information. They make relevant observations measure quantities, such as length and mass, using a range of simple equipment. Where appropriate, they carry out a fair test with some help, recognizing and explaining why it is fair. They record their observations in a variety of ways. They provide explanations for observations and for simple patterns in recorded measurements. They communicate in a scientific way what they have found out and suggest improvements in their work.

4. Pupils recognize that scientific ideas are based on evidence. In their own investigative work, they decide on an appropriate approach (e.g., using a fair test) to answer a question. Where appropriate, they describe, or show in the way they perform their task, how to vary one factor while keeping others the same. Where appropriate they make predictions. They select information from sources provided for them. They select suitable equipment and make a series of observations and measurements that are adequate for the task. They record their observations, comparisons and measurements using tables and bar charts. They begin to plot points to form simple graphs, and use these graphs to point out and interpret patterns in their data. They begin to relate their conclusions to these patterns and to scientific knowledge and understanding and to communicate them with appropriate scientific language. They suggest improvements in their work, giving reasons.

5. Understanding of scientific enquiry is substantially more advanced than most pupils at Level 4.

#### Life Processes and Living Things

1. Not yet functioning at Level 2.

2. Pupils use their knowledge about living things to describe the basic conditions (e.g., a supply of food, water, air, light) that animals and plants need in order to survive. They recognize that living things grow and reproduce. They sort living things into groups, using simple features. They describe the basis for their groupings (e.g., number of legs, shape of leaf). They recognize that different living things are found in different places (e.g., ponds, woods).

3. Pupils use their knowledge and understanding of basic life processes (e.g., growth, reproduction) when they describe differences between living and nonliving things. They provide simple explanations for changes in living things (e.g., diet affecting the health of humans or other animals, lack of light or water altering plant growth). They identify ways in which an animal is suited to its environment (e.g., a fish having fins to help it swim).

4. Pupils demonstrate knowledge and understanding of life processes and living things drawn from the key stage 2 or key stage 3 program of study. They use scientific names for some major organs of body systems (e.g., heart at key stage 2, the stomach at key stage 3) and identify the position of these organs in the human body. They identify organs (e.g., stamen at key stage 2, stigma, root hairs at key stage 3) of different plants they observe. They use keys based on observable external features to help them to identify and group living things systematically. They recognize that feeding relationships exist between plants and animals in a habitat, and describe these relationships using food chains and terms (e.g., predator and prey).

5. Understanding life processes and living things is substantially more advanced than most pupils at Level 4.

#### Physical Processes

1. Not yet functioning at Level 2.

2. Pupils know about a range of physical phenomena and recognize and describe similarities and differences associated with them. They compare the way in which devices (e.g., bulbs) work in different electrical circuits. They compare the brightness of color of lights, and the loudness or pitch of sounds. They compare the movement of different objects in terms of speed or direction.

3. Pupils use their knowledge and understanding of physical phenomena to link cause and effect in simple explanations (e.g., a bulb failing to light because of a break in an electrical circuit, the direction or speed of movement of an object changing because of a push or a pull). They begin to make simple generalizations about physical phenomena (e.g., explaining that sounds they hear become fainter the further they are form the source).

4. Pupils demonstrate knowledge and understanding of physical processes drawn from the key stage 2 or key stage 3 program of study. They describe and explain physical phenomena (e.g., how a particular device may be connected to work in an electrical circuit, how the apparent position of the sun changes over the course of a day). They make generalizations about physical phenomena (e.g., motion is affected by forces, including gravitational attraction, magnetic attraction, and friction). They use physical ideas to explain simple phenomena (e.g., the formation of shadows, sounds being heard through a variety of materials).

5. Understanding of physical processes is substantially more advanced than most pupils at Level 4.

## APPENDIX D

**Table:** Unadjusted Raw Means (Standard Deviations) for NC Measures and Test Scores at 7, 9, and 10 Years by Sex

	All	Boys	Girls
7 years			
NC English: speaking	2.14 (.62)	2.08 (.64)	2.19 (.60)
NC English: reading	2.21 (.67)	2.14 (.69)	2.28 (.65)
NC English: writing	1.98 (.61)	1.89 (.63)	2.07 (.59)
NC mathematics: using	2.07 (.63)	2.10 (.66)	2.05 (.59)
NC mathematics: number	2.18 (.61)	2.21 (.64)	2.16 (.58)
NC mathematics: shapes	2.13 (.60)	2.14 (.63)	2.13 (.56)
TOWRE: word	41.37 (16.66)	39.84 (17.06)	42.81 (16.15)
TOWRE: nonword	18.94 (11.88)	18.98 (12.18)	18.90 (11.60)
9 years			
NC English: speaking	3.05 (.67)	2.97 (.69)	3.12 (.65)
NC English: reading	3.11 (.76)	3.02 (.79)	3.20 (.72)
NC English: writing	2.84 (.74)	2.74 (.75)	2.94 (.71)
NC mathematics: using	2.93 (.73)	2.96 (.76)	2.90 (.69)
NC mathematics: number	3.00 (.72)	3.06 (.74)	2.97 (.69)
NC mathematics: shapes	3.00 (.68)	3.02 (.71)	2.98 (.65)
NC science: enquiry	2.95 (.64)	2.97 (.67)	2.94 (.61)
NC science: life	3.01 (.59)	3.02 (.62)	3.01 (.55)
NC science: physical	2.98 (.60)	3.00 (.63)	2.96 (.58)
10 years			
NC English: speaking	3.39 (.74)	3.31 (.77)	3.47 (.72)
NC English: reading	3.52 (.82)	3.44 (.84)	3.60 (.79)
NC English: writing	3.25 (.79)	3.13 (.81)	3.36 (.75)
NC mathematics: using	3.33 (.79)	3.38 (.82)	3.28 (.75)
NC mathematics: number	3.41 (.77)	3.46 (.81)	3.36 (.74)
NC mathematics: shapes	3.38 (.75)	3.42 (.78)	3.35 (.72)
NC science: enquiry	3.32 (.71)	3.33 (.74)	3.31 (.68)
NC science: life	3.41 (.68)	3.42 (.71)	3.41 (.66)
NC science: physical	3.37 (.69)	3.39 (.72)	3.36 (.66)
PIAT	.57 (.16)	.57 (.17)	.56 (.16)
Web mathematics	.77 (.16)	.78 (.16)	.76 (.16)

## APPENDIX E

**Table:** PHENOTYPIC CORRELATIONS FOR ENGLISH, MATHEMATICS, SCIENCE, AND “*g*” ACROSS AGES (7, 9, AND 10 YEARS)

	7-Year NC English	9-Year NC English	10-Year NC English	7-Year NC Mathematics	9-Year NC Mathematics	10-Year NC Mathematics	9-Year NC Science	10-Year NC Science	7- Year TOWRE	10- Year PIAT	7- Year *g*	9- Year *g*	10- Year *g*
7-year NC English	1												
9-year NC English	.63**	1											
10-year NC English	.62**	.68**	1										
7-year NC mathematics	.74**	.55**	.55**	1									
9-year NC mathematics	.54**	.74**	.59**	.58**	1								
10-year NC mathematics	.54**	.59**	.76**	.55**	.63**	1							
9-year NC science	.51**	.74**	.57**	.51**	.75**	.54**	1						
10-year NC science	.51**	.56**	.78**	.48**	.53**	.77**	.49**	1					
7-year TOWRE	.67**	.65**	.63**	.53**	.53**	.53**	.51**	.51**	1				
10-year PIAT	.42**	.43**	.47**	.34**	.38**	.36**	.35**	.37**	.44**	1			
7-year *g*	.41**	.41**	.41**	.39**	.41**	.39**	.38**	.39**	.41**	.36**	1		
9-year *g*	.38**	.40**	.40**	.38**	.41**	.41**	.37**	.36**	.37**	.45**	.42**	1	
10-year *g*	.37**	.42**	.41**	.37**	.40**	.38**	.35**	.37**	.31**	.55**	.40**	.55**	1

** =significant at .01 alpha level.
